# Dysregulated hematopoiesis in bone marrow marks severe COVID-19

**DOI:** 10.1038/s41421-021-00296-9

**Published:** 2021-08-04

**Authors:** Xin Wang, Yanling Wen, Xiaowei Xie, Yang Liu, Xiaohua Tan, Qingxian Cai, Yawen Zhang, Lin Cheng, Gang Xu, Shengyuan Zhang, Haiyan Wang, Lanlan Wei, Xian Tang, Furong Qi, Juanjuan Zhao, Jing Yuan, Lei Liu, Ping Zhu, Florent Ginhoux, Shuye Zhang, Tao Cheng, Zheng Zhang

**Affiliations:** 1 Institute for Hepatology, National Clinical Research Center for Infectious Disease, Shenzhen Third People’s Hospital; The Second Affiliated Hospital, School of Medicine, Southern University of Science and Technology, Shenzhen, Guangdong China; 2 CAS Key Laboratory of Pathogenic Microbiology and Immunology, Institute of Microbiology, Chinese Academy of Sciences (CAS), Beijing, China; 3 State Key Laboratory of Experimental Hematology and National Clinical Research Center for Blood Diseases, Institute of Hematology and Blood Diseases Hospital, Chinese Academy of Medical Sciences & Peking Union Medical College, Tianjin, China; 4 Center for Stem Cell Medicine and Department of Stem Cell & Regenerative Medicine, Chinese Academy of Medical Sciences and Peking Union Medical College, Tianjin, China; 5 Department of Oncology and Hematology, Shenzhen Third Peoples Hospital, Shenzhen, Guangdong China; 6 Department of Hepatology, Shenzhen Third Peoples Hospital, Shenzhen, Guangdong China; 7 Shenzhen Bay Laboratory, Shenzhen, Guangdong China; 8 Department for Infectious Diseases, Shenzhen Third People’s Hospital, Shenzhen, Guangdong China; 9 Singapore Immunology Network (SIgN), Agency for Science, Technology and Research (A*STAR), Biopolis, Singapore; 10 Shanghai Public Health Clinical Center, Fudan University, Shanghai, China; 11 Shenzhen Research Center for Communicable Disease Diagnosis and Treatment of Chinese Academy of Medical Science, Shenzhen, Guangdong China

**Keywords:** Transcriptomics, Haematopoietic stem cells

## Abstract

Severe coronavirus disease 2019 (COVID-19) is often indicated by lymphopenia and increased myelopoiesis; however, the underlying mechanism is still unclear, especially the alteration of hematopoiesis. It is important to explore to what extent and how hematopoietic stem cells contribute to the impairment of peripheral lymphoid and myeloid compartments in COVID-19 patients. In this study, we used single-cell RNA sequencing to assess bone marrow mononuclear cells from COVID-19 patients with peripheral blood mononuclear cells as control. The results showed that the hematopoietic stem cells in these patients were mainly in the G1 phase and prone to apoptosis, with immune activation and anti-viral responses. Importantly, a significant accumulation of immature myeloid progenitors and a dramatic reduction of lymphoid progenitors in severe cases were identified, along with the up-regulation of transcription factors (such as *SPI1, LMO4, ETS2, FLI1*, and *GATA2*) that are important for the hematopoietic stem cell or multipotent progenitor to differentiate into downstream progenitors. Our results indicate a dysregulated hematopoiesis in patients with severe COVID-19.

## Introduction

Coronavirus disease 2019 (COVID-19), which is caused by severe acute respiratory syndrome-coronavirus-2 (SARS-CoV-2), has become a global pandemic^1^. As of June 2021, over 179 million COVID-19 cases and 3.9 million deaths were confirmed world-wide (https://coronavirus.jhu.edu/map.html)^2^. The impact of the virus infection varies between different age groups. Old people, especially those with co-morbidities, are more likely to develop severe symptoms^3,4^. More importantly, previous studies have reported that COVID-19 may significantly affect the hematologic and immunologic systems, leading to lymphopenia^5,6^, thrombocytopenia^7,8^, coagulation disorders^9–12^, increase of HLA-DR^Low^ classical monocytes^8,13^, accumulation of immature/dysfunctional CD10^Low^CD101^–^CXCR4^+/–^ neutrophils, and disappearance of non-classical CD14^Low^CD16^High^ monocytes^14,15^ in the blood and lungs, especially in severe cases with COVID-19^8^.

In addition, abnormal interferon (IFN) production, hyper-inflammatory responses, cytokine storms, inefficient or delayed induction of neutralizing antibodies, and specific T cell responses may also be involved in the immunopathogenesis of SARS-CoV-2 infection^16–20^. However, the mechanisms underlying these changes are still underexplored with current phenomena being understood mainly by examining human peripheral blood or lung tissue^6,21–24^. Peripheral immune cells are derived from hematopoietic stem and progenitor cells (HSPCs) in bone marrow (BM)^25^. In a recent report, recombinant receptor-binding subdomain 1 of spike protein of SARS-CoV-2 (RBD-SD1) was used as probe to study the potential tropism of SARS-CoV-2 in 33 types of normal human tissues, and the results showed that over 80% of RBD-SD1 probe strongly interacted with BM cells^26^, suggesting the importance of understanding how COVID-19 affects the BM niche, especially the hematopoietic stem cell (HSC)^27^.

To further explore the mechanism, we used single-cell RNA sequencing (scRNA-seq) to analyze paired BM mononuclear cells (BMMCs) and peripheral blood mononuclear cells (PBMCs) from six COVID-19 patients, and comprehensively dissected HSPC subsets using specific lineage markers and a previously established reference dataset^28^. Interestingly, in severe COVID-19 patients, lymphoid-primed progenitor cells were dramatically depleted, which did not happen in mild cases, and the accumulation of immature granulocyte-monocyte progenitor cell (GMPs) were found in their BMMCs. Taken together, we concluded that dysregulated hematopoiesis in BM could mark severe COVID-19.

## Results

### Patient cohort

We enrolled six patients aged 40–70 years old, including three with mild COVID-19 (M1, M2, and M3) and three with severe COVID-19 (S1, S2, and S3) (Table 1), and downloaded BMMC scRNA-seq data of three HCs in the same age range from public database^29^ as control. The sample “B” (age 47) was renamed as healthy control 1 (short as “HC1”) in this study, the sample “F” (age 41) as “HC2”, and sample “H” (age 50) as “HC3”^29^. Mild COVID-19 was defined as those with mild clinical symptoms and not requiring mechanical ventilation, while severe COVID-19 as requiring hospitalization with a low- to high-flow oxygen (Table 1). BM samples from six COVID-19 patients were collected ~20 days after their hospitalization, and paired PBMC samples were collected 1 day before BM puncture, except that of patient M2, whose sample was collected 10 days before BM puncture.

**Table 1:** Clinical data of the enrolled patients.

	M1	M2		M3	S1	S2	S3
Severity	Mild	Mild	Mild		Severe	Severe	Severe
Gender	Male	Female		Female	Male	Male	Male
Age (years)	60 s	40 s		50 s	60 s	50 s	40 s
Bone marrow puncture (days after hospitalization)	21	14		28	24	11	18
Oxygen requirement at the date of sampling	Non	Non		Non	Yes Medium flow	Yes Low flow	Yes Medium flow
WBC^a^ (×10^9^ cells/L) (RR: 3.5–9.5 × 10^9^)	2.91	3.68		3.00	5.12	7.03	8.20
RBC^a^ (×10^12^ cells/L) (RR: 3.8–5.1 × 10^12^)	3.8	4.14		3.44	2.97	4.71	4.58
Platelet^a^ (×10^9^/L) (RR: 125–350 × 10^9^)	177	324		176	237	412	343
HGB^a^ (g/L) (RR: 115–150)	84	102		90	87	142	141
Immature granulocyte^a^, % (RR: 0–0.6)	0.00	0.30		0.90	7.00	1.10	1.00
Lymphocyte^a^ (×10^9^ cells/L) (RR: 1.1–3.2 × 10^9^)	0.59	1.02		1.04	0.60	1.60	1.60
Neutrophil^a^ (×10^9^ cells/L) (RR: 1.8–6.3 × 10^9^)	2.16	2.05		1.00	3.19	4.65	5.98
Neutrophil to lymphocyte ratio	3.66	2.01		0.96	5.32	2.90	3.74
Monocyte^a^ (×10^9^ cells/L) (RR: 0.1–0.6 × 10^9^)	0.24	0.48		0.21	0.82	0.72	0.51

*RR* reference range.

^a^Analysis by blood routine examination of peripheral blood cells, M1/M3/S1/S2-1 day before bone marrow puncture, M2-4 days before bone marrow puncture, S3-2 days before bone marrow puncture.

Among the six COVID-19 patients, M1, M3, S1, and S2 had travel history from Wuhan, while S3 took a train that started from Wuhan in January 2020. All six patients were tested positive for SARS-CoV-2 mRNA and showed symptoms of viral pneumonia at the admission of hospital. The S1 patient was identified as a severe case on the 2nd day after hospitalization, and relieved from severe disease on the 20th day. However, the anal/nasal swab of S1 was still tested positive for SARS-CoV-2 mRNA even on the 35th day. The S2 was recognized as a patient with severe COVID-19 at the day of admission, and relieved on the 11th day. While S3 became severely ill on the 6th day after hospitalization, and got out of severe illness at 17th day.

Routine blood cell tests (1–4 days before BM puncture) showed that the number of red blood cells (RBCs) observed in three patients (M1, M3, S1) dropped out of the normal range, and four patients (M1, M2, M3, S1) exhibited lower hemoglobin (HGB) levels (Table 1). The number of platelets in all patients stayed within the reference range (125–350 × 10^9^), except for S2 (412 × 10^9^). Notable increase in the number of immature granulocytes could be found in all three severe cases (S1–S3) and one mild case (M3). In addition, M1, M2, M3, and S1 had low level of lymphocytes according to the reference range (1.1–3.2 × 10^9^) (Table 1). Although the number of neutrophils from the six patients was within the reference range, the neutrophil/lymphocyte ratio was higher in patients with severe COVID-19 patients than in mild cases (Table 1). Further BM examination of the six COVID-19 patients (Table 2) revealed that the proportions of banded neutrophil in all COVID-19 patients were lower than the reference range. In contrast, the percentages of segmented neutrophil increased in M1 (14.5%), M2 (33.0%), S1 (43.5%), S2 (20.5%), and S3 (70.0%). Of note, the severe cases contained much more segmented neutrophils in BMMCs, and the proportion of total granulocytes in S1 and S3 was higher than the reference range. More importantly, the total erythrocytes jumped in M1 and M3, but dramatically decreased in S1 and S3 (Table 2).

**Table 2:** Bone marrow examination in six COVID-19 patients.

Cell types (%)	M1	M2		M3	S1	S2		S3
Promyelocyte (RR: 0.97–2.17)	1.50	–	–		–	1.50	–	
Neutrophil-myelocyte (RR: 4.45–8.53)	7.50	6.00		7.80	1.50	3.50		–
Neutrophil-metamyelocyte (RR: 5.93–9.87)	11.50	7.00		12.50	4.50	10.50		–
Banded neutrophil (RR: 20.22–27.20)	18.00	8.50		17.00	18.50	18.50		6.00
Segmented neutrophil (RR: 6.52–12.36)	14.50	33.00		10.30	43.50	20.50		70.00
Eosinophil-myelocyte (RR: 0.15–0.61)	0.50	–		–	–	–		–
Eosinophil-metamyelocyte (RR: 0.17–0.81)	1.00	–		0.50	–	–		–
Banded eosinophil (RR: 0.64–1.86)	0.50	–		–	–	0.50		–
Segmented eosinophil (RR: 0.25–1.47)	–	1.00		2.00	7.00	0.50		1.00
Prorubricyte (RR: 0.51–1.33)	2.00	1.00		1.30	–	2.50		–
Rubricyte (RR: 5.50–9.32)	12.50	5.50		4.50	0.50	9.50		–
Metarubricyte (RR: 8.39–13.11)	19.00	16.50		33.50	1.50	13.00		–
Mature lymphocyte (RR: 15.74–29.82)	9.00	17.50		9.50	15.00	14.50		18.00
Mature monocyte (RR: 0.00–3.00)	1.50	3.50		0.80	8.00	3.00		5.00
Mature plasma cell (RR: 0.00–0.71)	1.00	0.50		0.30	–	2.00		–
Total granulocyte (RR: 40.00–60.00)	55.00	55.50		50.20	75.00	55.50		77.00
Total erythrocyte (RR: 20.00–25.00)	33.50	23.00		39.20	2.00	25.00		0.00
Erythrocyte/nucleated cell ratio	0.50	0.30		0.64	0.02	0.33		0.00

*RR* reference range.

### Bone marrow cellular landscape in the studied population

**Fig. 1:**
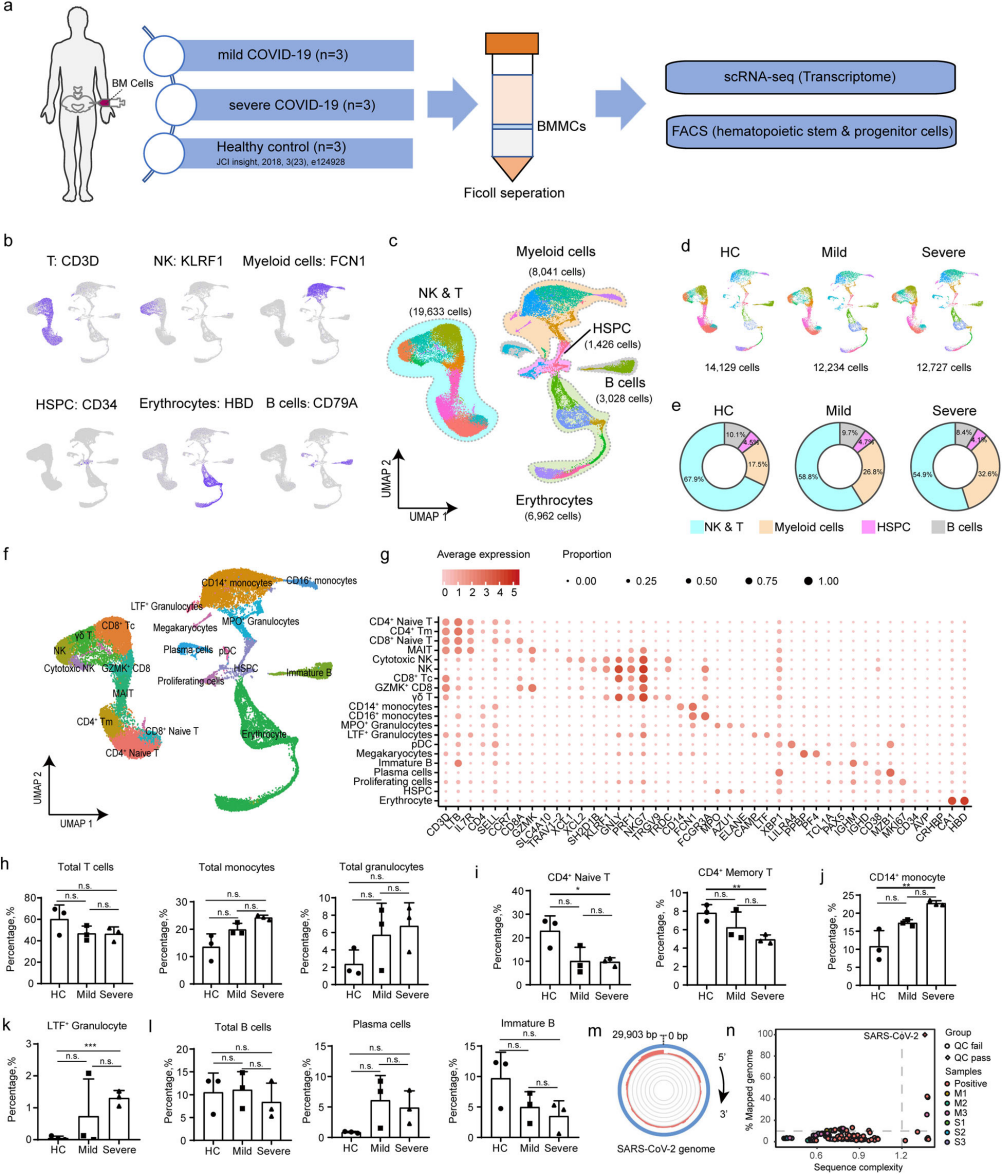
Characterization of BMMC compartments in COVID-19 patients. **a** Schematic diagram of the experimental design. ScRNA-seq of BMMCs derived from six COVID-19 patients and three age-matched HCs from public library were analyzed. **b** Feature plot of transcription activities for the marker genes of major cell types. **c** UMAP display of a total of 39,090 BMMCs. Five major cell types are indicated. **d** UMAP display of the distributions of BMMCs from HCs, mild, and severe patients. **e** Proportions of four major cell types (excluding erythrocytes) in BMMCs for HCs, mild, and severe patients. **f** UMAP display of 20 clusters. **g** Heatmap showing the relative expression of marker genes for each cell type. Colors indicate expression, while the size of the circles represents the proportion of expressed cells. **h** Proportions of total T cells, total monocytes, and total granulocytes in HCs, mild, and severe patients. **i** Fraction of CD4^+^ naïve T cells and CD4^+^ memory T cells among total cells. **j** Percentage of CD14^+^ monocyte among total cells. **k** Proportion of LTF^+^ granulocytes among total cells. **l** Ratio of total B cells, plasma cells, and immature B cells among total cells. **m** The aligned reads of the BMMC scRNA-seq dataset to the SARS-CoV-2 genome. The blue circle represents the genome of SARS-CoV-2, while red and gray circles represent positive control (previously reported BALF scRNA-seq dataset from COVID-19 patients) and BMMC samples, respectively. **n** Viral-Track analysis of the BMMCs scRNA-seq dataset. Only the positive control dataset (scRNA-seq data from BALF of COVID-19 patients^6^) showed QC passed (diamond dot) signals of SARS-CoV-2. *P* values for pairwise comparisons were calculated, unpaired two-sided Student’s *t-*test, *P < 0.05, **P < 0.01, ***P < 0.001, n.s., not significant.

We used Ficoll-based purification to isolate BMMCs from BM punctures for scRNA-seq on droplet-based single-cell platform (Fig. 1a). Public BMMCs scRNA-seq data of three age-matched healthy subjects were used as controls (Fig. 1a)^29^. Single-cell transcriptomes data obtained from 32,042 BMMCs that derived from COVID-19 patients were analyzed together with 16,330 BMMCs from HC, with averaged Mean Reads per Cell and a Median Genes per Cell being 123,494 and 1207 respectively (Supplementary Table S1). The clustering analysis was conducted by Uniform Manifold Approximation and Projection (UMAP), and five major cell types were identified, namely, natural killer (NK) & T cells (*KLRF1, CD3D*), myeloid cells (*FCN1*), HSPC (*CD34*), B cells (*CD79A*), and erythrocytes (*CA1, HBD*) (Fig. 1b, c). UMAP plot corresponding to each group/sample (Fig. 1d and Supplementary Fig. S1a) showed similar cellular compositions, suggesting high data integration quality and low batch effect. Then, we compared COVID-19 patients and controls in terms of the percentages of the four major cell types (Fig. 1e), and found higher proportion of myeloid cells (26.8% in mild patients, 32.6% in severe cases, vs 17.5% in controls), while lower proportion of NK & T lymphocytes (58.8% in mild patients, 54.9% in severe cases, vs 67.9% in controls) in COVID-19 patients. The proportions of HSPC and B cells in the two groups were comparable (Fig. 1e).

Referring to canonical cell-specific markers, the five major types could be further separated into 20 distinct cell clusters (Fig. 1f, g), including CD4 naïve T (*CD4, LTB*), CD8 naïve T (*CD8A, SELL*), MAIT (*SLC4A10, TRAV1–2*), cytotoxic NK (*XCL1, KLRF1*), NK (*KLRF1, NKG7*), CD8 cytotoxic T (“CD8 Tc”, *CD8A, GNLY*), GZMK CD8 (*CD8A, GZMK*), γδ T (*TRGV9, TRDC*), CD14 monocyte (*CD14, FCN1*), CD16 monocyte (*FCGR3A, FCN1*), MPO granulocyte (*MPO, AZU1*), LTF granulocyte (*CAMP, LTF*), pDC (*LILRA4*), megakaryocyte (*PPBP, PF4*), immature B cell (*TCL1A, PAX5*), plasma cells (*CD38, MZB1*), proliferating cells (*MKI67*), HSPC (*CD34, AVP, CRHBP*), and erythrocyte (*CA1, HBD*) (Fig. 1g). Possibly as a result of potential contamination of blood cells during the BM aspiration process, decreased number of BM T cells and increased number of monocytes/granulocytes were observed in patients with COVID-19 in this study compared to HC, especially in those with severe disease (Fig. 1h), which was consistent with previously reported peripheral changes^14,15,21,23^. In addition, the decline in T cells (Fig. 1h) was primarily due to the reduced CD4^+^ naïve T cells (*P* = 0.03 in severe vs HC, *P* = 0.06 in mild vs HC, Fig. 1i) and CD4^+^ memory T cells (*P* = 0.01 in severe vs HC, *P* = 0.22 in mild vs HC, Fig. 1I); whereas the percentages of other T cell subsets were comparable (Supplementary Fig. S1e–h). In addition, no significant change (*P* = 0.98 in severe vs HC, *P* = 0.46 in mild vs HC) was observed from the proportion of NK cells (Supplementary Fig. S1i, j). Regarding myeloid cells, the percentage of CD14^+^ monocytes in patients with severe COVID-19 was significantly higher than that in HC (*P* = 0.01, Fig. 1j). No HLA-DR^Low^ monocyte was found in BMMCs from COVID-19 patients, but an immature-like LTF^+^ granulocyte witnessed significant increase in patients with severe COVID-19 (Fig. 1k, *P* = 0.001 in severe vs HC, *P* = 0.37 in mild vs HC). Notably, the proportion of total B cells were comparable among all groups (Fig. 1l), but increased number of plasma cells (*P* = 0.06 in severe vs HC, *P* = 0.09 in mild vs HC) and decreased number of immature B cells (*P* = 0.10 in severe vs HC, *P* = 0.18 in mild vs HC) were observed in COVID-19 patients, although not statistically significant (Fig. 1l). In addition, no significant difference was found regarding the proportion of proliferating cells (Supplementary Fig. S1b), megakaryocytes (Supplementary Fig. S1c), or pDC (Supplementary Fig. S1d) between COVID-19 patients and HC.

Collectively, these data showed increased number of myeloid cells, decreased number of T cells, and a comparable level of HSPCs in the BM of COVID-19 patients when compared to HC.

### No infection of SARS-CoV-2 in the BMMCs of COVID-19 patients

There was no difference in the expression of *ACE2, NRP1*, and *TMPRSS2* (SARS-CoV-2 receptors^30^) between BMMCs of HC (Supplementary Fig. S1k) and those of COVID-19 patients (Supplementary Fig. S1l–m). *BSG* slightly elevated in most BMMCs from COVID-19 patients when compared with the HC. Additionally, no SARS-CoV-2 genome was identified in BMMCs by reads mapping (Fig. 1m) or Viral-Track algorithm^31^ (Fig. 1n), while bronchoalveolar lavage fluid (BALF) from COVID-19 patients^6^, used as control, was tested positive.

Both higher levels of *ANPEP* in monocytes and granulocytes and higher levels of *DPP4* in T cells and dendritic cells were observed in COVID-19 patients compared with HC (Supplementary Fig. S1k–m). In addition, the transcriptomic level of *ST6GAL1* and *ST3GAL4* also increased in the BMMCs from COVID-19 patients (Supplementary Fig. S1–m). By Viral-Track analysis, although no evidence of viral infection was found in BMMCs of COVID-19 patients (Fig. 1n), patient M1, M3, and S3 were tested positive for respiratory syncytial virus RNA, herpes simplex virus, and EBV DNA, respectively, suggesting that co-infection of other pathogens should not be ignored in COVID-19.

### Characterization of HSPCs by cell-type-specific markers and a curated reference database

**Fig. 2:**
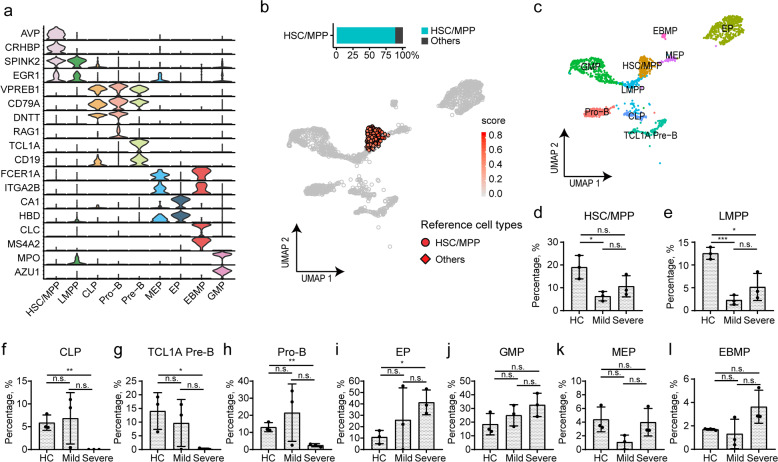
Annotation of HSPCs by cell-specific markers and immunophenotype-based reference dataset. **a** Violin plots show the expression of cell-specific markers for nine HSPC clusters. **b** Label transfer of HSC/MPP population according to an immunophenotype-based reference dataset. **c** UMAP displays the distribution of nine cell clusters of HSPCs. **d**–**l** The proportion of nine clusters in HSPCs from HC and COVID-19 patients. *P* values for pairwise comparisons were calculated, unpaired two-sided Student’s *t-*test, *P < 0.05, **P < 0.01, ***P < 0.001, n.s., not significant.

The depletion of T cells and the increase of myeloid cells in patients with COVID-19 have been reported^5,15,21,23^. Considering that no direct SARS-CoV-2 infection was observed in BMMCs, we hypothesized that an altered differentiation of HSPCs might have occurred during this process. Therefore, we stratified *CD34*^+^ cells from BMMCs and conducted a re-cluster analysis to precisely map HSPC heterogeneity. Nine progenitor cell clusters (Fig. 2a–c) were identified, including hematopoietic stem cell/multipotent progenitor cell (HSC/MPP, markers: *AVP, CRHBP*), lymphoid-primed multipotent progenitor (LMPP, markers: *SPINK2, EGR1*), megakaryocyte-erythrocyte progenitor cell (MEP, markers: *FCER1A, ITGA2B, HBD*), common lymphoid progenitor cell (CLP, markers: *DNTT, SPINK2*), eosinophil/basophil/mast progenitor cell (EBMP, markers: *CLC, MS4A2*), GMP (markers: *MPO, AZU1*), erythrocyte progenitor (EP, markers: *CA1, HBD*), Pre-B (markers: *TCL1A, CD19*), and Pro-B (markers: *VPREB1, RAG1*).

To further validate the naming, we conducted cross-reference for the nine clusters with a previously established scRNA-seq dataset of immunophenotype-guided HSPCs^28^, and the transferred cell-type annotation to our BMMCs dataset. We found that 88.6% of HSC/MPP cells (Fig. 2b), 50.6% of LMPP (Supplementary Fig. S2a), 76.9% of CLP (Supplementary Fig. S2b), and 55.9% of MEP (Supplementary Fig. S2c) could be consistently annotated by the reference dataset. Even though LMPP and MEP had relatively lower matching rates with the reference dataset, the majority of the unmatched LMPP and MEP was annotated as HSC/MPP (27.9% and 37.3%, respectively) (Supplementary Fig. S2a, c), suggesting an earlier change in transcriptome expression compared with surface marker when HSC was differentiated into LMPP or MEP.

Then we examined the proportion of each HSPC cluster and found significant decrease of HSC/MPP in patients with mild COVID-19 when compared with HC (Fig. 2d, *P* = 0.02), and slight decrease in patients with severe COVID-19 (Fig. 2d, *P* = 0.10). Moreover, a marked decrease could be found in the proportions of LMPP (Fig. 2e), CLP (Fig. 2f), TCL1A pre-B cells (Fig. 2g), and Pro-B cells (Fig. 2h) from COVID-19 patients with severe cases when compared to the HC group (Fig. 2e–h), while only the proportion of LMPP was significantly decreased in mild cases (Fig. 2e). In contrast, the proportion of EP (Fig. 2i) increased in patients with severe COVID-19, while no difference was found in that of MEP and EBMP (Fig. 2k, l). In summary, the lymphoid progenitors decreased significantly, while EP increased significantly in patients with severe COVID-19. In addition, the proportions of GMP slightly rose in those mild or severe cases when compared with HC (Fig. 2j).

### HSC/MPP is immune activated, non-proliferating, and prone to cell death in COVID-19 patients

**Fig. 3:**
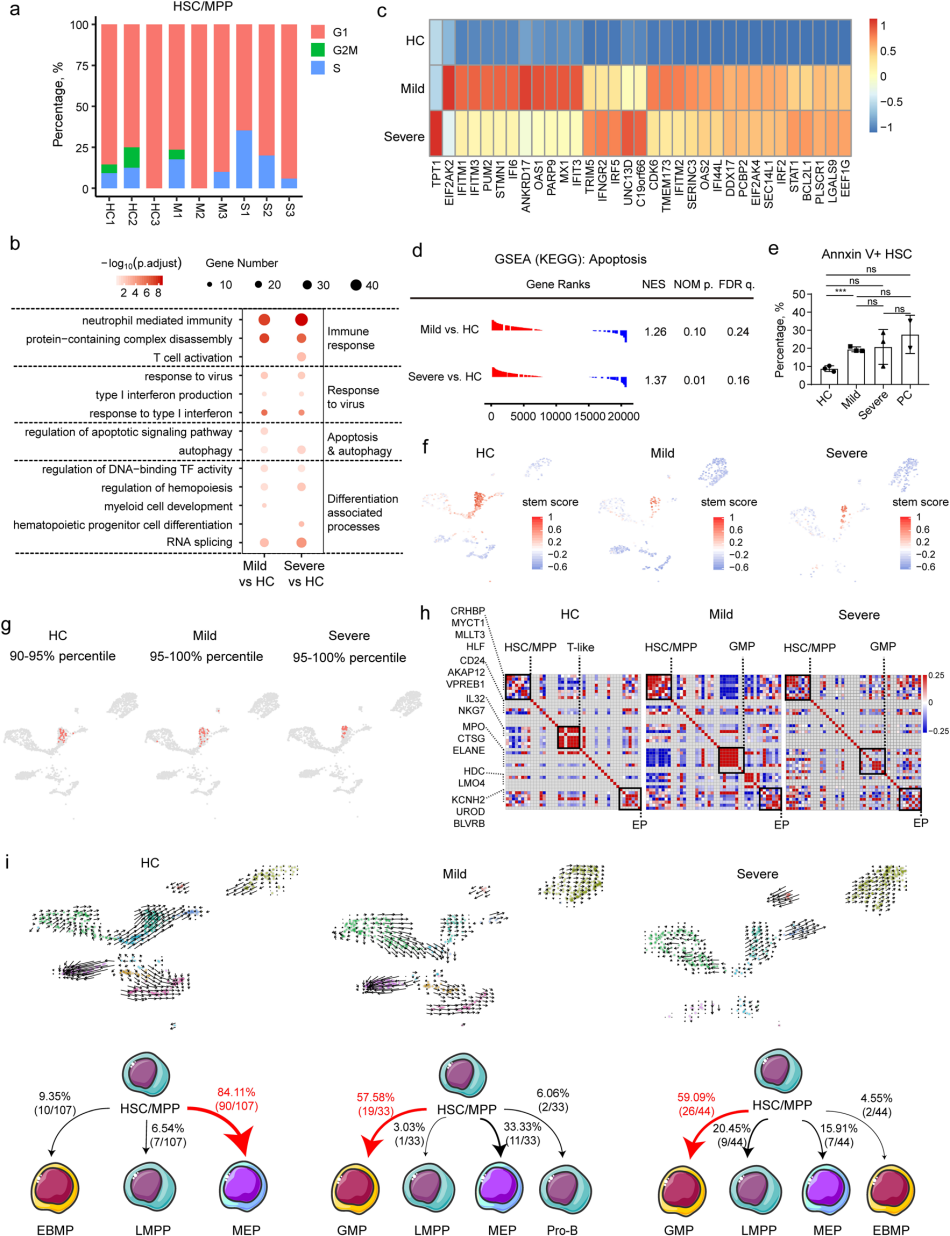
Perturbed differentiation tendency of HSC/MPP in COVID-19 patients. **a** Percentages of G1, S, and G2M phase for HSC/MPP population from HC and COVID-19 patients. **b** Enriched GO terms of up-regulated genes in the HSC/MPP population from COVID-19 patients. Three pathways were clustered into immune response-associated pathways, three were identified associated with virus response, two were related to apoptosis and autophagy, and five pathways participated in the processes of differentiation. **c** Heatmap showing the significant different expression patterns of IFN-stimulated genes in the HC, mild, and severe groups. **d** GSEA analysis of transcriptome comparisons in HSC/MPP shows that the KEGG pathway “Apoptosis” (Entry ID: hsa04210) was enriched in COVID-19 patients vs controls. **e** Proportion of Annexin V^+^ HSC in total HSC with flow cytometry. Results were compared among the HC, mild group, and severe group and two positive controls (PC). **f** Stemness signature scores of HSPCs from HC, mild, and severe patients projected onto 2c graph. Red color represents a higher stem score. **g**, **h** Gene pairwise Spearman correlation within the 90%–95% (HC) and 95%–100% (Mild group, Severe group) of the stem score. **g** The projection of corresponding cells onto the core model with corresponding percentile. **h** The heatmap of gene pairwise Spearman correlation. **i** RNA velocity analysis of HSPC cells among the HC, mild, and severe group. Upper panel projects the velocity field onto the UMAP plot of HSPC in the three groups. Lower panel shows the numbers and ratios of predicted differentiation endpoint of HSC/MPPs in the three groups.

As described above, the lymphoid progenitors were depleted in patients with severe COVID-19, suggesting that the perturbations of the lymphoid and myeloid compartments may be attributed to abnormal differentiation of upstream HSC/MPPs. Therefore, we first investigated whether HSC/MPPs were active or quiescent. Interestingly, gene expression analysis revealed that most HSC/MPPs from both controls and COVID-19 patients stayed in the G1 phase (Fig. 3a), a quiescent/non-proliferating state. Next, differentially expressed genes (DEGs) in the HSC/MPPs from COVID-19 patients and HC were analyzed (Supplementary Table S2), and gene ontology (GO) analysis of the DEGs of HSC/MPPs demonstrated enrichment of “neutrophil-mediated immunity” (Fig. 3b) and type I IFN-related pathway (Fig. 3b, c) in COVID-19 patients, indicating an immune activated status of HSC/MPP cells. Notably, autophagy (Fig. 3b), necroptosis (Supplementary Fig. S2d–f), and apoptosis pathway (Fig. 3b, d) were also significantly up-regulated in COVID-19 patients compared with HC. The proportion of apoptotic Annexin V^+^ HSCs in total Lin^–^ D34^+^CD38^–^ D45RA^–^CD90^+^CD49f^+^ HSCs from COVID-19 patients was higher than in HC, but lower than in samples collected from patients diagnosed with avascular necrosis of the femoral head and co-infected with human immunodeficiency virus or hepatitis virus, which worked as positive control (Fig. 3e and Supplementary Fig. S2g–k). More importantly, the biological processes related to myeloid cell development and hematopoietic progenitor cell differentiation were significantly up-regulated in COVID-19 patients (Fig. 3b), including “regulation of DNA-binding TF activity”, “regulation of hemopoiesis”, “myeloid cell development”, “hematopoietic progenitor cell differentiation”, and “RNA splicing”. These results suggested that the differentiation tendency of HSC/MPP cells might have been altered in COVID-19 patients.

### The differentiation of HSC/MPP cells from COVID-19 patients is changed at transcriptional level

HSC/MPPs gradually lose their stemness alongside their differentiation trajectory^32,33^. Here, we calculated stemness score^32^ (see Materials and methods section) for each single HSPC, and found that HSPCs with a high stemness score from HC outnumbered those from COVID-19 patients (Fig. 3f). Moreover, the HSC/MPPs from COVID-19 patients have lower stemness score when compared with those from HC (Supplementary Fig. S2l). Then, to determine the earliest turning point at which HSC/MPP exited from their naïve status, we ranked HSC/MPP according to their stemness scores, and stratified them every five percentiles and followed the expression pattern of lineage-specific markers (Supplementary Table S3 and Fig. S3a–f). Results showed that, in HC group, egress of HSC/MPP from stem cell status started at ~90–95% and expressed lymphoid associated gene module (e.g., *IL32* and *NKG7*) and erythrocyte progenitor associated gene module (e.g., *HBD*), suggesting lymphoid and erythroid differentiation potential of HSC/MPP in HC (Fig. 3g, h). In contrast, first five percentiles of HSC/MPPs from COVID-19 patients started to express gene modules associated with GMP and EP, rather than lymphoid compartments (Fig. 3g, h).

To further verify these results, RNA velocity algorithm was applied to model the differentiation trajectory of HSPCs^33^. The calculated velocity was projected onto the UMAP plots of HSPCs from all three studied groups (Fig. 3i, upper panel and Supplementary Fig. S3g), and each sample (Supplementary Fig. S3h–j). As expected, the velocity-arrow of HSC/MPPs primarily pointed to MEPs in HC, consistent with the cognition that HSC cells have a megakaryocyte-priming potential. In contrast, the HSC/MPPs cells from COVID-19 patients exhibited an attenuated “velocity” (differentiation potential) to MEPs, suggesting an altered differentiation tendency in these cells. Moreover, the proportions of differentiation endpoints of HSC/MPPs in the three groups were investigated. Results showed that 84.1% of HSC/MPPs from HC were predicted to preferentially differentiate into MEP cells (Fig. 3i, bottom left), while most of the HSC/MPPs from patients with mild (57.6%) and severe (59.1%) COVID-19 were predicted to preferentially differentiate into GMPs (Fig. 3i, bottom middle and bottom right). Although HSC/MPPs did not directly differentiate into EPs, MEPs from COVID-19 patients showed much stronger tendency to differentiate into EPs than in HC. The HSC/MPPs from mild COVID-19 cases kept the potential of differentiating into pro-B cells, while the cells from severe cases were prone to differentiate into myeloid-associated lineages, including GMPs, EBMPs, and LMPPs. These results revealed that the HSC/MPPs from COVID-19 patients preferentially differentiated into GMP, rather than the lymphoid progenitors (Fig. 3h, i). This agitation may partially contribute to the aforementioned decrease of LMPPs, CLPs, Pro-B, Pre-B and the increase of GMPs and EPs in patients with COVID-19, particularly in those severe cases.

### Up-regulation of megakaryocyte progenitor and granulocyte progenitor-priming transcription factors in the HSC/MPP of COVID-19 patients

Transcriptional factors (TFs) work as the driver of the differentiation process of HSC/MPPs. To explore whether lineage-primed TFs were affected in HSC/MPP cells from COVID-19 patients, we examined canonical lineage-priming TF modules, including Pre-B-priming TFs (namely *PAX5, EBF1, ID3*), megakaryocyte progenitor cells (MkP)-priming TFs (namely *ETS2, FLI1, GATA2, PBX1*), granulocyte progenitor (GP) priming-TFs (namely *SPI1, LMO4*), and MDP-priming TFs (namely *IRF7, IRF8*)^34–36^.

**Fig. 4:**
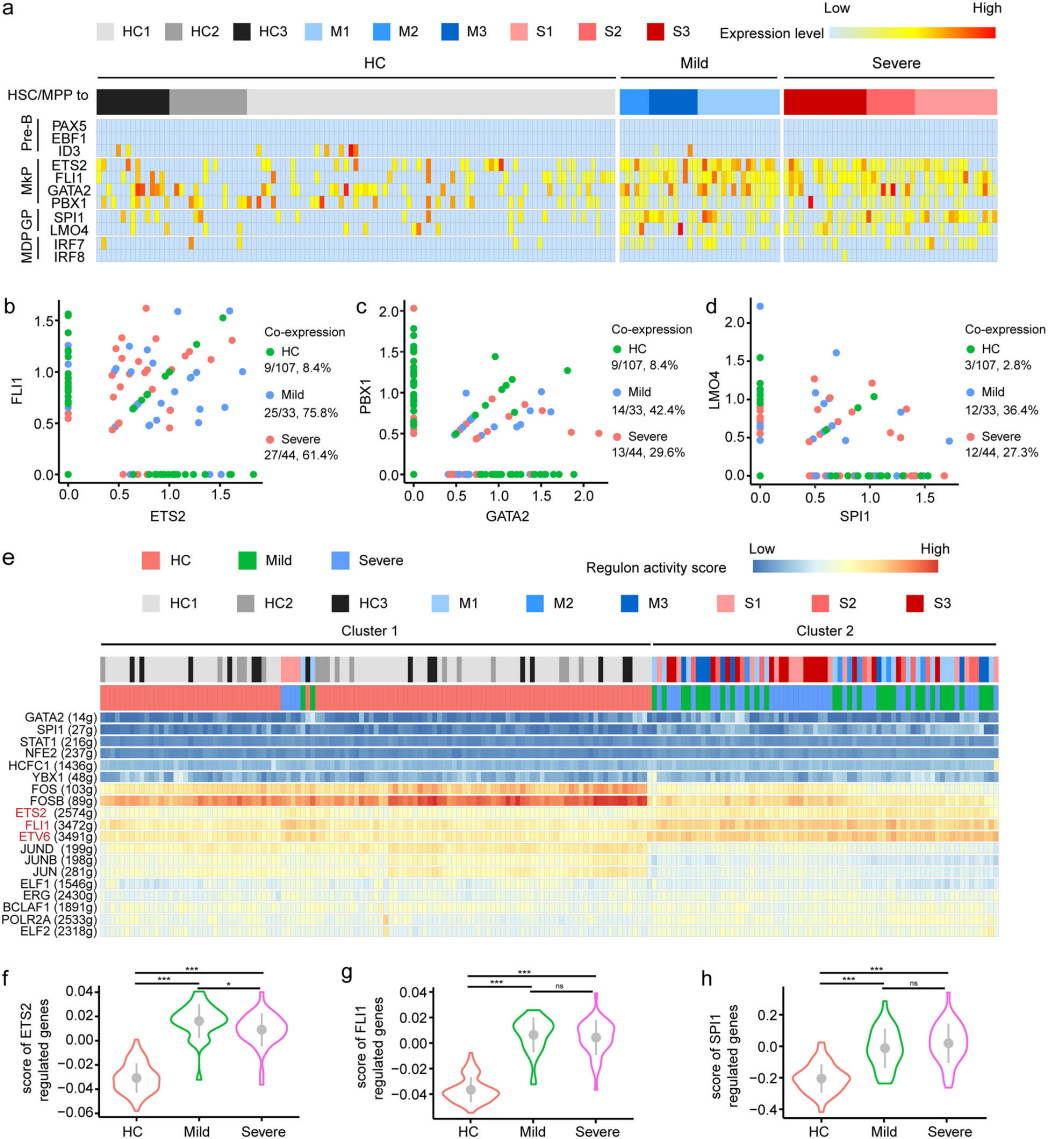
Transcriptional regulation underlies HSC/MPP from COVID-19 patients. **a** Heatmap displays the expression levels of lineage-priming TFs for HSC/MPP cells from HCs, mild, and severe patients. Five gene modules involved in hematopoiesis differentiation are presented, including pre-B (*PAX5, EBF1*, and *ID3*), MkP (*ETS2, FLI1, GATA2*, and *PBX1*), GP (*SPI1, LMO4*), and MDP (*IRF7, IRF8*). **b**–**d** Co-expression of selected TFs was assessed across different groups. **e** Heatmap showing the regulon activity scores (RASs) of lineage-related TFs for HSC/MPP cells from HCs, mild, and severe patients. 184 HSC/MPP cells were arranged by unsupervised clustering according to their regulon activities. **f**–**h** Score of specific TF-regulated genes in three groups.

In all, 8 out of 107 HSC/MPP cells in HC and 2 out of 77 HSC/MPP cells in COVID-19 patients with mild disease expressed *ID3*, but not in severe cases, although *PAX5* and *EBF1* were not identified (Fig. 4a). Regarding the MkP-priming TFs, significant co-expression of *ETS2-FLI1-GATA2-PBX1* and *SPI1-LMO4* was observed in the HSC/MPPs from COVID-19 patients (Fig. 4a), with 75.8% and 61.4% of HSC/MPPs co-expressing *ETS2* and *FLI1* in the mild and severe cases, respectively, compared with 8.4% in HC (Fig. 4b). 42.4% and 29.6% of HSC/MPPs co-expressing *GATA2* and *PBX1* were observed in the mild and severe groups, respectively, compared with 8.4% in HC (Fig. 4c). The co-expression of *SPI1* and *LMO4* in HSC/MPPs was 36.4% and 27.3% in mild and severe groups, respectively, compared with 2.8% in controls (Fig. 4d). Regarding MDP-priming TFs, only five of 107 HSC/MPPs (4.7%) from HC expressed *IRF7*, while 36.4% (12/33) of mild cases and 29.6% (13/44) of severe cases expressed *IRF7* (Fig. 4a).

To further investigate the transcriptional regulation, we analyzed gene regulatory networks of HSC/MPPs using an unsupervised algorithm named SCENIC^37^. Interestingly, the HSC/MPP cells were separated into two major subsets, namely cluster 1 and cluster 2. Cluster 1 contained all the 107 HSC/MPPs from the HC but only two HSC/MPPs from mild cases and four HSC/MPPs from severe COVID-19 cases, and the other 31 cells in mild cases and 40 cells in severe cases were clustered into cluster 2 unsupervisedly, indicating marked difference between regulatory TF landscapes of the HSC/MPPs from COVID-19 patients and those from HC (Fig. 4e and Supplementary Fig. S4a). Consistently, the regulons including *GATA2*, *SPI1*, *ETS2*, *FLI1*, and *ETV6* were activated in COVID-19 patients (Fig. 4e). In addition, we conducted score analysis to examine the transcriptional level of TF-downstream target genes, and identified substantial increase in scores of *ETS2*, *FLI1*, and *SPI1* in COVID-19 patients compared with controls (Fig. 4f–h and Supplementary Fig. S4d–f). In comparison, the scores of *GATA2* significantly increased only in HSC/MPPs from patients with severe COVID-19, rather than in those from mild cases when compared with HC (Supplementary Fig. S4b, g). In addition, no significant difference was found between mild and severe groups, except the *ETS2* (Fig. 4f). In contrast, the score of *PBX1*, a MkP priming TF, and more importantly, a regulator of HSC self-renewal^38^, was lower in the severe COVID-19 group than in controls (Supplementary Fig. S4c, h). Collectively, MkP- and GP-priming TFs, including *ETS2, FLI1, SPI1*, and *GATA2* were activated in the HSC/MPP cells from COVID-19 patients, probably contributing to the increased output of myeloid progenitor cells and decreased output of lymphoid progenitors.

### Depletion of lymphoid progenitors did not have significant impact on the production of SARS-CoV-2-specific antibodies or T-cell response in COVID-19 patients

To dissect the impact of the perturbed BM immune landscape on peripheral immune response during COVID-19, we analyzed the single-cell transcriptomic profile of paired PBMCs. This dataset included five paired PBMCs (M1, M3, S1, S2, and S3) collected 1 day before BM puncture, one from M2 collected 10 days before BM puncture, and 3 PBMCs from HCs^21^ as controls.

**Fig. 5:**
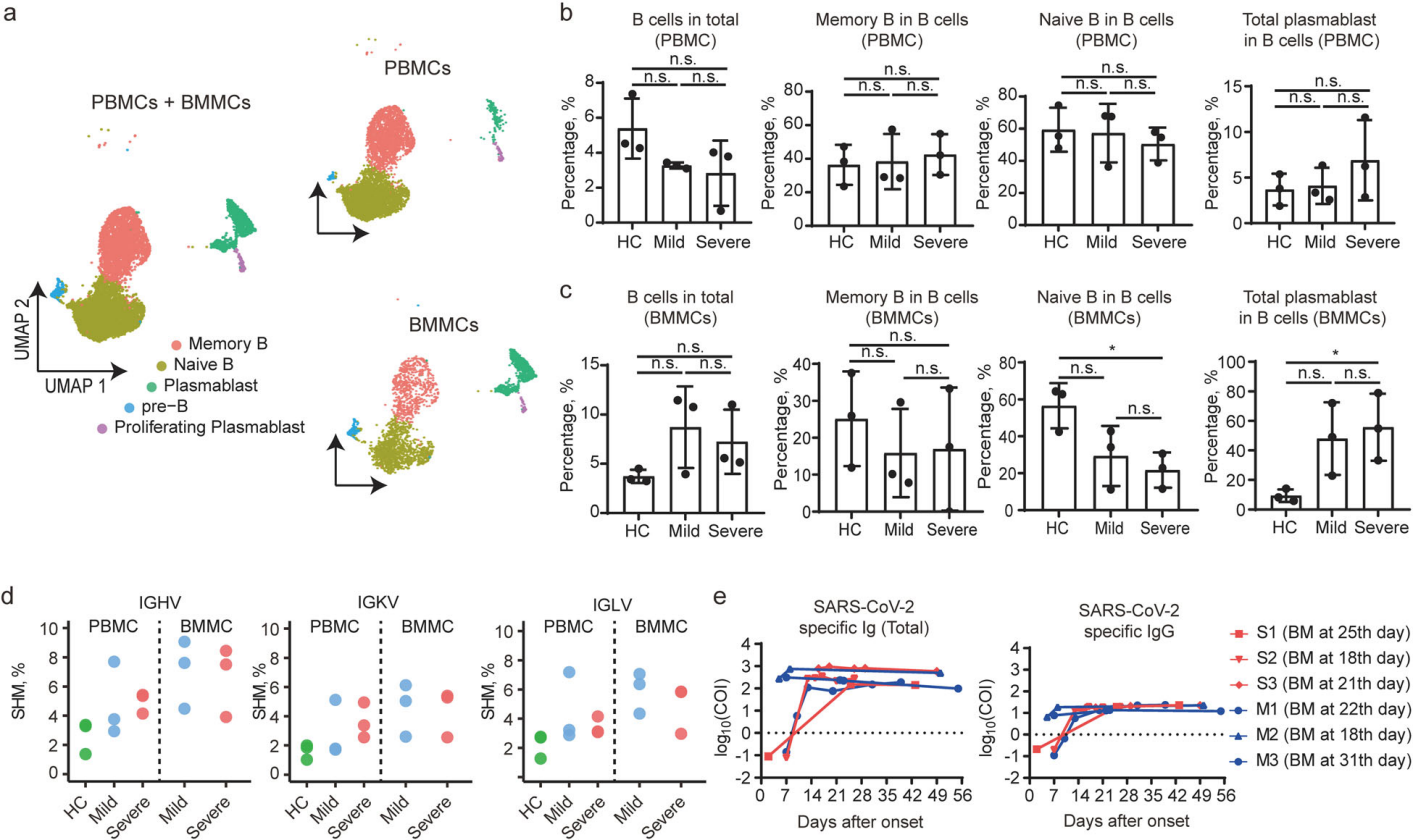
Analysis of B cells from BMMCs and paired PBMCs and production of SARS-CoV-2-specific antibody in COVID-19 patients. **a** UMAP plot of B cells from BMMCs and PBMCs. **b**, **c** Proportion of B cells among total cells and three types of B cells among total B cells, with results from PBMC samples (**b**) and BMMC samples (**c**). **d** Somatic hypermutation rate of IGHV, IGKV, and IGLV from three groups and two origins. **e** Total SARS-CoV-2-specific antibody titers and SARS-CoV-2-specific IgG titers of six patients at different time points after symptom onset. The BM puncture was done between the 18th and 31st day after symptom onset.

Combined analysis of BMMCs and PBMCs revealed that the B cells were comprised of five clusters, namely pre-B (characterized by *VPREB1, IGLL1*), naïve B cell (*IGHD, TCL1A*), memory B cell (*CD79B, CD82*), plasmablast (*IGHA1, IGHG1*), and proliferating plasmablast (*TUBA1B, GAPDH*) (Fig. 5a and Supplementary Fig. S5a). The proportion of B cells in PBMC cells decreased in COVID-19 patients when compared with controls, but it was not statistically significant. Additionally, no significant difference could be found in the proportions of memory B cells, naïve B cells, and total plasmablasts in different groups (Fig. 5b). Neither could it be found in the proportion of B cells in BMMC cells from COVID-19 patients (Fig. 5c). However, the increased accumulation of antibody-secreting cells in BM could be indicated by significant decrease in naïve B cells along with considerable increase in plasma cells identified in patients with severe COVID-19 when compared with mild cases (Fig. 5c). The amount of somatic hypermutations (SHM) of the B-cell receptors (BCRs) in PBMCs from COVID-19 patients was also found to be higher than controls (Fig. 5d).

We then tested the serial serum samples collected between 0 and 56 days after hospitalization to figure out whether the decrease of lymphoid progenitor cells affected the production of SARS-CoV-2-specific antibodies among the six studied COVID-19 patients. The SARS-CoV-2-specific antibodies detected in mild and severe cases were at similar levels, including SARS-CoV-2-specific total immunoglobulins (Fig. 5e, left panel), IgGs (Fig. 5e, right panel), IgA (Supplementary Fig. S5b, left panel), and IgM (Supplementary Fig. S5b, right panel).

With further analysis of the NK & T cell compartments from PBMCs and BMMCs, we identified 14 clusters, namely *CD4*^+^*COTL1*^high^ T, *CD4*^+^*GZMH*^high^ T, *CD4*^+^*GZMK*^high^ T, *CD4*^+^*LTB*^high^ T, *CD4*^+^ naïve T, *CD8*^+^*GZMA*^high^ T, *CD8*^+^*GZMH*^high^ T, *CD8*^+^*GZMK*^high^ T, *CD8*^+^ naïve T, γδ T, NK, proliferating cells, MAIT, and Treg (Supplementary Fig. S5c–f). There was lower proportion of *CD4*^+^*GZMK*^high^ T cells (Supplementary Fig. S6a), but a higher proportion of *CD4*^+^*GZMH*^high^ T cells in severe cases than in mild cases (Supplementary Fig. S6a). T-cell receptor (TCR) clonotype analysis revealed that clonally expanded T cells in the PBMCs were mainly comprised of *CD4*^+^*GZMH*^high^ T cells, *CD8*^+^*GZMH*^high^ T cells, and *CD8*^+^*GZMK*^high^ T cells (Supplementary Fig. S6b), with T cell expansion level being significantly lower in BMMCs than in PBMCs (Supplementary Fig. S6c). Shannon diversity index analysis demonstrated lower TCR diversity of *CD4*^+^ T cells in severe cases, indicating a higher clonal expansion (Supplementary Fig. S6d) and higher TCR diversity of the *CD8*^+^ T cells in BMMCs from severe cases (Supplementary Fig. S6d). Migration analysis showed that the *CD4*^+^*GZMH*^high^ T, *CD4*^+^*GZMK*^high^ T, *CD8*^+^*GZMA*^high^ T, *CD8*^+^*GZMH*^high^ T, and *CD8*^+^*GZMK*^high^ T cells had higher levels of TCR sharing between PBMCs and BMMCs (Supplementary Fig. S6e, f), indicating significant migration of those subsets between the blood and BM niche. Further analysis showed that the clonally expanded T cells (>100 cells) were not hyperactivated or more exhausted (Supplementary Fig. S6g–j), suggesting preserved function of T cells from COVID-19 patients despite reduced lymphoid progenitors. Therefore, the depletion of BM lymphoid progenitors in COVID-19 patients did not have significant impact on the functional immune responses of their peripheral lymphoid compartments against the virus.

### Immature GMPs accumulated in severe COVID-19 patients

**Fig. 6:**
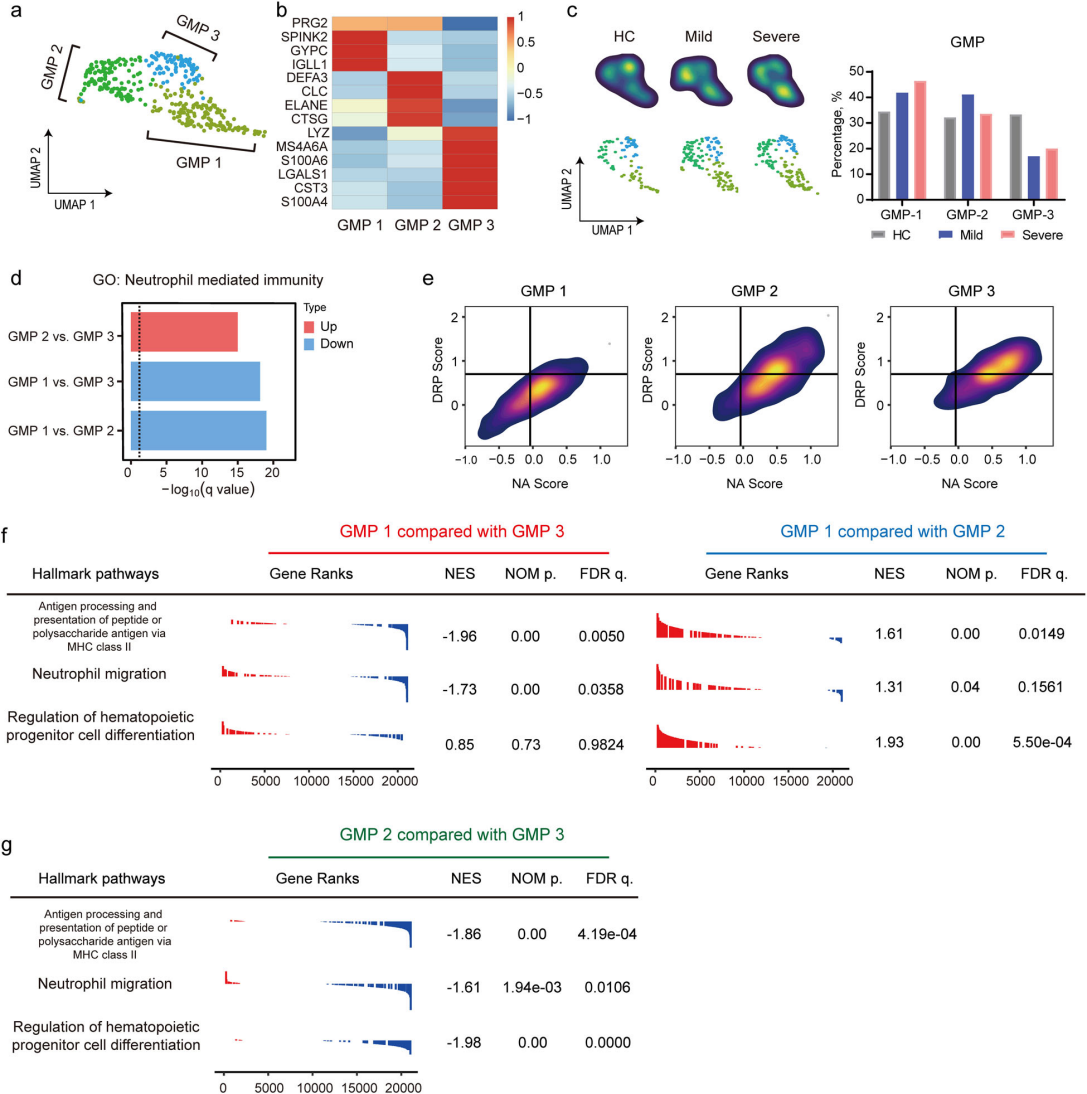
Immature and dysfunctional-like GMP was accumulated in severe COVID-19 patients. **a** UMAP plot of three clusters of GMP, namely GMP 1, GMP 2, and GMP 3. **b** Differential expression genes of three GMP clusters. **c** Density plot of three GMP clusters in three groups. **d** Up-regulation or down-regulation of GO pathway “Neutrophil-mediated immunity” in three clusters of GMP. **e** DRP score and NA score of the three GMP types. **f**, **g** GSEA analysis of GMP 1, GMP 2, and GMP 3 on the three hallmark pathways.

As reported recently, accumulation of immature and immunosuppressive neutrophil was found in both the blood and lungs of COVID-19 patients^14,15^, suggesting emergency myelopoiesis. As revealed above, we identified increase in GMPs from patients with severe COVID-19 (Fig. 2j), and therefore decided to extract the GMPs cluster for further analysis. Three GMP clusters were then identified (Fig. 6a) as GMP-1 (*SPINK2, GYPC*), GMP-2 (*ELANE, DEFA3*), and GMP-3 (*LYZ, LGALS1, S100A6, S100A4*) (Fig. 6b). GMP-3, GMP-2, and GMP-1 were enriched in HC, mild, and severe cases, respectively (Fig. 6c). Pre-maturation markers, such as *MPO*, were enriched in GMPs (Supplementary Fig. S7a), which are reminiscent of immature monocytes/neutrophils^39^ that produced under pathological conditions, including severe infection and sepsis^15^. Compared with GMP-1 in the HC group, GMP-1 from the patient group contained more *S100A8*^high^ (Supplementary Fig. S7b) and *HLA-DRA*^low^ (Supplementary Fig. S7d) cells and also highly expressed IFN-stimulated genes, such as *ISG15* (Supplementary Fig. S7b). In addition, previously reported genes (*CD24*, *LCN2*. Supplementary Fig. S7c), which were associated with poor clinical outcomes in sepsis, together with functional exhaustion makers (*CD274, ARG1*, Supplementary Fig. S7e) were not well expressed^15,40^. GMP-2 had a similar transcriptional profile with GMP-1 (Supplementary Fig. S7a–e), while GMP-3 had lower levels of *ISG15* (Supplementary Fig. S7b) and normal levels of *HLA-DR* related genes (Supplementary Fig. S7d).

Further analysis revealed that GMP-1 is likely to become dysfunctional in patients with severe COVID-19 (Fig. 6d, e). GO analysis showed the “neutrophil-mediated immunity” pathway was down-regulated in GMP-1, but up-regulated in GMP-2 (Fig. 6d). We also compared the signature scores of “defense response to pathogen (DRP)” and “neutrophil activation (NA)” (see Materials and methods section) among three sub-clusters of GMPs. Although the GMPs from patients had relatively higher DRP and NA scores than those from the HC (Supplementary Fig. S7f–h), GMP-1 had a significantly lower DRP and NA score than GMP-2 and GMP-3 (Fig. 6e). Gene Set Enrichment Analysis (GSEA) analysis revealed that the hallmark genesets associated with “antigen processing and presentation” and “migration” were down-regulated in GMP-1 compared with GMP-3 (Fig. 6f, g). In addition, analysis of pathways associated with “hematopoietic progenitor cell differentiation” indicated an upstream position of GMP-1 and GMP-3 (Fig. 6f, g). Immature GMP-1 was significantly accumulated in the BM of patients with severe COVID-19 and slightly increased in mild cases compared with controls, supporting the hypothesis that “emergency myelopoiesis” in severe COVID-19 leads to an accumulation of immature myeloid cells in the periphery and contributes to the pathophysiology of the disease.

## Discussion

Host immune responses play crucial roles in resisting viral infections, and a damaged immune response can induce a range of detrimental pathologic consequences^41^. Accumulating evidence has shown that patients with severe COVID-19 would exhibit cytokine storm syndrome^2,18^, which was often associated with an imbalanced immune system, and were characterized by the accumulation of myeloid cells and depletion of lymphoid cells^8,21,23^. Moreover, it is unclear whether lymphopenia is caused directly by SARS-CoV-2 infection, or via a cytokine storm in an indirect manner^5^. This study has provided novel evidence connecting lymphopenia with suppressed generation of lymphoid progenitors from HSC/MPP cells in COVID-19 patients. This suppression was associated with specific up-regulation of granulocyte-monocyte and megakaryocyte progenitor-priming TFs (including *SPI1, LMO4, ETS2, FLI1*, and *GATA2*) in HSC/MPP, thus leading to an increased production of GMP, MkP, and EP from HSC/MPP, accompanied by the reduction of lymphoid progenitors. Along with the poor ability to regulate hematopoietic progenitors, increased proportions of myeloid cells, monocytes, and granulocytes, as well as a depletion of T cells in BMMCs from COVID-19 patients were observed in this study, and increase in the proportion of immature granulocytes in the blood of these COVID-19 patients was also identified, particularly in those severe cases (Table 1).

Up-regulation of *ETS2, FLI1, GATA2*, and *ETV6* in the HSC/MPPs of COVID-19 patients raised the speculation that the HSC/MPPs preferentially differentiated into MkP cells. However, the percentage of MEP cells and upstream of MkP from COVID-19 patients were reduced in comparison to controls. These counterintuitive results may be attributed to several factors. First, HSC/MPPs in COVID-19 patients could be more prone to necroptosis and apoptosis than those in controls, which might lead to the loss of progenitor cells. Second, the growing evidence shows that HSCs are megakaryocyte-primed by default^27,42^, and thus they may bypass the traditional MEP differentiation checkpoint. Consistently, as the marker of megakaryocyte-biased HSCs, the expression of *ITGA2B* (CD41) was up-regulated in the HSC/MPPs of COVID-19 patients, despite the rarely found *VWF*^+^ HSC/MPP (a megakaryocyte-primed HSC)^42^. Third, the MEP cells might preferentially differentiate into other downstream subsets, e.g. the EP, thus resulting in a significant increase of EP and a decrease of MEP. These can be questions that require further investigation in the future.

With the potential to lead to high level of cytokines and stimulate the HSC/MPP cells to cell death, the increased expression of the receptors of coronavirus and influenza observed in this study suggested that correlation may exist between the enhanced differentiation of HSC/MPPs into MkP and GMP in COVID-19 patients and acute infection by SARS-CoV-2, or other co-infected pathogens. Indeed, evidence shows that HSPCs can be activated by viral infection, IFNs, and cytokines. Previously, stem-like Mk-committed progenitors (SL-MkPs) were identified within the HSC compartment^43^. In response to stress, acute inflammatory signaling triggered cell cycle activation of quiescent SL-MkPs, thereby driving a rapid maturation program of SL-MkPs and other MkPs, promoting platelet recovery after inflammation-induced thrombocytopenia.

It should be noted that there are some limitations of this study. Firstly, only three mild and three severe BMMC samples were enrolled in this study, which limited the number of hematopoietic stem cells and progenitor cells in silico, ex vivo, and in vitro analyses. Secondly, the whole blood should be used when investigating the differentiation from immature/dysfunctional-like GMPs to immature/dysfunctional neutrophils. The PBMC samples used in this study were consistent with the previous reports, but did not include neutrophils as these cells were excluded through Ficoll density centrifugation. Moreover, the BMMC samples from other acute respiratory virus-related diseases, such as influenza, should be enrolled as controls, to further identify specific features of COVID-19.

In conclusion, by identifying the drastic decrease in the number of lymphoid progenitors along with significant accumulation of immature and dysfunctional-like GMPs in BM of patients with severe COVID-19 as the possible cause of the biased differentiation of HSC/MPP cells into granulocyte–monocytes rather than lymphoid lineage, this study highlights the potential of therapeutics that correct the imbalanced differentiation of HSC/MPPs to be adopted as clinical treatment of COVID-19.

## Materials and methods

### Preparation of BMMCs and PBMCs

We collected BM aspirates and blood samples from three patients with mild COVID-19 (M1, M2, M3) and three with severe COVID-19 (S1, S2, S3), and used Public BMMCs scRNA-seq data of three age-matched healthy subjects^29^ and three PBMCs from HCs^21^ as controls. The study protocols have been approved by the Institutional Ethics Committee of Shenzhen Third People’s Hospital (Approval code 2020-208), and written informed consent was obtained from each enrolled subject. Patients’ blood test results are provided in Table 1. Red-blood-cell lysis buffer was used to treat BMMCs and PBMCs freshly isolated from the BM aspirates and blood samples by Ficoll density gradient separation.

### ScRNA-seq library construction and sequencing

Barcoded scRNA-seq libraries were prepared using the Chromium Single Cell V(D)J Reagent kit (10x Genomics, PN-1000006) according to the manufacturer’s instructions. A suspension of ~2 × 10^4^ purified BMMCs or PBMCs was used to form Gel Beads-in-emulsion for each sample. Subsequent sequencing was performed on the Illumina NovaSeq6000 platform. The raw sequencing reads of scRNA-seq and scTCR/scBCR-seq of BMMCs and PBMCs generated for this study have been stored in the National Genomics Data Center (https://bigd.big.ac.cn/), accession number: HRA000233 (BMMCs), HRA000555 (BMMCs BCR & TCR), HRA000517 (PBMCs), and HRA000550 (PBMCs BCR & TCR). In addition, three BMMCs scRNA-seq datasets (GSM3396162, GSM3396167, and GSM3396169) from age-matched healthy people^29^ were utilized as controls.

### Single-cell TCR and BCR analysis

The amino acid and nucleotide sequence of TCR chains were assembled and annotated by CellRanger vdj program (version 3.1.0). Only cells with paired TRA and TRB were used for clonality analysis. An exact match of nucleotide sequences of the CDR3 of both TRA and TRB were used to define the same TCR clonotype. Statistical analysis of Shannon index used to measure the cross-compartment clonal diversity were performed using *t*-tests, and TCR migration between PBMC and BMMC was delineated using R package STARTRAC (version 0.1.0).

The nucleotide sequence of BCR chains was assembled by CellRanger vdj program (version 3.1.0), and annotated by SCIGA (https://github.com/sciensic/SCIGA). Matched V, J gene and CDR3 nucleotide sequences were used to define the same BCR clonotype. Statistical comparisons of Shannon index used to measure the cross-compartment clonal diversity were performed using *t*-test.

### Dimension reduction and clustering analysis

The read count for each gene in each cell was quantified by the Cell Ranger pipeline (version 3.1, GRCh38 genome assembly) with default settings, then filtered gene-barcode matrices were analyzed with R Seurat package (version 3.1.5)^44^. Genes detected in <3 cells, and cells where mitochondrial gene accounts for >10% of the total, and with fewer than 200 or >4500 detected genes were removed. The raw unique molecule identifiers (UMIs) of genes were normalized using the *“NormalizeData”* function with default parameters. Next, the *“IntegrateData”* function was applied to correct batch effect in COVID-19 patients and HC. The *“RunPCA”* function was performed based on the top 2000 features generated by the *“FindVariableFeatures”* function and then UMAP of single cells was performed by the *“RunUMAP”* function. Finally, we adopted *“FindNeighbors”* and *“FindClusters”* to identify cell clusters at resolution of 0.8 and visualized them by UMAP with default settings. HSPCs and myeloid cells were re-clustered following the similar steps described above, including the removal of contaminated cells, dimension reduction, and clustering analysis.

### Identification of marker genes and annotation of cell clusters

Marker genes for each cluster were identified with the MAST algorithm in the *FindAllMarkers* function^45^ of Seurat. The filtering criteria for marker genes was |lnFC|> 0.25, p.adjust < 0.05, and pct.1>0.25. The cell clusters were annotated by referring to previously reported cell-type-specific marker genes^46^, and by retrieving cell type information from a curated immunophenotype-based reference dataset using the *“TransferData”* function in Seurat.

### Cell cycle score and status

Cell cycle scores and the status of HSC/MPP cells were calculated by the *“cyclone”* function in the scran (version 1.12.1) package^47^.

### RNA velocity

The alignment of reads with annotation of exons and introns, which was generated by the Cell Ranger pipeline, was processed using velocyto (version 0.17) “run10x” command and velocyto loom files were obtained. The Seurat Wrappers package Velocity (version 0.6) was then used to estimate RNA velocity following gene filtering process (2000 genes with the highest variance and a minimum feature counts of three detected in three cells). Spliced and unspliced counts were normalized separately based on total counts per cell, PCA, clustering, and UMAP plotting using the default setting. Gamma fitting, RNA velocity calculations, and Markov process simulation were also conducted.

### Calculating gene expression signature scores

The cell gene expression signature score was calculated using the “*AddModuleScore*” function in Seurat. The stemness score of the HSPC cell was calculated with a geneset that was comprised of overlapped differential expression genes from reference HSC/MPPs^28^ and our HSC/MPP: *ADAM28, AIF1, ANGPT1, ANKRD28, AREG, AVP, BEX2, BEX4, BST2, CD164, CD44, CRHBP, CRYGD, CSF3R, CYTL1, ELMO1, FOS, FXYD5, H1F0, HEMGN, HOPX, IDS, LAPTM4A, LIMS1, LST1, MDK, MLLT3, MSRB3, MYCT1, NPR3, RBPMS, SELL, SERPINB1, SMIM24, SPINK2, TAOK3, TFPI*, and *ZFP36*. The activity signature scores of the T cells from BMMC and PBMC were calculated using the following genes: *CD69, CCL19, CCL2, CCL21, CCL5, CCR2, CCR7, CD24, CD27, CD276, CD28, CD40LG, CD46, CD47, CD5, CD55, CD6, CD70, CD74, CD80, CD81, CD83, CD86, CDC42, GATA3, HMGB1, ICOS, IFNG, IL12A, IL15, IL18, IL1A, IL1B, IL1RL2, IL2, IL21, IL23A, IL23R, IL27RA, IL2RA, IL36B, IL4, IL4R, IL7, IL7R, LGALS9, TNFRSF14, TNFSF11*, and *TNFSF13B*. The exhaustion scores of the T cells from BMMC and PBMC were calculated using the following genes: *PDCD1, LAG3, TIGIT, HAVCR2, TOX, CD160, CTLA4*, and *EOMES. LYZ, CST3, RETN, FCER1G, TYROBP, S100A8, ANXA2, FTL, GRN, S100A9, RAB31, PRTN3, STXBP2, MNDA, CTSZ, CTSG, MPO, AZU1, PLAC8, CFD, TUBB4B, S100A11, PRDX4*, and *ASAH1* were used to calculate the “defend response to pathogen score (DRP)” of GMP cells, and *LYZ, FCER1G, S100A8, RNASE6, GRN, S100A9, IRF8, CTSG, MPO, AZU1, PLAC8, ISG15*, and *HMGB2* were used to calculate the “neutrophil activation (NA)” score.

### Reads mapping and tracking the SARS-CoV-2 genome

We adopted two methods to identify the infection of BMMCs by SARS-CoV-2. One was to align BMMCs scRNA-seq dataset to the SARS-CoV-2 genome (NC_045512.2) using the Cell Ranger pipeline, with a previously reported bronchoalveolar lavage fluid (BALF) cell scRNA-seq dataset (GSE145926)^6^ that was tested positive for SARS-CoV-2 being included as the positive control. The other was to use Viral-Track^31^ to detect viral reads from the scRNA-seq dataset using the default parameter setting.

### Flow cytometry staining of the HSPCs

Purified BMMCs of the six COVID-19 patients were used to stain the HSPCs and flow cytometry was performed on FACSymphony^TM^ S6 (BD Biosciences). The following antibodies for the HSPCs were used: lineage cocktail (CD3, CD14, CD16, CD19, CD20, CD56/Pacific Blue) (Biolegend, clone UCHT1; HCD14, 3G8, HIB19, 2H7, HCD56, Cat. no. 348805, RRID: AB_2889063), CD34/APC (BD Biosciences, clone 581, Cat. no. 555824, RRID: AB_398614), CD38/PE-Cy7 (Biolegend, clone HIT2, Cat. no. 303516, RRID: AB_2072782), CD90/PerCP-Cy5.5 (BD Biosciences, clone 5E10, Cat. no. 561557, RRID: AB_10712762), CD45RA/APC-Cy7 (Biolegend, clone HI100, Cat. no. 304128, RRID: AB_10708880), CD49f/BV605 (BD Biosciences, clone GoH3, Cat. no. 740416, RRID: AB_2740146), CD10/BV786 (BD Biosciences, clone HI10a, Cat. no. 564960, RRID: AB_2739025), LIVE/DEAD dye/BV510 (Invitrogen, Cat. no. L34957), and Annexin V/FITC (BD Biosciences, Cat. no. 556547, RRID: AB_2869082). Gatings included HSC (Lin^−^CD34^+^CD38^−^CD45RA^−^CD90^+^CD49f^+^CD10^−^), MPP (Lin^−^CD34^+^CD38^−^CD45RA^−^CD90^−^CD10^−^).

### Functional annotation analysis

The *“FindMarkers”* was applied to detect the DEGs between any two given groups. GO and Kyoto Encyclopedia of Genes and Genomes (KEGG) pathway analysis of DEGs were performed with clusterProfiler R package^48^, and only terms in the “GO Biological Processes” were considered in the GO enrichment analysis. In addition, GSEA was also included and performed with C2 (curated), C3 (regulatory target), and C5 (Gene Ontology) in MSigDB.

### Stem-score based stratification analysis

HSC/MPP cells were stratified by descending stemness signature scores^32^ (V, 95%–100% percentiles, IV, 90%–95% percentiles, III, 85%–90% percentiles, II, 80%–85% percentiles, and I, 75%–80% percentiles). Correlated expression (by Spearman correlation) of different lineage-specific gene modules was assessed in each stratified HSC/MPP population.

### Regulatory network inference

The single-cell regulatory network inference and clustering (SCENIC)^37^ was used to explore the regulatory landscape based on normalized expression levels of scRNA-seq data. Only genes expressed in at least 1% of HSC cells were retained to construct the regulatory network. The TF and directly regulated genes were identified as regulons. In total, 149 regulons were identified with a threshold of at least 10 co-expressed genes per regulon. The Student’s *t* test (R 3.6.2, two-sided, unadjusted for multiple comparisons) was used to analyze differences of AUCell score in a pairwise fashion among the three groups (HC, mild, and severe). Differentially activated regulons with a *P* value < 0.05 in any of the pairwise comparisons were selected for further clustering. Finally, all regulons were clustered based on the AUCell score of the enriched TFs in each single cell using Pheatmap R package.

### Detection of SARS-CoV-2-specific immunoglobulin using Chemiluminescence Microparticle Immuno Assay

The SARS-CoV-2-specific immunoglobulin in plasma was tested with Chemiluminescence Microparticle Immuno Assay kit (Beijing Wantai Biological Pharmacy Enterprise Co., Ltd., China). The antibody concentration was represented by fluorescence intensity, which was presented as the relative fluorescence of the sample to control (COI), with COI > 1 indicating positive testing results for SARS-CoV-2-specific antibody.

### Statistics

The two-tailed unpaired Student’s *t* test (Prism version 8.0.1, GraphPad Software, San Diego, CA) was used to explore differences lying in the abundance of cell types for all cell clusters in different groups. *P* value < 0.05 was considered statistically significant.

### Study approval

All procedures were performed in accordance with the ethical standards of the responsible committee on human experimentation (institutional and national) and with the Helsinki Declaration of 1975, as revised in 2000. Informed consent was obtained from all patients prior to inclusion in the study.

## Supplementary information

Supplementary Information
