# Supplementary material for: Dysregulated hematopoiesis in bone marrow marks severe COVID-19
Source: Cell Discov. 2021 Aug 4;7:60. doi: 10.1038/s41421-021-00296-9 (PMC8335717; doi:10.1038/s41421-021-00296-9)
Supplement: Supplementary file 1 — Supplementary Information [file 41421_2021_296_MOESM1_ESM.pdf]

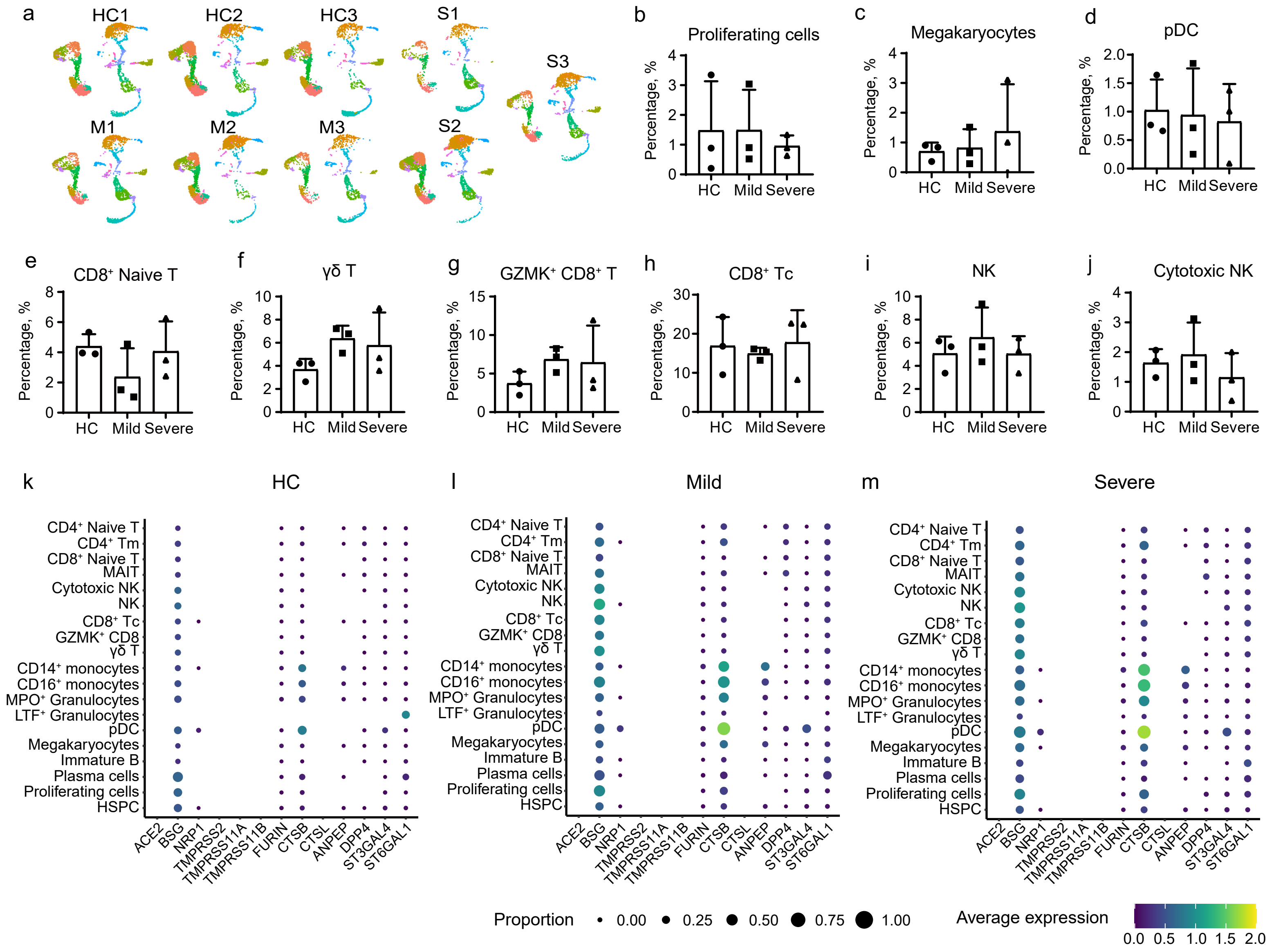

Supplementary Fig. S1 Characterization of BMMCs and susceptibility of BMMCs to SARS-CoV-2 and other viruses (related to Fig. 1). (a) Separated UMAP plots of BMMCs from each subject. (b-j) The proportions of nine clusters in BMMCs. (k-m) The transcription levels of *ACE2, BSG, NRP1, TMPRSS2, TMPRSS11A, TMPRSS11B, FURIN, CTSB, CTSL, ANPEP, DPP4, ST3GAL4,* and *ST6GAL1* in various cell types from healthy controls (k), mild COVID-19 patients (l), and severe COVID-19 patients (m).

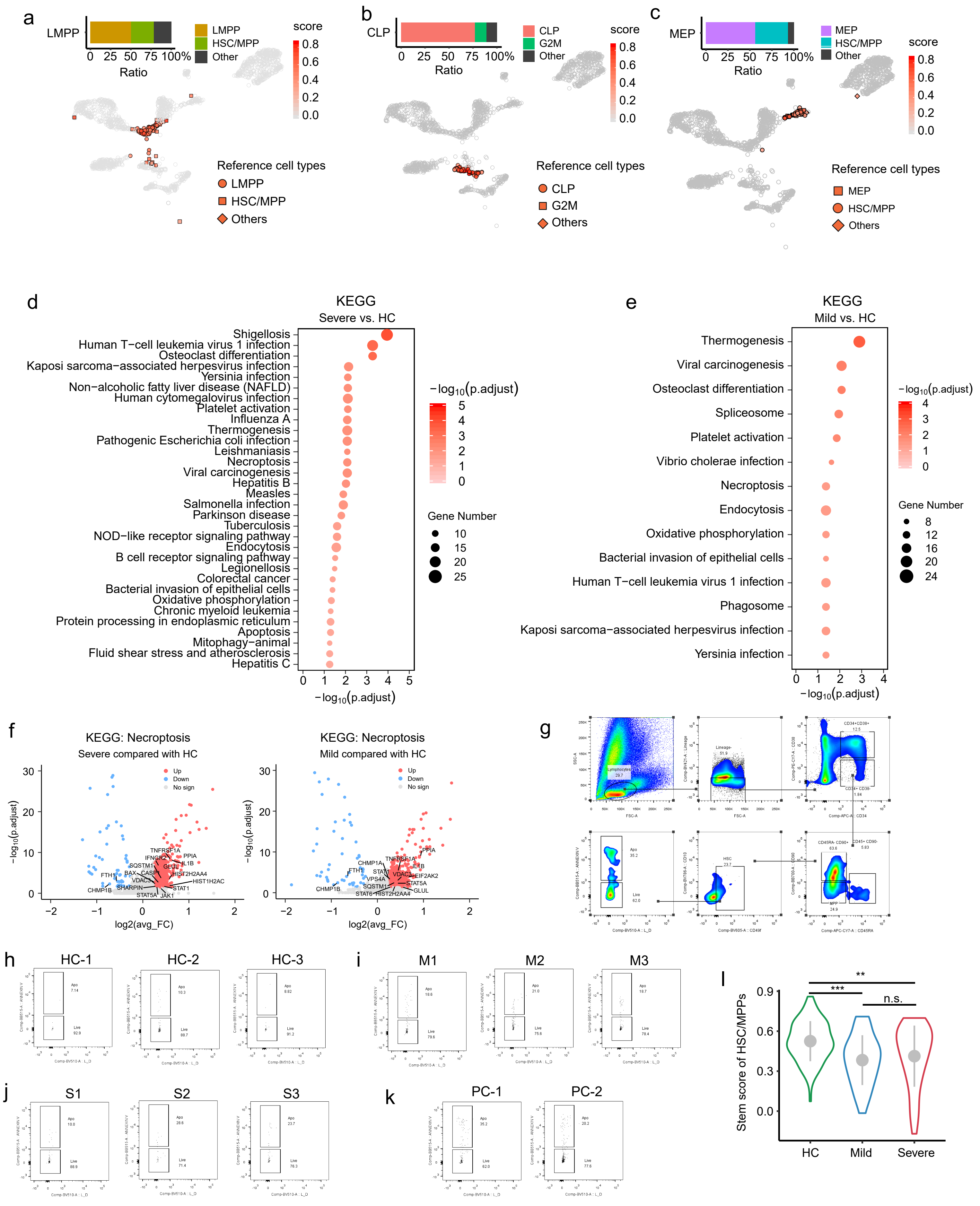

Supplementary Fig. S2 Clustering analysis of HSPC and DEG analysis of HSC/MPP (related to Fig. 2 and Fig. 3) (a-c) Label transfer of LMPP, CLP, and MEP population according to an immunophenotype based reference dataset. (d) KEGG analysis of DEGs in HSC/MPP cells between severe COVID-19 patients and healthy controls.(e) KEGG analysis of DEGs in HSC/MPP cells between mild COVID-19 patients and healthy controls. (f) The volcano plot shows the DEGs associated with the KEGG pathway “Necroptosis” (Entry ID: hsa04217) in HSC/MPP between COVID-19 patients and controls. (g) Gating strategy of flow cytometry analysis to identify HSC and Annexin V+ HSCs. (h-k) Dot plots of Annexin V+ HSC in each sample. (l) Stem score of HSC/MPPs in 3 groups.

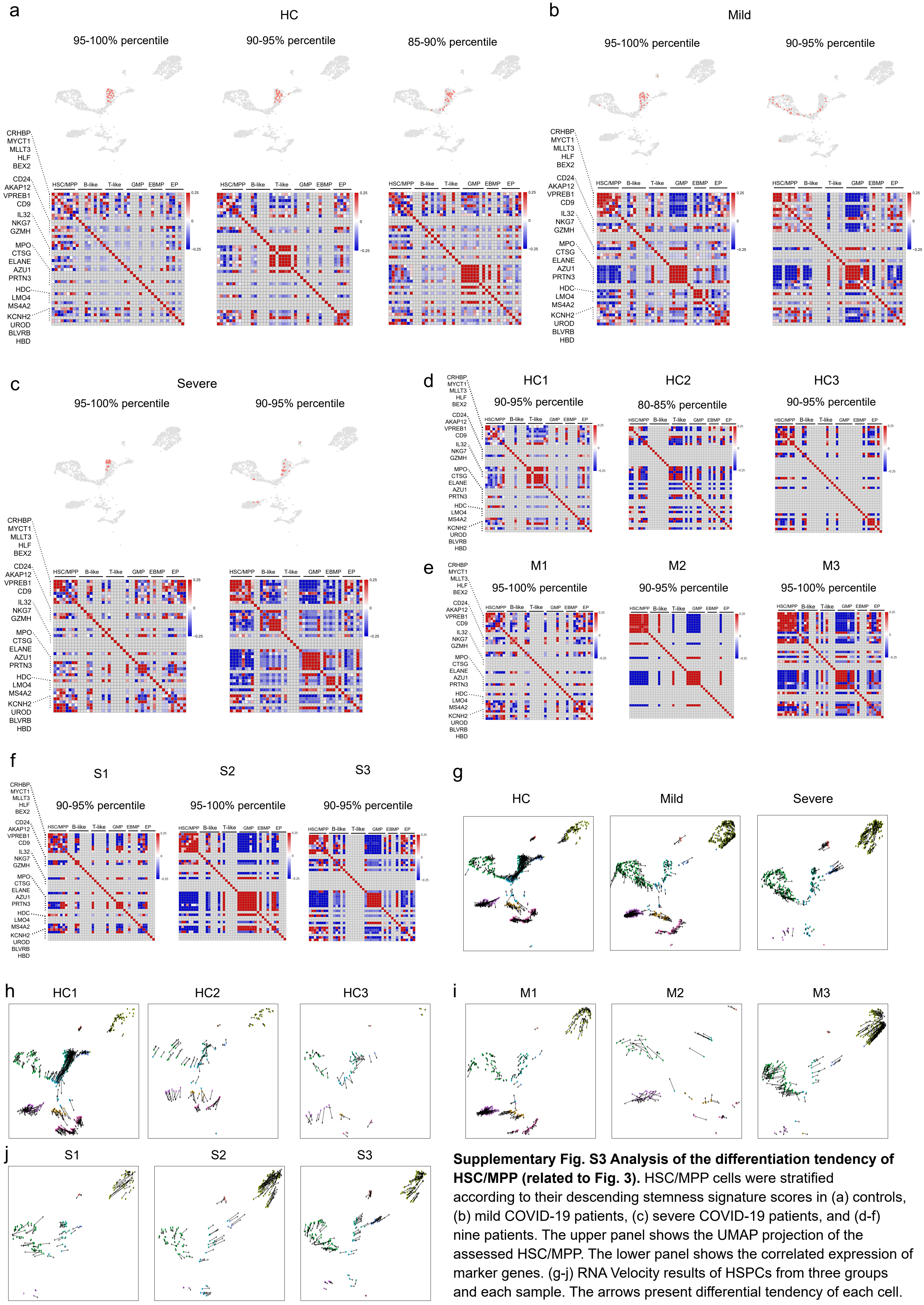

Supplementary Fig. S3 Analysis of the differentiation tendency of HSC/MPP (related to Fig. 3). HSC/MPP cells were stratified according to their descending stemness signature scores in (a) controls, (b) mild COVID-19 patients, (c) severe COVID-19 patients, and (d-f) nine patients. The upper panel shows the UMAP projection of the assessed HSC/MPP. The lower panel shows the correlated expression of marker genes. (g-j) RNA Velocity results of HSPCs from three groups and each sample. The arrows present differential tendency of each cell.

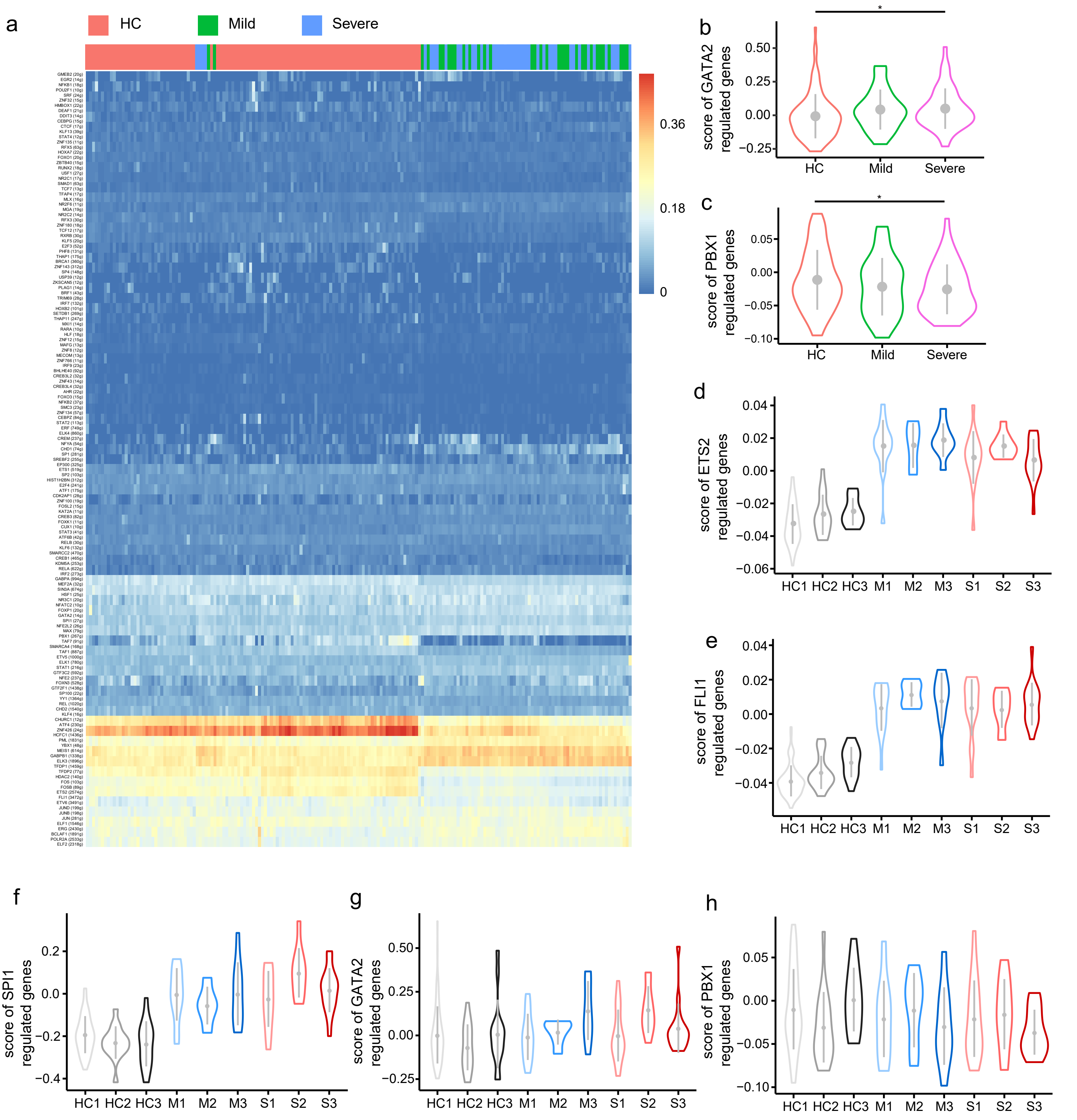

Supplementary Fig. S4 Transcriptional regulation and clustering analysis of myelopoiesis (related to Fig. 4). (a) The entire RASs matrix of HSC/MPP cells. (b-h) Score of specific TF regulated genes in the three groups or each sample.

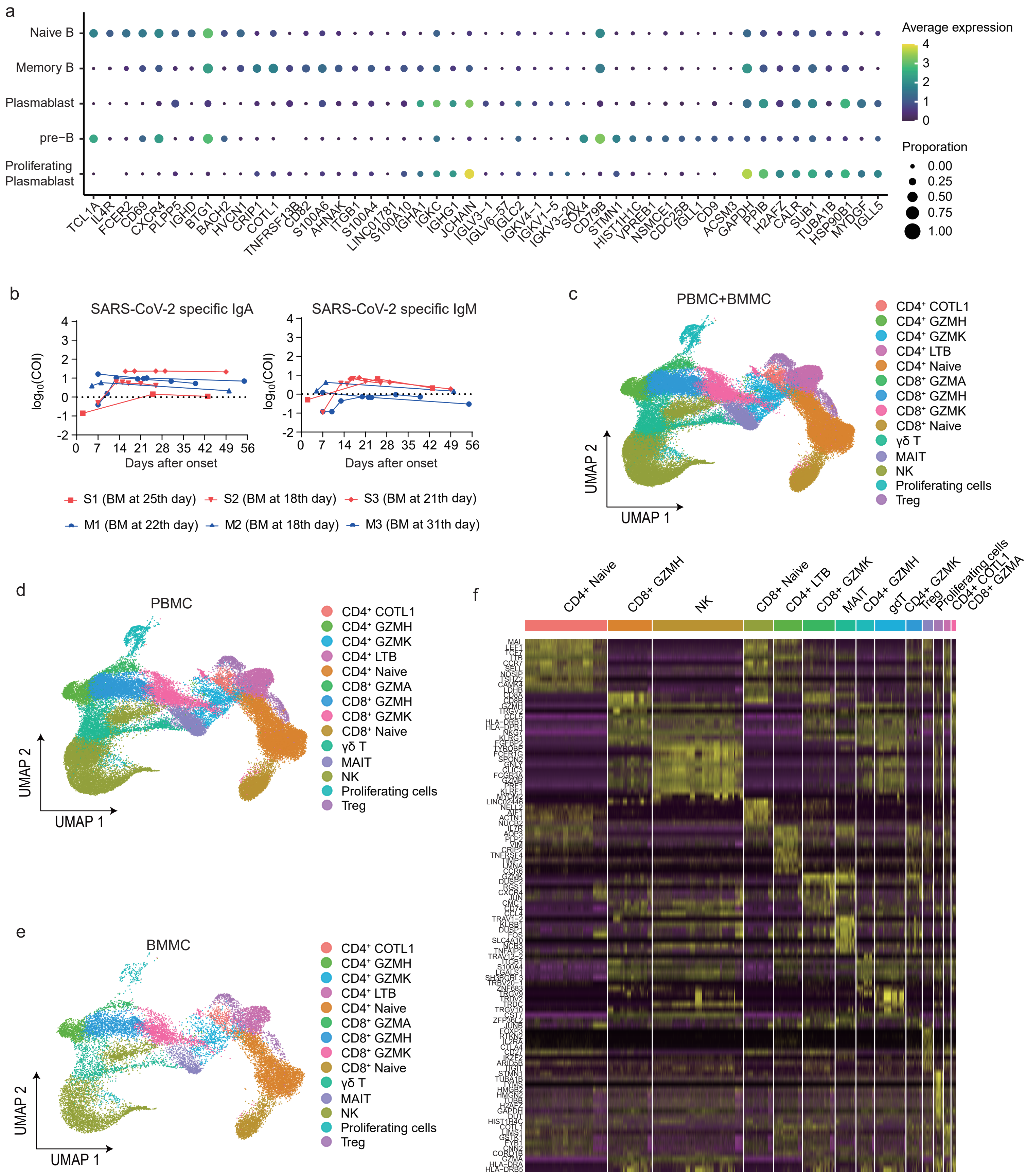

Supplementary Fig. S5 Characterization of lymphoid cells in PBMCs/BMMCs and production of SARSCoV-2 specific antibodies (related to Fig. 5). (a) Heatmap of differential expression genes of five B cell types. (b) SARS-CoV-2 specific IgA and IgM titers of six patients at different time points after symptom onset. The bone marrow puncture was done between the 11th and 28th day after hospitalization. (c-e) UMAP plot of T cells from BMMCs and PBMCs. (f) Heatmap of differential expression genes of fourteen NK & T cell types.

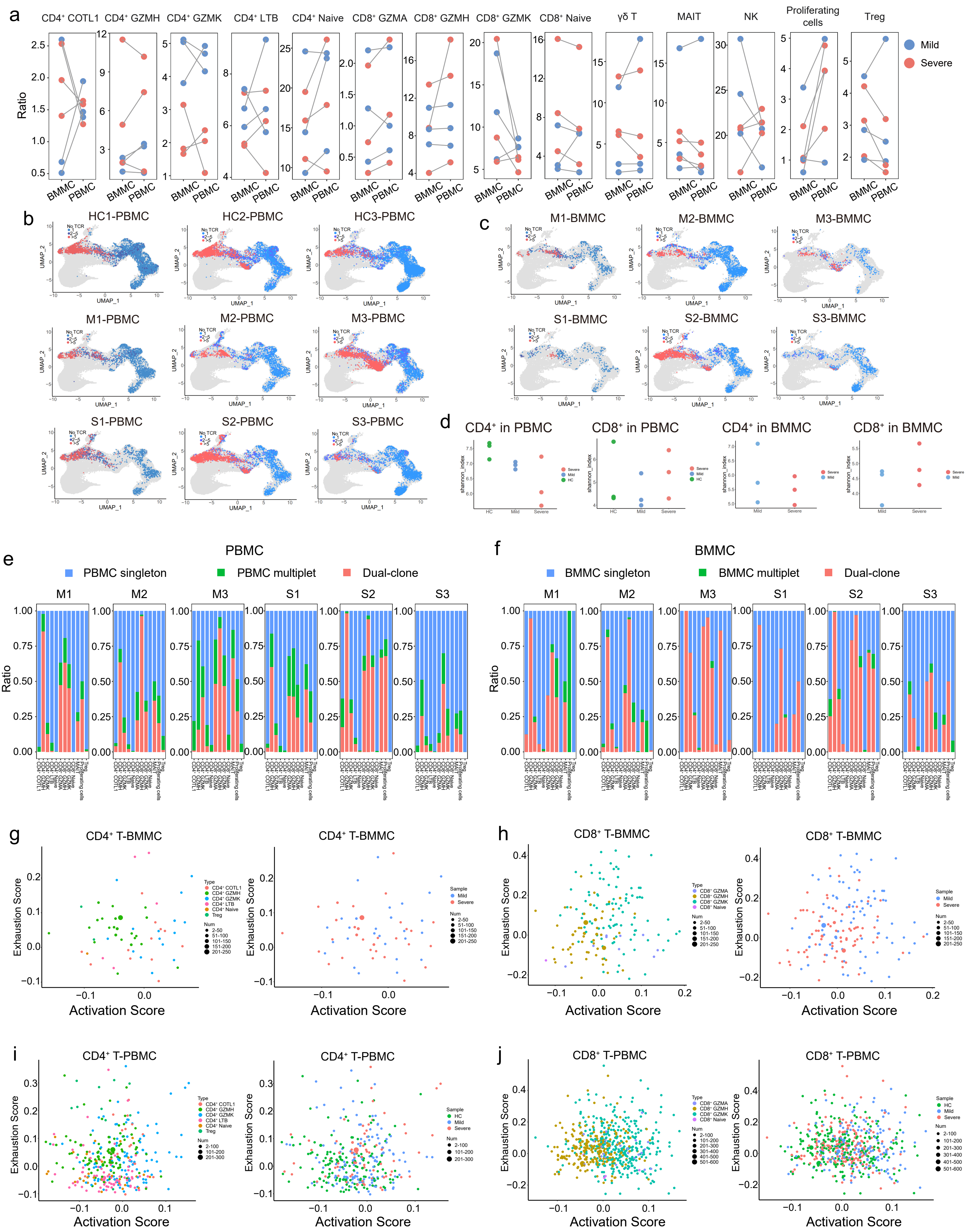

Supplementary Fig. S6 Characterization of T cells in PBMCs/BMMCs (related to Fig. 5). (a) Proportion of fourteen T cell types among total T cells in PBMCs and BMMCs. (b-c) Clonal expansion of TCRs in PBMCs/BMMCs of different samples. (d) Shannon diversity index of CD4⁺ T or CD8⁺ T in PBMCs/BMMCs of different groups. (e-f) Share of TCR clonotypes between BMMC and paired PBMC samples. Single represents non-expanded TCR clonotype, multi means expanded TCR clonotype, and dual indicates those clonotypes shared in paired PBMC and BMMC samples.(g-j) Exhaust score and active score of clonal expansion of CD4⁺ or CD8⁺ T cells in PBMC/BMMCs.

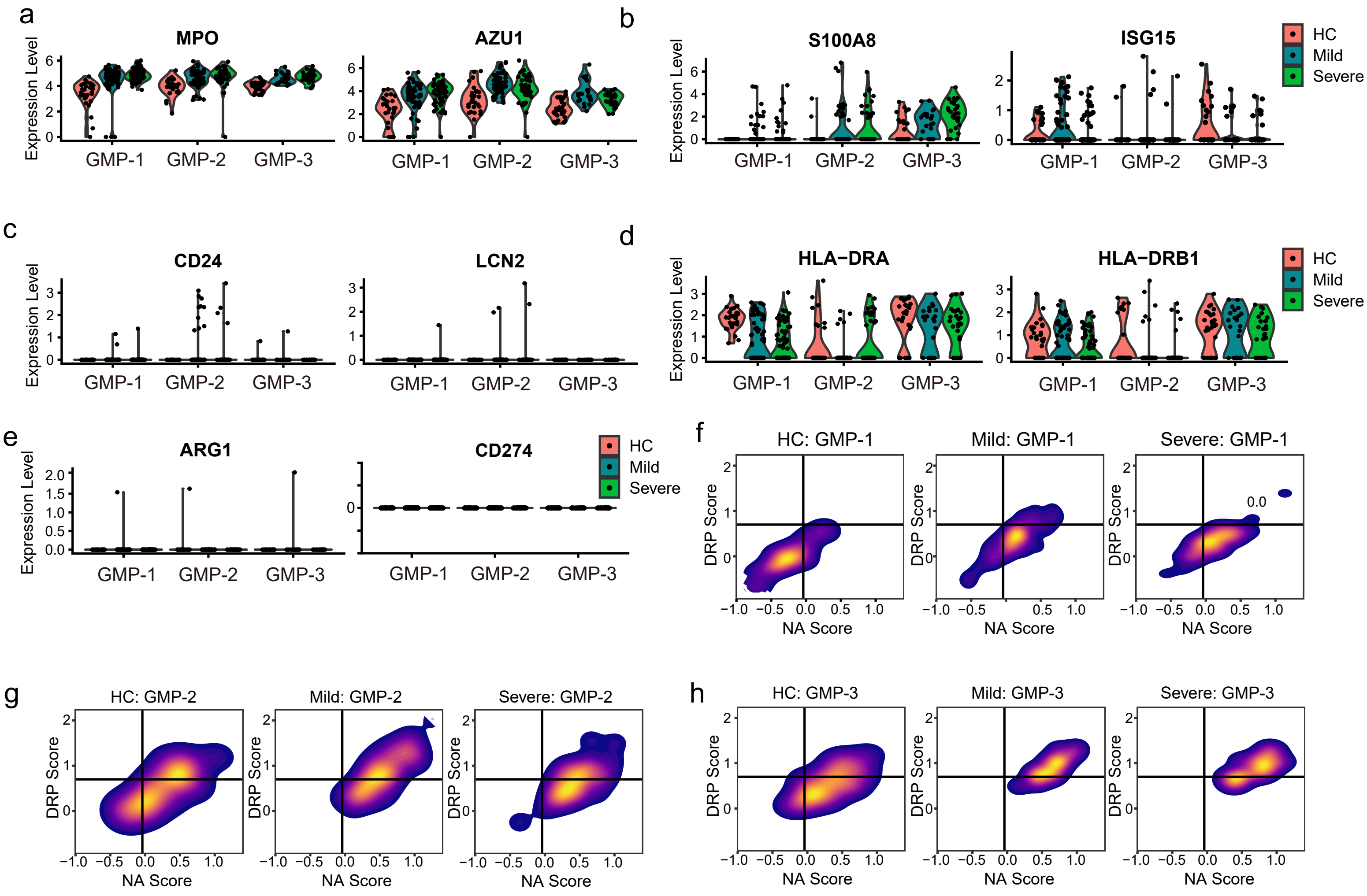

Supplementary Fig. S7 Characterization of GMPs in BMMCs (related to Fig. 6). (a-e) Violin plot of *MPO, AZU1, S100A8, ISG15, CD24, LCN2, HLA-DRA, HLA-DRB1, ARG1*, and *CD274* in GMP 1, GMP 2, and GMP 3 from the HC, mild, and severe group. (f-h) DRP scores and NA scores were calculated according three GMP types from the HC group, mild group, and severe group.

Supplementary Table S1. QC of scRNA

Sample ID	Estimated Number of Cells	Mean Reads per Cell	Median Genes per Cell
HC1	4,844	67,705	685
HC2	6,035	46,996	698
HC3	5,451	39,583	796
M1	4,817	173,154	1,581
M2	6,337	119,534	1,728
M3	4,243	181,168	808
S1	3,258	207,548	1,244
S2	8,765	100,820	1,672
S3	4,622	174,942	1,650
Average	5,375	123,494	1,207

Supplementary Table S2. DEG of HSC-MPP in patients and HCs (Part I: Severe vs HC), Related to Fig. 3 & Supplementary Fig. S2

gene	p_val	avg_logFC	pct.1	pct.2	p_val_adj
MT-ATP8	3.31E-46	3.264304852	0.977	0.037	6.90E-42
MT-ND4L	2.45E-59	2.669216959	0.977	0.626	5.09E-55
EEF1G	2.68E-57	2.650058428	1	0.252	5.58E-53
NME2	3.80E-47	2.251878146	1	0.131	7.90E-43
PTPRCAP	1.49E-36	1.547274748	0.955	0	3.10E-32
GABARAP	1.41E-30	1.516314257	1	0.159	2.95E-26
MT-ND6	5.34E-21	1.375902899	0.841	0.112	1.11E-16
ZFP36L2	1.01E-20	1.220741325	1	0.813	2.10E-16
MYC	1.33E-12	1.215725088	0.636	0.084	2.76E-08
ALDOA	1.55E-22	1.175620579	0.977	0.224	3.23E-18
RPL17	3.03E-35	1.125398849	1	0.981	6.31E-31
RNASEK	1.21E-26	1.049412776	0.864	0.019	2.52E-22
IFITM1	6.41E-20	1.02459544	0.818	0.065	1.33E-15
ATP6V0C	8.75E-27	1.01395233	0.841	0.009	1.82E-22
PCBP2	2.97E-24	0.941094742	0.977	0.832	6.18E-20
HLA-DQA1	1.14E-09	0.857173863	0.841	0.308	2.38E-05
WASHC1	2.39E-12	0.854645767	0.795	0.178	4.97E-08
CD74	8.74E-12	0.849821841	0.977	0.972	1.82E-07
MRPS24	4.37E-24	0.833966072	0.727	0	9.09E-20
PSMA6	5.94E-18	0.833814126	0.841	0.103	1.24E-13
TUBB	9.56E-11	0.831209136	0.977	0.57	1.99E-06
ITGA4	4.95E-14	0.813423839	0.773	0.131	1.03E-09
RCC1	1.33E-16	0.799264482	0.795	0.093	2.76E-12
TXNIP	4.42E-09	0.787299939	0.773	0.252	9.19E-05
MAT2A	8.75E-13	0.774409113	0.795	0.168	1.82E-08
PPIA	6.03E-14	0.773260688	1	0.879	1.26E-09
HIST1H1E	7.33E-12	0.763774631	0.523	0.028	1.53E-07
ACTG1	4.46E-14	0.751433096	1	0.981	9.28E-10
SERPINB1	5.88E-13	0.750680429	1	0.879	1.22E-08
MIF	2.93E-11	0.748568876	0.977	0.757	6.10E-07
PCBP1	8.48E-13	0.7409763	0.955	0.589	1.76E-08
NFE2	8.23E-17	0.739789571	0.864	0.14	1.71E-12
MT-CO2	3.08E-23	0.722356108	0.977	1	6.42E-19
TRAPPC5	1.96E-20	0.713612961	0.705	0.009	4.08E-16
IFI44L	4.59E-13	0.702376643	0.591	0.037	9.56E-09
ZYX	4.90E-12	0.675962091	0.886	0.271	1.02E-07
PGD	3.05E-11	0.672744657	0.841	0.243	6.36E-07
RHOA	3.38E-11	0.666562192	0.955	0.673	7.04E-07
ACTB	3.69E-10	0.66269025	1	0.953	7.68E-06
ETV6	1.61E-09	0.66162673	0.773	0.224	3.36E-05
POLD4	2.12E-16	0.658096716	0.659	0.028	4.40E-12
OXA1L	1.27E-10	0.656719327	0.864	0.299	2.64E-06
UBE2V1	1.13E-14	0.641707067	0.659	0.047	2.36E-10
TKT	2.38E-09	0.634036146	0.977	0.776	4.96E-05
SNHG7	1.19E-06	0.633794147	0.932	0.626	0.024783515
PFN1	5.34E-10	0.630072221	1	0.907	1.11E-05
TAGLN2	1.22E-07	0.625576907	0.977	0.72	0.002546015
MATR3.1	7.13E-17	0.625567269	0.614	0.009	1.48E-12
AKR1C3	6.86E-10	0.621331041	0.886	0.336	1.43E-05
IL1B	3.62E-13	0.612604461	0.705	0.093	7.53E-09
HMGN2	1.06E-06	0.600470471	1	0.757	0.02213196
TNFRSF1A	2.99E-13	0.600033632	0.864	0.215	6.22E-09

DDX39B	5.53E-14	0.598514403	0.659	0.056	1.15E-09
ARF5	5.65E-14	0.596576618	0.977	0.346	1.18E-09
HSPD1	1.68E-07	0.595334617	0.932	0.561	0.003503062
GUCY1B1	8.84E-12	0.591696934	0.614	0.065	1.84E-07
LRRC70	3.91E-16	0.585545638	0.523	0	8.13E-12
RAC2	2.15E-10	0.584456744	1	0.654	4.48E-06
CNST	2.17E-10	0.571601755	0.682	0.131	4.52E-06
IFI6	5.35E-09	0.568226566	0.682	0.168	0.000111441
HIST1H2AC	6.85E-07	0.562642443	0.545	0.121	0.014270198
INKA1	2.76E-14	0.560131668	0.705	0.075	5.76E-10
RAB37	1.24E-10	0.557231948	0.795	0.215	2.59E-06
HIST2H2AA4	1.50E-08	0.550580417	0.386	0.019	0.000311595
ZNF638	1.08E-12	0.549753599	0.773	0.15	2.24E-08
GATD3B	3.00E-15	0.547933873	0.568	0.009	6.24E-11
DDX17	3.86E-09	0.546213614	0.909	0.393	8.04E-05
MT-ATP6	1.93E-14	0.544047204	0.977	0.991	4.01E-10
CD34	4.74E-11	0.543170235	0.977	0.439	9.86E-07
PHB2	1.12E-06	0.540985531	0.886	0.514	0.023241519
GRINA	1.40E-11	0.539971578	0.682	0.112	2.92E-07
MRPL16	1.07E-10	0.539740528	0.909	0.336	2.22E-06
YWHAB	3.06E-07	0.533665491	0.932	0.561	0.006363374
RPLP0	1.44E-17	0.533293732	1	1	3.00E-13
MT-ND5	2.95E-09	0.532480927	0.977	0.981	6.14E-05
EMB	6.61E-08	0.52563392	0.568	0.112	0.001377156
LUC7L3	1.95E-07	0.524652907	0.864	0.393	0.004059988
DLK1	1.34E-09	0.523415707	0.318	0	2.78E-05
CAP1	1.92E-09	0.523035984	0.886	0.346	4.00E-05
SLC40A1	1.12E-09	0.52278492	0.75	0.206	2.33E-05
HIST1H1D	4.25E-14	0.517498997	0.614	0.037	8.86E-10
GPI	2.99E-10	0.514558232	0.705	0.15	6.23E-06
GLUL	5.01E-10	0.513199204	0.886	0.327	1.04E-05
MAT2B	7.06E-13	0.510466894	0.705	0.103	1.47E-08
PTGES3	1.04E-07	0.510297182	0.977	0.589	0.002156208
HNRNPA2B1	1.07E-06	0.507870348	0.977	0.757	0.022183591
PSAP	1.00E-07	0.507416911	0.818	0.327	0.002082465
CDK4	4.51E-10	0.507328739	0.864	0.299	9.40E-06
LY6E	1.65E-07	0.505741804	0.909	0.449	0.003428762
HCLS1	4.78E-13	0.505037566	0.841	0.206	9.95E-09
ATP6V0A2	8.02E-10	0.504315805	0.705	0.159	1.67E-05
APEX1	5.72E-07	0.504306404	0.977	0.645	0.011904349
WBP1	1.91E-19	0.503976649	0.614	0	3.99E-15
ZRANB2	7.34E-13	0.501938102	0.886	0.262	1.53E-08
GNG5	4.55E-07	0.500714041	0.932	0.542	0.009479895
DNAJA1	1.28E-08	0.500164947	0.864	0.346	0.000267342
SCAMP2	1.75E-11	0.498601617	0.773	0.178	3.64E-07
NRROS	1.81E-12	0.498489781	0.614	0.056	3.78E-08
SNRPA	8.25E-08	0.496166904	0.75	0.252	0.001717571
DDOST	8.77E-11	0.49553782	0.886	0.308	1.83E-06
GSTM2	4.54E-12	0.495042146	0.5	0.019	9.45E-08
MT-CO1	1.11E-12	0.493715603	0.977	1	2.31E-08
MBNL1	1.06E-07	0.491869809	0.886	0.411	0.002197712
IL18	1.03E-09	0.491126339	0.932	0.402	2.15E-05
GFI1B	6.11E-11	0.491013696	0.545	0.047	1.27E-06
MAPRE2	1.49E-06	0.490346907	0.705	0.252	0.031027582
ARPC1B	2.38E-07	0.490224742	0.955	0.617	0.004953234
KDELR1	1.10E-09	0.487339736	0.932	0.402	2.29E-05

LYL1	5.76E-07	0.486403863	0.795	0.327	0.011987754
KPNB1	3.65E-08	0.485306663	0.818	0.308	0.000760841
FERMT3	2.40E-11	0.482659005	0.705	0.131	5.00E-07
ARHGDIB	8.68E-09	0.480084023	1	0.953	0.000180798
SPTBN1	4.38E-07	0.478950123	0.682	0.215	0.009123283
DAZAP2	1.44E-07	0.472891241	0.864	0.383	0.002993172
PCBD1	4.02E-09	0.472608279	0.818	0.28	8.37E-05
BUB3	4.96E-11	0.469431749	0.727	0.159	1.03E-06
SENP6	3.15E-10	0.467493179	0.682	0.14	6.55E-06
DBNDD2	1.38E-14	0.467379866	0.477	0	2.88E-10
LCP2	2.31E-14	0.465978731	0.795	0.15	4.81E-10
CCDC88A	3.45E-09	0.465966463	0.75	0.215	7.19E-05
MT-CYB	2.56E-13	0.465895368	0.977	1	5.33E-09
XBP1	5.32E-07	0.465135793	0.932	0.505	0.011079502
CNPY3	4.86E-09	0.462236002	0.795	0.262	0.000101123
SLC9A3R1	1.68E-11	0.459307739	0.636	0.093	3.49E-07
M6PR	4.28E-11	0.459247954	0.795	0.215	8.90E-07
XRCC5	1.14E-11	0.459066648	0.932	0.346	2.37E-07
UCP2	2.96E-13	0.458499862	0.795	0.187	6.16E-09
NDUFV2	2.85E-10	0.457973442	0.886	0.327	5.93E-06
SPI1	6.59E-10	0.457946712	0.682	0.15	1.37E-05
KDM5B	8.23E-10	0.457083309	0.568	0.075	1.71E-05
ORAI3	3.91E-11	0.456541433	0.5	0.037	8.15E-07
MRPL38	1.38E-18	0.455772225	0.591	0	2.86E-14
TMEM35B	1.38E-14	0.452361309	0.477	0	2.88E-10
SMARCE1	7.28E-13	0.451783631	0.659	0.084	1.52E-08
FLI1	6.69E-09	0.449932278	0.773	0.243	0.000139267
MPP1	3.40E-09	0.447724236	0.682	0.168	7.07E-05
NFATC2	1.05E-10	0.447323621	0.659	0.121	2.18E-06
HLA-B	1.58E-06	0.446659908	1	0.953	0.032982496
SARNP	5.69E-13	0.44614143	0.568	0.037	1.18E-08
PMPCB	3.61E-12	0.444508762	0.909	0.318	7.51E-08
STAT1	9.77E-07	0.44379758	0.455	0.075	0.020331731
PSMB8	7.50E-07	0.442980628	0.909	0.477	0.015613984
SH3BGRL3	1.30E-06	0.442724972	1	0.832	0.027169138
ARPC5	5.85E-07	0.442499835	0.841	0.383	0.012171257
SHMT2	5.55E-11	0.440974356	0.614	0.084	1.15E-06
KIF2A	2.46E-12	0.440172073	0.705	0.121	5.11E-08
ATP5PB	3.57E-07	0.438244	0.886	0.43	0.007429455
LINC02256	1.66E-14	0.438208581	0.705	0.093	3.46E-10
PRMT2	1.36E-07	0.437000339	0.705	0.224	0.002839214
CNN2	1.89E-08	0.434647064	0.75	0.243	0.000392631
NDUFV1	1.04E-06	0.434488766	0.75	0.29	0.021555743
LGALS9	3.59E-08	0.433425146	0.818	0.318	0.000746882
IL16	1.13E-08	0.430434216	0.568	0.103	0.000235624
LCP1	1.00E-08	0.427411347	0.818	0.299	0.000208506
CD109	1.55E-07	0.427176727	0.5	0.084	0.003221914
GRSF1	1.01E-10	0.425372779	0.705	0.159	2.10E-06
RBBP4	4.09E-07	0.423669745	0.773	0.299	0.008511011
TRIM58	7.19E-10	0.42226622	0.545	0.075	1.50E-05
GNG10	1.75E-10	0.421976822	0.455	0.019	3.63E-06
C20orf27	5.02E-08	0.4208984	0.75	0.252	0.001045115
CAPRIN1	4.99E-10	0.420589513	0.795	0.243	1.04E-05
NBPF26	1.41E-12	0.420388347	0.523	0.019	2.93E-08
BCAP31	1.48E-10	0.420370468	0.841	0.28	3.09E-06
SQSTM1	1.17E-08	0.419999091	0.841	0.327	0.000244288

SNAP23	9.17E-13	0.419777353	0.705	0.131	1.91E-08
NUMA1	2.58E-07	0.415491499	0.477	0.075	0.005364894
ATRX	4.71E-08	0.414968381	0.886	0.402	0.000980082
SMARCA2	4.76E-11	0.414912586	0.75	0.178	9.90E-07
CBR1	1.20E-07	0.412532268	0.455	0.056	0.002493663
ZKSCAN1	2.25E-06	0.411402001	0.705	0.262	0.046925549
TMC8	1.13E-06	0.409235761	0.636	0.196	0.023517868
ACAP2	6.61E-11	0.408367554	0.705	0.15	1.38E-06
SYK	6.62E-09	0.405966714	0.659	0.168	0.000137771
NRDC	1.23E-06	0.405837826	0.614	0.178	0.025595413
FDFT1	8.25E-10	0.405773387	0.75	0.215	1.72E-05
NT5C	1.58E-06	0.404947803	0.659	0.215	0.032934265
ZNF706	3.78E-07	0.40456279	0.841	0.374	0.007866398
FNTA	6.52E-12	0.403962117	0.636	0.093	1.36E-07
MRPL18	1.54E-09	0.40267663	0.636	0.131	3.21E-05
FANCL	1.76E-08	0.402221253	0.386	0.019	0.00036692
AGPAT5	4.49E-13	0.401553986	0.591	0.047	9.35E-09
CDC37	1.10E-11	0.401066079	0.932	0.355	2.30E-07
GCA	1.47E-10	0.400304936	0.523	0.047	3.06E-06
PLEK	1.74E-06	0.398158301	0.614	0.187	0.036284856
SSR1	2.19E-06	0.397317177	0.727	0.28	0.045514075
RAB24	4.23E-13	0.396313651	0.545	0.028	8.80E-09
HIGD2A	1.34E-07	0.395966205	0.977	0.57	0.002783451
BCKDHA	7.81E-14	0.395781627	0.455	0	1.63E-09
GPAT3	5.74E-10	0.394284733	0.545	0.075	1.20E-05
SRSF10	3.69E-08	0.392361845	0.841	0.346	0.000768744
PRRC2B	2.46E-07	0.391323216	0.5	0.093	0.005129083
CRTAP	2.81E-10	0.390779543	0.705	0.178	5.85E-06
PSMA5	7.23E-08	0.389375447	0.795	0.308	0.001504307
GNB2	2.19E-06	0.388938285	0.591	0.168	0.045576177
UFM1	1.52E-09	0.388527748	0.705	0.178	3.17E-05
RAB11B	6.95E-10	0.387871197	0.75	0.215	1.45E-05
GUCY1A1	1.14E-06	0.387158464	0.614	0.187	0.023773695
SOD2	8.51E-08	0.387016185	0.773	0.29	0.001771399
RBPMS	8.05E-08	0.386220693	0.682	0.206	0.001676683
NGRN	8.81E-11	0.386110028	0.432	0.009	1.83E-06
SYNGR2	3.31E-08	0.384456602	0.591	0.131	0.000689263
PDCD2	6.52E-07	0.384272723	0.795	0.336	0.013575911
RALY	8.31E-08	0.383464382	0.795	0.308	0.001730426
HPS1	2.08E-10	0.383376645	0.636	0.112	4.33E-06
HMGN4	2.26E-12	0.382097861	0.409	0	4.70E-08
ECI2	6.55E-07	0.381118803	0.614	0.178	0.013634754
HIST1H2BK	2.26E-12	0.381069544	0.409	0	4.70E-08
TGIF2	9.95E-12	0.380986308	0.5	0.028	2.07E-07
UROD	1.89E-07	0.380167343	0.727	0.252	0.003932253
NOB1	7.13E-11	0.378810379	0.818	0.262	1.48E-06
HACD4	3.77E-07	0.377366033	0.659	0.206	0.007858057
CHST12	1.25E-06	0.377222837	0.75	0.299	0.025969717
JAK1	1.84E-06	0.376887522	0.773	0.327	0.038360932
NDUFC1	9.06E-08	0.376389034	0.909	0.439	0.001886816
MLF2	8.52E-10	0.376089994	0.864	0.327	1.77E-05
UQCRC1	2.09E-10	0.374553337	0.705	0.168	4.36E-06
MRPS27	1.72E-10	0.374128248	0.705	0.168	3.57E-06
REST	2.01E-07	0.37398909	0.727	0.252	0.00417473
ARL6IP5	1.31E-07	0.372512382	0.886	0.421	0.002737398
KCNAB2	6.00E-10	0.370862879	0.682	0.159	1.25E-05

OGT	2.56E-10	0.370349257	0.614	0.112	5.32E-06
MRPS18B	2.56E-12	0.36992264	0.659	0.112	5.33E-08
PECAM1	1.36E-09	0.369122312	0.636	0.14	2.82E-05
GPSM3	2.47E-07	0.369108951	0.909	0.458	0.00514982
RGS19	1.57E-06	0.36844176	0.659	0.224	0.032645594
PTOV1	1.06E-07	0.368199273	0.591	0.14	0.002214206
DAPK1	3.37E-08	0.367819264	0.659	0.187	0.00070135
AGGF1	2.98E-13	0.367497222	0.5	0.019	6.21E-09
GRB2	3.58E-10	0.367470558	0.682	0.159	7.45E-06
C14orf119	3.21E-07	0.366607472	0.455	0.065	0.006686172
SLC25A36	4.29E-07	0.365569414	0.636	0.187	0.008937607
CD82	1.44E-07	0.365181658	0.727	0.252	0.003004676
UNC13D	2.32E-07	0.364941385	0.614	0.168	0.004839859
PPP1CA	9.67E-11	0.364454645	0.864	0.318	2.01E-06
DELE1	3.25E-07	0.364430966	0.545	0.121	0.006761971
ABHD14B	5.41E-07	0.363912032	0.932	0.505	0.011272711
MMS19	8.43E-13	0.363903778	0.568	0.037	1.76E-08
SDHD	1.73E-12	0.363548356	0.75	0.178	3.59E-08
SNX5	1.60E-10	0.36321153	0.705	0.178	3.34E-06
LRRFIP2	1.95E-08	0.363164118	0.705	0.215	0.00040594
UBXN4	1.63E-06	0.362512285	0.795	0.355	0.033988937
DCTD	8.03E-11	0.3624837	0.614	0.112	1.67E-06
PSMG2	6.17E-08	0.362246314	0.818	0.336	0.001284988
FLT3	9.66E-08	0.361967269	0.523	0.093	0.002012249
TAGAP	2.34E-09	0.361429756	0.477	0.047	4.87E-05
C5orf51	7.11E-08	0.360795678	0.386	0.028	0.001479343
NIPSNAP1	1.16E-07	0.360749505	0.568	0.131	0.002410921
NREP	2.31E-06	0.36061052	0.659	0.234	0.048007693
DYRK4	1.18E-10	0.359948757	0.477	0.037	2.47E-06
CAVIN1	7.74E-07	0.3585775	0.682	0.252	0.016114281
TNFSF13	2.26E-12	0.358337086	0.409	0	4.70E-08
RABGAP1	4.72E-11	0.357378315	0.659	0.131	9.83E-07
EPN1	8.99E-09	0.35729792	0.523	0.093	0.000187235
IVD	6.45E-10	0.356532893	0.477	0.037	1.34E-05
STIM1	1.55E-07	0.356354399	0.455	0.065	0.003222005
PSMB10	1.93E-06	0.355713288	0.75	0.308	0.040181452
PRPF8	5.87E-08	0.35531723	0.614	0.159	0.001221977
TIMM17B	2.09E-12	0.354998445	0.614	0.084	4.35E-08
HAUS4	8.48E-11	0.35481835	0.568	0.075	1.77E-06
UPF3A	8.23E-08	0.354293131	0.659	0.196	0.001713962
TOP1	1.72E-09	0.352940224	0.682	0.187	3.58E-05
FPGS	1.08E-10	0.351672808	0.545	0.065	2.26E-06
CASP1	1.68E-07	0.351654808	0.477	0.075	0.003499347
SRSF1	2.43E-09	0.350450053	0.659	0.168	5.06E-05
FAM96A	2.12E-11	0.349053866	0.818	0.262	4.41E-07
IRF2	1.34E-09	0.348237443	0.523	0.065	2.78E-05
RNF24	3.63E-09	0.347907428	0.591	0.131	7.56E-05
GRN	2.68E-10	0.347209005	0.727	0.196	5.58E-06
IFNGR2	1.06E-11	0.346049175	0.591	0.084	2.20E-07
MGLL	1.32E-08	0.34578982	0.591	0.131	0.00027446
CPXM1	4.39E-07	0.344821897	0.545	0.131	0.009137609
ANKRD10	5.52E-08	0.344186395	0.636	0.168	0.001149373
FBXL5	5.93E-11	0.34365243	0.636	0.112	1.23E-06
USP11	2.98E-10	0.343617869	0.614	0.112	6.20E-06
SELENOW	1.80E-08	0.34360008	0.841	0.346	0.000374389
RTL8A	3.07E-08	0.343230876	0.523	0.093	0.000639826

USP10	4.31E-07	0.342861491	0.477	0.084	0.00896659
PLSCR1	7.49E-08	0.342582721	0.636	0.178	0.001558419
ATP6AP1	7.19E-13	0.342581981	0.682	0.112	1.50E-08
TECR	2.29E-06	0.342216871	0.682	0.252	0.047781672
RAB10	6.68E-10	0.342185613	0.591	0.103	1.39E-05
HEBP1	9.80E-07	0.34210073	0.818	0.374	0.020402136
RPN1	1.11E-08	0.340126913	0.614	0.15	0.000230301
KEAP1	1.31E-09	0.340012811	0.568	0.093	2.73E-05
RNF187	4.37E-08	0.339533354	0.682	0.206	0.000910277
RAD23A	1.09E-06	0.338620149	0.818	0.374	0.022700021
RPS26	4.49E-08	0.338362405	1	1	0.000935148
UBE2L6	1.54E-06	0.337668404	0.795	0.355	0.032086437
WDR77	7.10E-10	0.337508433	0.432	0.019	1.48E-05
SEPHS2	1.77E-12	0.336991666	0.727	0.159	3.68E-08
CIAO1	1.53E-11	0.336978811	0.568	0.065	3.18E-07
WASHC4	1.64E-11	0.336951976	0.614	0.093	3.41E-07
COX16	2.13E-11	0.33683791	0.568	0.075	4.43E-07
RCN1	1.05E-06	0.336602799	0.614	0.187	0.021846365
UBR5-AS1	7.11E-07	0.335614488	0.455	0.075	0.014804086
PRRC2A	2.17E-06	0.335605983	0.5	0.112	0.04527201
PSIP1	6.98E-09	0.335283037	0.909	0.43	0.000145371
PIGM	7.63E-08	0.335270524	0.455	0.065	0.001588673
ISCU	4.52E-08	0.334760435	0.841	0.364	0.0009413
ABTB1	2.34E-11	0.334723751	0.5	0.037	4.87E-07
STAT5A	9.96E-07	0.334723713	0.705	0.262	0.020740246
RASAL3	8.32E-08	0.334655973	0.523	0.093	0.001732555
GPS1	5.33E-10	0.334537021	0.545	0.084	1.11E-05
MLX	1.10E-08	0.334354189	0.5	0.065	0.000229887
IFITM2	1.94E-06	0.334037106	0.932	0.533	0.040367819
PTPN6	5.75E-07	0.334030373	0.75	0.299	0.01197759
HDAC2	2.93E-08	0.333577844	0.773	0.29	0.000609694
AKIRIN2	3.75E-09	0.333536309	0.523	0.075	7.81E-05
SF3A2	6.01E-08	0.332976562	0.682	0.215	0.001251304
LMAN1	9.19E-07	0.331851198	0.705	0.262	0.019131656
RNF220	8.29E-08	0.331781707	0.659	0.206	0.001725702
RAB14	3.09E-08	0.331470837	0.568	0.121	0.000642736
PRDX3	9.80E-10	0.330738367	0.795	0.28	2.04E-05
AHCY	4.43E-07	0.328225691	0.682	0.234	0.009232335
OXLD1	6.31E-07	0.32813805	0.545	0.131	0.013145902
ATF2	1.98E-10	0.328047855	0.477	0.037	4.13E-06
EBLN3P	4.44E-08	0.327260915	0.568	0.121	0.000925359
TMPO	2.49E-07	0.327223597	0.523	0.112	0.005189711
NMI	5.84E-07	0.326986791	0.659	0.224	0.012165451
CAPZB	5.30E-07	0.326833741	0.886	0.449	0.011030549
ZNF302	4.82E-08	0.326727474	0.523	0.093	0.00100386
MED16	2.63E-08	0.326541616	0.409	0.028	0.000546982
RAB7A	1.16E-08	0.326187425	0.727	0.243	0.000242043
SCHIP1	2.06E-09	0.325316548	0.432	0.037	4.29E-05
BSCL2	2.33E-09	0.324933289	0.409	0.019	4.85E-05
LINC01089	6.78E-10	0.324904909	0.5	0.056	1.41E-05
DHRS4	9.58E-08	0.324278869	0.568	0.131	0.001995455
SHKBP1	2.44E-07	0.324105274	0.591	0.159	0.005076653
DNAJC1	1.87E-08	0.323160213	0.682	0.206	0.000389864
IRF5	2.55E-11	0.321669206	0.568	0.075	5.32E-07
NIPBL	3.58E-11	0.321386446	0.568	0.084	7.45E-07
ANXA6	7.23E-08	0.320269291	0.568	0.131	0.001505878

ISYNA1	4.09E-07	0.319957884	0.636	0.196	0.008509705
RANGRF	1.34E-08	0.319913831	0.568	0.121	0.000277995
OSBPL9	7.84E-09	0.319575079	0.523	0.084	0.000163304
RCN2	2.10E-07	0.319212677	0.727	0.271	0.004367204
SCNM1	2.84E-11	0.319149059	0.568	0.075	5.90E-07
SORL1	4.37E-07	0.318524546	0.705	0.28	0.009103044
ASF1A	6.31E-08	0.318447042	0.5	0.075	0.001314362
CXXC5	3.30E-09	0.317755744	0.614	0.14	6.87E-05
MGMT	1.89E-07	0.31708102	0.659	0.215	0.003928408
TMEM173	8.11E-11	0.316926994	0.909	0.374	1.69E-06
RNPS1	4.46E-07	0.31648929	0.932	0.523	0.009283778
C19orf66	4.12E-08	0.315746169	0.636	0.178	0.000858177
ATM	8.93E-09	0.31553967	0.455	0.056	0.000185914
PPP1R12A	9.13E-07	0.31552	0.705	0.271	0.019014027
RHOG	3.99E-07	0.315347708	0.795	0.346	0.008302274
HADH	4.65E-08	0.315131645	0.568	0.131	0.000967999
CUX1	1.36E-06	0.315124059	0.636	0.215	0.028222107
NSFL1C	2.08E-08	0.315054254	0.636	0.178	0.000432663
FAM120AOS	6.00E-09	0.314989983	0.477	0.047	0.000124869
ZFAND2B	1.36E-06	0.314498269	0.545	0.15	0.028318994
RANBP2	3.90E-09	0.314485422	0.614	0.14	8.13E-05
TMEM219	3.16E-08	0.314469198	0.864	0.393	0.000657034
C11orf1	1.86E-06	0.313814536	0.682	0.252	0.038815511
EMD	5.21E-07	0.312997631	0.545	0.14	0.010848511
UBE2E1	1.10E-08	0.312743167	0.75	0.262	0.000230057
SNRNP40	3.34E-10	0.312670625	0.614	0.121	6.95E-06
MRPL44	1.39E-13	0.312622642	0.591	0.056	2.89E-09
ADH5	3.81E-11	0.312540253	0.682	0.159	7.93E-07
MLEC	6.48E-10	0.312452131	0.659	0.159	1.35E-05
MFSD14A	5.79E-11	0.312340174	0.364	0	1.21E-06
GNL3	4.98E-11	0.31218804	0.75	0.215	1.04E-06
C11orf98	5.63E-11	0.311502417	0.432	0.009	1.17E-06
AC116366.3	2.30E-10	0.311207225	0.409	0.009	4.79E-06
MEA1	6.06E-10	0.310896118	0.727	0.206	1.26E-05
ANAPC5	2.80E-07	0.309474709	0.841	0.393	0.005821455
SDHA	3.39E-09	0.309437205	0.568	0.112	7.06E-05
EEF1A1	5.47E-12	0.308912267	1	1	1.14E-07
MRPL37	1.82E-08	0.307860688	0.568	0.121	0.000379184
GCNT1	3.60E-08	0.306972346	0.386	0.028	0.000750062
ATAD1	1.11E-07	0.30685593	0.5	0.093	0.002303404
METTL3	5.27E-07	0.306394778	0.386	0.037	0.010971633
TESMIN	6.96E-07	0.306244854	0.455	0.075	0.014489075
OAS2	3.20E-07	0.305619225	0.341	0.019	0.006652241
ISCA2	2.02E-07	0.305505313	0.5	0.093	0.004215351
TMEM69	3.85E-10	0.30536831	0.568	0.103	8.02E-06
ZEB2	5.74E-08	0.304849584	0.705	0.243	0.001195329
SPTLC2	8.44E-10	0.304701724	0.636	0.159	1.76E-05
C1orf43	9.81E-09	0.304428155	0.886	0.402	0.000204199
CDC73	2.24E-06	0.303886152	0.455	0.093	0.046640457
CITED2	1.98E-06	0.303876271	0.614	0.206	0.041302034
XAF1	4.35E-07	0.30355936	0.5	0.103	0.009051953
TIA1	4.39E-08	0.303541574	0.523	0.103	0.000914261
NIFK	1.02E-07	0.303234584	0.614	0.178	0.002129058
SLC25A46	2.74E-08	0.302314799	0.409	0.028	0.000571008
NDUFB10	1.62E-07	0.301684576	0.886	0.439	0.003375976
ROCK1	1.03E-07	0.300914886	0.818	0.355	0.002153477

EGLN2	1.47E-07	0.300423664	0.318	0.009	0.003062161
FLOT2	2.24E-08	0.300329882	0.5	0.084	0.000467129
METAP1	2.30E-09	0.300276124	0.477	0.047	4.79E-05
ARL11	1.94E-11	0.300077922	0.5	0.037	4.03E-07
FAM208A	9.60E-10	0.299800376	0.455	0.037	2.00E-05
SMARCC2	1.83E-10	0.299626737	0.591	0.121	3.80E-06
ABHD16A	1.34E-09	0.299477748	0.318	0	2.78E-05
SMARCB1	1.61E-08	0.29938966	0.705	0.234	0.000336187
FIRRE	6.81E-10	0.2990426	0.432	0.019	1.42E-05
DCTN2	8.60E-09	0.298644694	0.841	0.355	0.000179003
MKRN1	3.81E-08	0.2984074	0.682	0.224	0.000793374
VSIR	4.57E-08	0.29744096	0.614	0.168	0.000952193
HOXA9	6.21E-07	0.297079469	0.523	0.131	0.012932819
ETHE1	1.31E-07	0.296402909	0.705	0.252	0.002736685
NDUFAF3	1.54E-10	0.296102995	0.841	0.308	3.21E-06
UTY	8.14E-07	0.295686908	0.341	0.028	0.016950629
TBC1D5	9.65E-13	0.295590192	0.614	0.103	2.01E-08
DEF6	5.29E-13	0.295307947	0.636	0.112	1.10E-08
DPYD	4.23E-08	0.295244816	0.5	0.084	0.000881251
BIN2	4.56E-07	0.295123968	0.659	0.224	0.009494031
RNF114	2.77E-09	0.295092695	0.705	0.224	5.76E-05
PGAM1	1.20E-06	0.294812424	0.682	0.262	0.025030961
AASDHPPT	3.78E-11	0.293376904	0.523	0.056	7.87E-07
RSPRY1	1.30E-09	0.29303378	0.477	0.047	2.71E-05
SLC25A11	1.22E-08	0.292794347	0.545	0.103	0.000253203
MRPL3	3.19E-09	0.292478293	0.659	0.178	6.64E-05
IST1	2.87E-09	0.292439755	0.5	0.075	5.98E-05
CRLS1	9.95E-07	0.292345209	0.432	0.075	0.020707474
SHARPIN	9.20E-07	0.291898529	0.455	0.084	0.019149933
MSRA	5.05E-08	0.291804266	0.5	0.084	0.001050848
RTN3	1.20E-08	0.291736265	0.773	0.299	0.000250848
OLA1	2.22E-07	0.291587659	0.955	0.542	0.004616345
PARP10	1.71E-07	0.29093299	0.364	0.028	0.003556719
GSN	9.15E-11	0.290727064	0.75	0.234	1.90E-06
DCAF12	4.91E-10	0.290656856	0.5	0.056	1.02E-05
HSPA4	7.94E-08	0.289875908	0.455	0.065	0.00165401
PPP1CC	1.25E-06	0.289428247	0.818	0.393	0.0261012
ZNF493	5.35E-07	0.289222404	0.432	0.065	0.011136819
BTN3A2	3.72E-08	0.288901341	0.591	0.159	0.000774409
ZSCAN18	1.63E-11	0.288850741	0.523	0.065	3.40E-07
PLXND1	1.22E-10	0.288473661	0.455	0.028	2.54E-06
DDX42	1.55E-08	0.288084163	0.409	0.028	0.00032167
SRSF4	2.47E-08	0.28791378	0.659	0.206	0.000513359
VDAC3	2.24E-07	0.287532102	0.795	0.346	0.004653386
AC246787.2	2.80E-08	0.287327896	0.273	0	0.000582498
RAB2A	1.91E-06	0.287277693	0.659	0.243	0.039798279
WDR1	2.89E-07	0.287058249	0.591	0.187	0.006006582
TM7SF3	6.37E-08	0.286805161	0.818	0.364	0.001326568
TPT1	5.98E-07	0.286651015	1	1	0.012456674
GOPC	1.26E-07	0.286449838	0.545	0.131	0.002622335
CD46	2.85E-09	0.28606991	0.5	0.084	5.93E-05
NACA	1.29E-06	0.285771412	1	1	0.026917571
PLSCR3	4.70E-10	0.285694795	0.386	0.009	9.78E-06
ANXA4	2.47E-08	0.285684002	0.545	0.121	0.000513925
NIPSNAP3A	1.03E-11	0.285360643	0.682	0.159	2.14E-07
RNF149	1.35E-06	0.285182385	0.341	0.028	0.028162001

P2RY8	1.29E-08	0.285149332	0.455	0.065	0.000269316
NAPA	2.84E-09	0.284880708	0.591	0.131	5.91E-05
FGFR1OP2	1.81E-07	0.284765055	0.841	0.383	0.003778514
TIPRL	5.14E-07	0.284711229	0.523	0.131	0.01069795
POLR2D	1.18E-10	0.284625337	0.659	0.15	2.45E-06
MACF1	1.09E-06	0.284423881	0.727	0.29	0.022683769
MRFAP1L1	9.57E-10	0.283827664	0.523	0.084	1.99E-05
TMEM223	2.40E-08	0.283486802	0.432	0.047	0.000499699
ARHGEF3	3.01E-07	0.28279479	0.386	0.037	0.006273525
NAP1L4	1.18E-06	0.282561484	0.568	0.168	0.024520511
AC090673.1	6.18E-09	0.282393657	0.295	0	0.000128748
ANXA11	1.02E-10	0.281989624	0.818	0.28	2.12E-06
MKKS	1.39E-07	0.281928321	0.432	0.056	0.002896695
NR3C1	5.21E-08	0.281827023	0.614	0.178	0.001085128
NDFIP1	1.81E-10	0.281475812	0.773	0.252	3.76E-06
CHTF8	1.40E-06	0.281354359	0.477	0.103	0.029108117
PPM1G	4.76E-07	0.28131646	0.727	0.29	0.009912341
BCL2L1	1.19E-06	0.281201584	0.455	0.084	0.024680812
PPT1	6.71E-07	0.281000786	0.659	0.234	0.013975951
XRCC6	2.24E-06	0.280638998	0.841	0.421	0.046539777
VTA1	4.38E-09	0.280636225	0.523	0.093	9.12E-05
PPP2R1A	8.02E-08	0.28034874	0.727	0.29	0.001670634
HSD17B4	6.38E-10	0.279706406	0.477	0.056	1.33E-05
CDK5RAP3	6.17E-07	0.279474614	0.636	0.215	0.012842663
CDC123	1.32E-06	0.279442898	0.5	0.121	0.027521196
U2AF1	1.64E-06	0.279353041	0.364	0.037	0.034059763
RBM5	1.65E-06	0.277991652	0.568	0.178	0.034380014
RPP21	6.01E-09	0.277885445	0.432	0.037	0.000125175
TMED3	2.28E-06	0.277768778	0.841	0.421	0.047562202
ACBD6	2.07E-06	0.277586502	0.591	0.187	0.043112985
STAM	2.33E-06	0.277534936	0.386	0.056	0.048417202
DOCK8	1.70E-07	0.277026658	0.591	0.178	0.003545667
RWDD3	5.79E-11	0.276705781	0.364	0	1.21E-06
RGS14	6.76E-11	0.276673078	0.523	0.065	1.41E-06
KIT	8.80E-08	0.276656267	0.477	0.084	0.001831135
RCSD1	9.62E-09	0.276372978	0.841	0.355	0.000200278
ACY1	3.42E-08	0.276190813	0.341	0.009	0.000711322
LMO4	1.62E-07	0.275451471	0.477	0.103	0.003369105
RPL7A	6.06E-09	0.274249986	1	1	0.000126191
EDEM2	1.04E-11	0.273819701	0.5	0.047	2.16E-07
MRPL24	9.28E-07	0.273323036	0.477	0.103	0.019314986
RFK	9.74E-07	0.272986524	0.477	0.103	0.020286381
NUP37	2.06E-06	0.272986184	0.364	0.047	0.042972462
FAAP20	3.23E-10	0.272614235	0.818	0.308	6.72E-06
HECTD1	1.53E-09	0.272415405	0.591	0.14	3.20E-05
POLR2E	8.33E-08	0.272223091	0.682	0.234	0.001733735
NOA1	2.06E-06	0.272158615	0.636	0.234	0.042949854
PIK3CD	1.71E-07	0.272083398	0.386	0.037	0.003558875
CAPG	4.62E-09	0.271767475	0.591	0.15	9.61E-05
PLAGL1	4.58E-10	0.271671044	0.659	0.187	9.54E-06
TCTEX1D1	1.13E-08	0.271384432	0.477	0.075	0.000234806
C1orf21	1.38E-07	0.271375326	0.659	0.234	0.002878311
STX12	7.20E-10	0.271242576	0.455	0.037	1.50E-05
FAM219B	3.77E-08	0.271180193	0.523	0.112	0.000784387
VAT1	1.10E-08	0.270968008	0.477	0.065	0.000228808
METTL17	1.45E-06	0.270816554	0.432	0.075	0.030275214

EDC4	5.79E-11	0.270744598	0.364	0	1.21E-06
PTGER2	3.65E-07	0.270610273	0.545	0.15	0.007605772
COASY	4.02E-10	0.27060799	0.455	0.037	8.36E-06
SLBP	1.56E-08	0.270380982	0.636	0.187	0.000324423
ETS2	8.38E-10	0.269476321	0.75	0.252	1.74E-05
SMIM29	1.10E-07	0.26912403	0.455	0.075	0.002300161
SMG1	2.24E-08	0.268247283	0.545	0.121	0.000466043
BRWD1	2.98E-08	0.268238808	0.545	0.131	0.00062074
CHD2	6.41E-07	0.268159407	0.568	0.168	0.013351808
ZNF524	1.57E-08	0.267938267	0.386	0.028	0.000327672
CHST13	5.46E-09	0.267307268	0.523	0.093	0.000113685
MRPL34	6.40E-10	0.267198427	0.727	0.224	1.33E-05
PAF1	5.75E-10	0.267120673	0.477	0.047	1.20E-05
FSCN1	9.27E-08	0.266987045	0.591	0.168	0.001930012
YIF1B	3.20E-07	0.266872506	0.455	0.075	0.00666975
CHD1L	9.84E-09	0.266493269	0.591	0.15	0.000204925
AL365205.1	3.64E-12	0.266410688	0.432	0.009	7.59E-08
KAT6B	9.67E-09	0.266357881	0.568	0.131	0.000201313
IGBP1	8.60E-07	0.266292148	0.818	0.402	0.017896418
ARL5A	2.44E-10	0.265429643	0.591	0.121	5.08E-06
RAB6A	1.90E-09	0.265314294	0.5	0.075	3.96E-05
APPL1	9.66E-08	0.26508163	0.75	0.299	0.002011832
CREG1	3.56E-07	0.264728799	0.795	0.355	0.007404597
POP5	4.65E-07	0.264712664	0.545	0.159	0.009672285
ILK	4.89E-08	0.264587502	0.477	0.075	0.001018616
TNFAIP8L2	2.80E-08	0.262527964	0.273	0	0.000582498
TSC22D1	2.11E-07	0.262230843	0.773	0.327	0.004384794
PDHB	1.26E-07	0.261995966	0.477	0.093	0.002626676
CASP4	2.73E-07	0.261462225	0.841	0.411	0.00567649
TBRG1	9.06E-11	0.261423467	0.545	0.093	1.89E-06
COMMD2	1.37E-07	0.261348736	0.591	0.178	0.002860425
SIGMAR1	1.08E-06	0.261347023	0.364	0.037	0.022544463
CTSB	1.50E-10	0.261264293	0.545	0.103	3.13E-06
DUS3L	2.50E-08	0.261021105	0.455	0.056	0.00051957
BAX	8.89E-08	0.260929345	0.795	0.336	0.001851113
SCAMP3	1.55E-07	0.260696058	0.477	0.093	0.003231886
ALDH9A1	4.22E-10	0.260689552	0.591	0.131	8.78E-06
PRPF4B	9.01E-13	0.260316087	0.682	0.15	1.88E-08
TTC9C	3.72E-11	0.260177126	0.523	0.065	7.75E-07
NKIRAS2	4.72E-07	0.260090373	0.455	0.093	0.009828642
NUDT2	4.76E-11	0.259802718	0.614	0.121	9.90E-07
PRMT6	1.17E-07	0.259609808	0.364	0.028	0.00243421
SPG21	1.50E-09	0.259512319	0.841	0.355	3.13E-05
LRP10	1.87E-08	0.259403072	0.455	0.065	0.00038968
HLTF	6.50E-08	0.258989324	0.364	0.028	0.001353887
C11orf49	2.83E-07	0.258906048	0.364	0.028	0.005894647
POLB	7.61E-09	0.258121892	0.409	0.037	0.00015853
SNRNP70	7.55E-08	0.258105496	0.659	0.224	0.001571196
ANP32A	1.39E-06	0.257802294	0.773	0.364	0.028971541
THOC6	1.88E-07	0.257562172	0.477	0.103	0.003911593
STARD7	2.71E-07	0.257548914	0.591	0.187	0.005637738
LETMD1	1.95E-09	0.257471266	0.727	0.252	4.06E-05
UBE2J1	1.66E-06	0.257411385	0.75	0.327	0.034597542
ROBO4	2.76E-08	0.257342732	0.455	0.075	0.00057515
ERCC5	2.82E-10	0.257099386	0.341	0	5.87E-06
RER1	6.59E-07	0.257074369	0.545	0.159	0.013728871

UBXN7	2.14E-06	0.256785207	0.364	0.047	0.044591248
TRABD	1.01E-08	0.256771327	0.614	0.178	0.000210544
MAN2A2	8.72E-09	0.255992743	0.477	0.084	0.000181649
MRPL41	6.71E-07	0.255986942	0.523	0.14	0.013966123
GATA2	3.83E-07	0.255868021	0.727	0.308	0.007971817
SAFB	1.78E-08	0.255837391	0.523	0.112	0.000369878
ADSS	1.32E-09	0.255435277	0.614	0.159	2.76E-05
MPHOSPH8	2.94E-07	0.255348151	0.705	0.28	0.006116163
JAGN1	1.24E-06	0.255222596	0.705	0.29	0.025880161
PLSCR4	4.39E-07	0.255160753	0.295	0.009	0.009141141
SEC14L1	3.12E-08	0.255056672	0.5	0.103	0.000650464
GNPTG	6.22E-10	0.254821783	0.432	0.037	1.29E-05
BRIX1	2.26E-12	0.25437196	0.614	0.103	4.71E-08
TRIM5	5.56E-07	0.254352692	0.386	0.047	0.011575652
MRPL28	1.95E-07	0.253914721	0.591	0.178	0.004057418
PIGC	1.65E-07	0.253815773	0.5	0.103	0.003427993
TRIP12	2.44E-07	0.253530722	0.432	0.065	0.005089453
CBWD5	1.00E-08	0.253137336	0.432	0.047	0.000208755
MAN2B1	3.22E-07	0.252218568	0.341	0.028	0.006710075
MTCH2	1.07E-07	0.252021577	0.5	0.112	0.002231243
DNAJC4	2.73E-07	0.251810654	0.886	0.458	0.005690807
DTWD1	2.96E-07	0.251768936	0.5	0.112	0.006170838
TMEM220	1.10E-06	0.251437015	0.545	0.168	0.022995399
COPA	3.96E-07	0.251279145	0.545	0.15	0.00823507
RTL8C	8.24E-07	0.251137827	0.477	0.112	0.017163705
NOSIP	2.63E-09	0.251002816	0.636	0.178	5.48E-05
SSBP1	1.92E-08	0.250891301	0.955	0.514	0.000399693
VPS26B	6.89E-08	0.250887075	0.523	0.131	0.001434162
PABPN1	8.30E-07	0.250868802	0.682	0.262	0.017288617
ARHGAP1	5.49E-07	0.250832297	0.409	0.065	0.011427235
MRPS34	7.71E-07	0.250539064	0.591	0.196	0.016057175
EIF2AK4	2.14E-06	0.250313499	0.386	0.065	0.044570179
RPL18A	2.29E-09	-0.269247635	1	1	4.76E-05
DNAJB6	3.22E-09	-0.273919576	0.909	0.71	6.70E-05
RPL39	1.17E-11	-0.293537344	1	1	2.45E-07
ATF4	6.63E-07	-0.296973931	0.705	0.626	0.013800224
RPL36	8.97E-13	-0.339912347	1	1	1.87E-08
RPL15	4.38E-13	-0.340256388	1	1	9.12E-09
IGSF10	2.01E-06	-0.345011877	0.409	0.364	0.041911383
RPS18	1.98E-14	-0.36062476	1	1	4.12E-10
RPL10A	1.10E-13	-0.370673859	0.977	1	2.29E-09
PAPOLA	2.46E-09	-0.378126692	0.659	0.598	5.13E-05
BTG1	7.27E-07	-0.41279941	0.795	0.664	0.015126001
RPL35	1.79E-18	-0.425744765	1	1	3.72E-14
RPS16	6.66E-08	-0.434626475	1	1	0.001386883
RPS6	2.76E-19	-0.442098474	1	1	5.74E-15
NDUFB1	3.62E-08	-0.461136083	0.682	0.654	0.000753548
VAMP2	7.29E-08	-0.463377691	0.636	0.664	0.001518674
RPL34	9.23E-22	-0.467678002	1	1	1.92E-17
NPM3	4.37E-07	-0.469569887	0.227	0.43	0.009101876
YPEL5	1.21E-09	-0.489121901	0.727	0.579	2.53E-05
CYCS	1.57E-09	-0.501820235	0.659	0.645	3.27E-05
ARL4A	6.03E-08	-0.510945675	0.5	0.551	0.001256219
HNRNPA1P48	4.21E-07	-0.514322246	0.023	0.374	0.008758353
SELENOK	6.49E-07	-0.520074703	0.477	0.579	0.013518915
IGKC	5.17E-07	-0.533959652	0	0.308	0.010763774

FOS	1.12E-10	-0.53468688	0.864	0.991	2.33E-06
CCNL1	1.02E-06	-0.543386434	0.682	0.766	0.021183492
FTH1	5.79E-08	-0.547383561	1	0.991	0.001204999
UBB	9.34E-07	-0.554536381	0.932	0.972	0.019450314
EIF1	1.07E-10	-0.557345498	1	1	2.23E-06
SNHG8	6.62E-10	-0.566401531	1	0.981	1.38E-05
RPL37A	1.12E-22	-0.573210267	1	1	2.32E-18
SNRPE	1.49E-09	-0.601231953	0.864	0.832	3.10E-05
PSMA2	9.82E-09	-0.610442069	0.364	0.561	0.000204547
RPS27	1.88E-31	-0.644886233	1	1	3.91E-27
RPL23A	3.16E-34	-0.649321464	0.977	1	6.58E-30
RPL9	9.66E-34	-0.669418849	1	1	2.01E-29
CHMP1B	2.28E-07	-0.720781071	0.386	0.617	0.004749659
RPL36A	1.05E-21	-0.746464027	1	1	2.18E-17
RPL27	2.11E-29	-0.763703401	0.977	1	4.40E-25
NAP1L1	3.91E-17	-0.781757949	0.977	1	8.13E-13
MT-ND4	4.82E-21	-0.783186307	0.977	1	1.00E-16
RPLP2	4.61E-41	-0.806972811	1	1	9.60E-37
AC103591.3	8.53E-07	-0.869189776	0.318	0.579	0.017752112
NFKBIA	1.68E-07	-0.87159047	0.432	0.626	0.003506114
ATP5PO	2.25E-13	-0.879498212	0.682	0.822	4.68E-09
C6orf48	3.93E-20	-0.929248156	0.932	0.897	8.19E-16
JUND	7.55E-14	-0.942526478	0.5	0.822	1.57E-09
RPS11	2.45E-38	-0.956652872	1	1	5.10E-34
RPL38	2.29E-38	-0.966122337	1	1	4.76E-34
FOSB	3.36E-14	-1.021386975	0.523	0.953	6.99E-10
JUN	7.54E-11	-1.024008561	0.636	0.944	1.57E-06
AVP	2.13E-11	-1.063535303	0.705	0.841	4.43E-07
EGR1	9.80E-10	-1.104726945	0.136	0.664	2.04E-05
IER2	5.26E-10	-1.140768076	0.545	0.832	1.10E-05
MT-ND2	7.44E-31	-1.144307278	0.977	1	1.55E-26
ID1	2.65E-10	-1.16867466	0.114	0.654	5.52E-06
JUNB	1.12E-11	-1.17193109	0.591	0.925	2.33E-07
RPL21	2.56E-64	-1.203927866	1	1	5.32E-60
RPS10	8.10E-44	-1.279714793	0.977	1	1.69E-39
ZFP36	3.40E-18	-1.308304151	0.591	0.925	7.09E-14
MALAT1	8.36E-39	-1.355012793	0.977	1	1.74E-34
RPL7	2.83E-68	-1.369439945	1	1	5.88E-64
RPL31	4.43E-71	-1.370424897	1	1	9.22E-67
RPL23	4.50E-50	-1.406556042	1	1	9.36E-46
RPS17	9.50E-45	-1.420319483	0.977	1	1.98E-40
RPS29	4.23E-68	-1.540701551	1	1	8.81E-64
RPL27A	4.18E-69	-1.643786226	0.977	1	8.70E-65
RPL13A	3.49E-83	-1.684605778	1	1	7.27E-79
RPL41	8.02E-89	-1.755627266	1	1	1.67E-84
RPS20	6.35E-82	-2.016040235	0.977	1	1.32E-77

Supplementary Table S2. DEG of HSC-MPP in patients and HCs (Part II: Mild vs HC), related to Fig. 3 & Supplementary Fig. S2

gene	p_val	avg_logFC	pct.1	pct.2	p_val_adj
MT-ATP8	9.45E-32	3.148096779	0.97	0.037	1.97E-27
MT-ND4L	2.62E-47	2.649815635	1	0.626	5.45E-43
EEF1G	5.78E-44	2.518480987	0.939	0.252	1.20E-39
NME2	3.98E-38	2.434982316	1	0.131	8.28E-34
PTPRCAP	5.97E-32	1.610072344	0.97	0	1.24E-27
IFITM1	4.76E-23	1.582682659	0.939	0.065	9.90E-19
MT-ND6	1.06E-16	1.418809221	0.848	0.112	2.21E-12
GABARAP	7.29E-21	1.379255225	0.97	0.159	1.52E-16
ALDOA	2.40E-16	1.139176676	0.909	0.224	5.00E-12
ZFP36L2	1.74E-14	1.135656555	0.97	0.813	3.62E-10
MAT2A	4.02E-18	1.125781799	0.939	0.168	8.37E-14
MIF	6.69E-16	1.081131861	1	0.757	1.39E-11
CD74	2.63E-16	1.068962461	1	0.972	5.47E-12
RNASEK	1.29E-24	1.055991257	0.909	0.019	2.68E-20
PCBP2	7.49E-21	0.996681354	1	0.832	1.56E-16
RPL17	2.24E-24	0.996266659	1	0.981	4.67E-20
PSMA6	8.75E-18	0.978553843	0.909	0.103	1.82E-13
ATP6V0C	4.72E-23	0.948581873	0.848	0.009	9.82E-19
NFE2	4.73E-15	0.942227004	0.879	0.14	9.84E-11
ITGA4	3.16E-16	0.933906235	0.909	0.131	6.57E-12
MYC	1.19E-10	0.932785305	0.667	0.084	2.49E-06
PPIA	5.26E-14	0.913393038	0.97	0.879	1.09E-09
PCBP1	8.94E-14	0.911480173	0.939	0.589	1.86E-09
IFI6	4.27E-12	0.890966909	0.848	0.168	8.89E-08
IFI44L	1.31E-11	0.877869903	0.606	0.037	2.73E-07
HLA-B	1.21E-13	0.83220485	1	0.953	2.53E-09
RCC1	3.97E-12	0.827009336	0.727	0.093	8.27E-08
MT-CO2	6.10E-17	0.826660578	1	1	1.27E-12
SNHG7	5.81E-09	0.818271815	1	0.626	0.000120995
ACTB	1.42E-12	0.813221136	1	0.953	2.96E-08
SERPINB1	1.40E-12	0.811457717	1	0.879	2.92E-08
LY6E	4.04E-09	0.804133529	0.939	0.449	8.42E-05
TRAPPC5	1.52E-20	0.794380436	0.788	0.009	3.17E-16
HLA-C	9.24E-09	0.778467203	0.97	0.888	0.000192423
ZYX	1.97E-09	0.774048606	0.848	0.271	4.10E-05
MATR3.1	1.52E-20	0.767972678	0.788	0.009	3.17E-16
MRPS24	9.67E-20	0.75738023	0.697	0	2.01E-15
MT-ND5	3.29E-11	0.741850619	1	0.981	6.85E-07
PFN1	2.64E-11	0.725674882	1	0.907	5.50E-07
COX16	3.52E-09	0.716736284	0.606	0.075	7.33E-05
BCAP31	1.74E-09	0.708221954	0.879	0.28	3.62E-05
IFITM3	1.64E-06	0.706343666	0.909	0.607	0.03411951
GNG10	1.41E-20	0.700727994	0.818	0.019	2.94E-16
STMN1	4.39E-07	0.694851712	1	0.673	0.009146623
TSPAN3	3.11E-10	0.690960026	0.879	0.252	6.47E-06
VCP	5.13E-08	0.686869659	0.879	0.336	0.001068334
UBE2V1	3.52E-19	0.686678133	0.848	0.047	7.34E-15
RHOA	4.11E-10	0.686630801	0.97	0.673	8.55E-06
HSPD1	4.03E-09	0.682160708	1	0.561	8.39E-05

CRTAP	4.04E-08	0.675417324	0.727	0.178	0.000840934
XAF1	2.07E-10	0.672913891	0.697	0.103	4.31E-06
IL1B	1.05E-10	0.672005561	0.697	0.093	2.19E-06
EPSTI1	6.79E-13	0.669142692	0.697	0.056	1.41E-08
ACTG1	4.45E-12	0.669009663	0.97	0.981	9.26E-08
NRROS	4.32E-11	0.661752939	0.636	0.056	8.98E-07
LSP1	2.25E-06	0.660534585	0.727	0.243	0.046859936
EIF2AK2	6.59E-09	0.654539602	0.848	0.262	0.000137199
ATP5PB	5.11E-07	0.65100468	0.879	0.43	0.010647663
LRRC70	1.13E-17	0.6506908	0.636	0	2.36E-13
MT-ATP6	3.49E-13	0.648159561	1	0.991	7.26E-09
GSTP1	2.00E-09	0.643083615	1	0.888	4.17E-05
NDUFV2	1.40E-07	0.64020953	0.848	0.327	0.002913982
ETV6	9.39E-07	0.638959554	0.727	0.224	0.019543057
DAZAP2	5.28E-08	0.634447317	0.909	0.383	0.001099367
CDK6	4.50E-07	0.633776274	0.97	0.626	0.009378547
HIST1H1E	8.48E-10	0.633470981	0.515	0.028	1.77E-05
HNRNPA2B1	3.81E-08	0.633465965	0.97	0.757	0.000793946
CNBP	4.28E-10	0.632804151	0.97	0.757	8.92E-06
LINC02256	1.14E-11	0.627056102	0.727	0.093	2.37E-07
KDELR1	1.11E-08	0.624244475	0.939	0.402	0.000231536
SNRPA	4.16E-09	0.622638268	0.848	0.252	8.66E-05
RCSD1	1.72E-09	0.618045336	0.939	0.355	3.58E-05
UBE2L6	2.87E-08	0.61031783	0.909	0.355	0.000597242
IMP3	1.73E-08	0.606270908	0.818	0.243	0.000360185
MAT2B	3.75E-12	0.603790256	0.758	0.103	7.82E-08
MBNL1	3.66E-08	0.601036734	0.939	0.411	0.00076297
TOMM5	4.16E-09	0.598658191	0.848	0.252	8.65E-05
TAGLN2	1.25E-07	0.59452423	0.939	0.72	0.00260896
PGD	9.96E-08	0.591792348	0.788	0.243	0.002072983
USP10	2.86E-14	0.590189172	0.788	0.084	5.96E-10
GRINA	5.55E-13	0.589055494	0.788	0.112	1.16E-08
LUC7L3	1.39E-08	0.585076577	0.939	0.393	0.000290038
CDK4	1.76E-06	0.582771095	0.788	0.299	0.036744588
SLC9A3R1	5.17E-10	0.57618802	0.667	0.093	1.08E-05
NOP58	5.94E-08	0.576130521	0.818	0.262	0.001236018
WDR83OS	3.04E-07	0.573812203	1	0.617	0.006326198
SRSF10	1.79E-08	0.57142814	0.909	0.346	0.000371958
RAC2	1.34E-06	0.570885761	0.939	0.654	0.027995675
WASHC1	2.42E-07	0.57070618	0.697	0.178	0.005033349
NUTM2A-AS1	7.02E-08	0.566470895	0.667	0.14	0.001461787
YWHAB	4.60E-07	0.564243159	0.97	0.561	0.009586312
MX1	3.35E-09	0.555110247	0.606	0.075	6.98E-05
RAB37	1.35E-11	0.554359726	0.879	0.215	2.82E-07
ETS2	3.31E-10	0.55393946	0.879	0.252	6.88E-06
ARHGDIB	2.04E-08	0.552888778	1	0.953	0.000425752
HNRNPR	3.69E-09	0.549621438	0.939	0.364	7.68E-05
UROD	3.25E-08	0.542485233	0.818	0.252	0.000676558
NCF4	9.41E-10	0.537311907	0.697	0.121	1.96E-05
CNOT8	7.17E-10	0.53690867	0.606	0.065	1.49E-05
CD82	3.19E-08	0.535545618	0.818	0.252	0.000664388
GLUL	2.01E-06	0.534618145	0.818	0.327	0.041946246
WBP1	9.92E-16	0.534237258	0.576	0	2.07E-11

ANKRD10	1.97E-08	0.527704873	0.727	0.168	0.000410705
ARF5	8.11E-07	0.526455792	0.848	0.346	0.016881519
PGAM1	2.03E-06	0.525995632	0.758	0.262	0.042192856
AC004687.1	7.10E-13	0.525946682	0.879	0.187	1.48E-08
AHCY	3.79E-07	0.521760631	0.758	0.234	0.007891465
CBR1	6.57E-08	0.519552235	0.515	0.056	0.001367099
SLC40A1	1.98E-07	0.518126501	0.727	0.206	0.004116278
PRDX3	1.11E-06	0.516961045	0.788	0.28	0.023099506
ZRANB2	5.59E-09	0.516400254	0.848	0.262	0.0001164
SMARCE1	8.29E-14	0.514721542	0.758	0.084	1.73E-09
SH3BGRL3	1.10E-06	0.510886615	1	0.832	0.022969513
SRPRA	1.93E-08	0.509419713	0.697	0.15	0.000401176
NIFK	1.11E-06	0.508193188	0.667	0.178	0.023162276
MRPS21	7.20E-08	0.506971605	1	0.561	0.001498378
TXNIP	1.19E-06	0.505223491	0.758	0.252	0.024829142
SCHIP1	1.10E-08	0.504869171	0.485	0.037	0.000229247
RPLP0	2.53E-13	0.504642995	1	1	5.26E-09
CAP1	1.66E-08	0.504303081	0.909	0.346	0.000346621
PRKACB	1.07E-08	0.503713132	0.848	0.271	0.00022359
RPN1	1.56E-11	0.501287543	0.788	0.15	3.25E-07
HPS1	4.12E-09	0.500420651	0.667	0.112	8.58E-05
STOM	1.81E-06	0.500376401	0.727	0.234	0.0376651
SCAMP2	4.60E-09	0.499265962	0.758	0.178	9.57E-05
OTUB1	2.76E-10	0.496086347	0.818	0.196	5.75E-06
POLD4	7.30E-10	0.494918338	0.515	0.028	1.52E-05
CAPRIN1	9.95E-08	0.494561117	0.788	0.243	0.002071155
GTF2I	8.68E-08	0.492906285	0.879	0.336	0.001807665
ZNF292	1.53E-08	0.491358704	0.758	0.196	0.000317519
ATP6V0E1	7.23E-08	0.491035414	0.909	0.374	0.00150504
KIF2A	8.70E-10	0.490496384	0.697	0.121	1.81E-05
NDUFV1	2.80E-07	0.486864217	0.818	0.29	0.005836052
ANP32A	4.15E-07	0.48684889	0.879	0.364	0.008639144
ELOVL5	1.04E-07	0.484750052	0.758	0.224	0.002161067
TMC8	1.55E-08	0.484626911	0.758	0.196	0.000321801
GNB5	1.89E-07	0.483017002	0.515	0.065	0.003929486
HLTF	1.24E-07	0.478885019	0.424	0.028	0.00257382
PSMB10	1.68E-08	0.477906517	0.879	0.308	0.000350764
CNPY3	2.40E-07	0.47753864	0.788	0.262	0.004996801
SPI1	1.22E-08	0.477177846	0.697	0.15	0.000253865
MT-CO1	5.24E-08	0.477016881	1	1	0.001090826
ZEB2	5.30E-11	0.476229326	0.879	0.243	1.10E-06
LRRC47	1.40E-07	0.474205728	0.515	0.065	0.002913693
CCT2	8.76E-07	0.474077973	0.97	0.533	0.018245579
FAM208A	3.33E-09	0.473390086	0.515	0.037	6.93E-05
FLI1	1.11E-09	0.473185787	0.848	0.243	2.30E-05
MGLL	1.45E-08	0.472661022	0.667	0.131	0.000302468
SP100	8.23E-09	0.470711947	0.788	0.224	0.000171336
PDCD6	1.82E-06	0.467985392	0.879	0.393	0.037936714
TMEM173	6.10E-09	0.46781911	0.939	0.374	0.00012692
CPXM1	1.32E-07	0.46684017	0.636	0.131	0.002741143
RNF149	3.17E-08	0.465391959	0.455	0.028	0.000659183
MPHOSPH10	5.37E-08	0.46515307	0.697	0.168	0.001117224
RBPMS	2.60E-07	0.462619902	0.727	0.206	0.005412615

SHMT2	8.89E-11	0.461373946	0.667	0.084	1.85E-06
ACTR3	6.43E-07	0.45979213	0.788	0.271	0.013390669
NBPF26	1.39E-10	0.459726828	0.515	0.019	2.89E-06
TMEM246	4.64E-07	0.45896698	0.545	0.093	0.009651882
MRPS18B	1.06E-10	0.458780455	0.697	0.112	2.20E-06
PPP1CA	1.08E-06	0.45594135	0.818	0.318	0.02245465
EMD	4.08E-10	0.451530983	0.727	0.14	8.50E-06
GNAI1	1.45E-06	0.45004447	0.515	0.084	0.030166861
RAB14	4.54E-08	0.449264709	0.636	0.121	0.000945762
TNFRSF1A	2.91E-10	0.448620324	0.818	0.215	6.05E-06
MPIG6B	1.40E-06	0.445724985	0.394	0.037	0.029102116
CHST12	5.54E-07	0.444802705	0.818	0.299	0.011535473
SMARCA4	2.83E-08	0.443685803	0.697	0.159	0.00058901
PTDSS1	3.94E-08	0.443661505	0.788	0.234	0.000820877
RAB24	8.15E-13	0.443560051	0.606	0.028	1.70E-08
TMEM35B	6.79E-14	0.441491413	0.515	0	1.41E-09
HCLS1	1.22E-06	0.441468633	0.697	0.206	0.025436371
CUX1	2.22E-06	0.441323233	0.697	0.215	0.046173928
ATP6V0A2	1.91E-07	0.439325768	0.667	0.159	0.003968247
CEP95	6.09E-08	0.439070938	0.606	0.112	0.001267998
GATD3B	6.05E-10	0.437473398	0.455	0.009	1.26E-05
UQCRC1	7.51E-08	0.437367503	0.697	0.168	0.001563577
MT-CYB	1.30E-08	0.437126349	1	1	0.000270886
MRPL37	6.04E-10	0.436457165	0.697	0.121	1.26E-05
GUCY1B1	6.26E-09	0.43406584	0.576	0.065	0.00013038
HOXA9	3.86E-07	0.433608063	0.606	0.131	0.008042582
GRSF1	8.48E-11	0.432389322	0.758	0.159	1.77E-06
IFIT3	2.61E-11	0.430922659	0.424	0	5.43E-07
PPP2R1A	8.01E-08	0.428930191	0.818	0.29	0.001667268
MRPL38	8.44E-15	0.427688426	0.545	0	1.76E-10
FNTA	7.58E-11	0.426644967	0.667	0.093	1.58E-06
HCG18	1.69E-07	0.426430168	0.545	0.084	0.003525245
VDAC3	8.08E-08	0.426424143	0.879	0.346	0.001682064
ADSL	9.58E-10	0.424367518	0.727	0.159	2.00E-05
STAT5A	2.28E-07	0.42428647	0.788	0.262	0.004757343
C11orf98	1.19E-11	0.423816194	0.515	0.009	2.48E-07
RCN1	1.67E-06	0.423363632	0.667	0.187	0.034668295
MRPL18	9.59E-09	0.422940952	0.667	0.131	0.000199671
TUT4	2.09E-07	0.422382371	0.576	0.103	0.004357162
SDHA	1.05E-10	0.422243923	0.697	0.112	2.18E-06
FDFT1	2.22E-07	0.421678196	0.727	0.215	0.004629761
KDM5B	5.04E-07	0.421471303	0.515	0.075	0.010493442
SERINC3	6.80E-07	0.421128634	0.697	0.206	0.014164138
MGMT	2.35E-10	0.418100364	0.818	0.215	4.90E-06
TECR	8.80E-09	0.418028531	0.818	0.252	0.000183239
ISYNA1	8.84E-08	0.417532012	0.727	0.196	0.001839567
GCA	1.05E-08	0.414760465	0.515	0.047	0.000218604
GRB2	1.58E-08	0.414719167	0.697	0.159	0.000328677
TBC1D5	9.70E-09	0.414511794	0.606	0.103	0.000201879
OAS1	8.94E-11	0.412371034	0.485	0.009	1.86E-06
CDIPT	3.83E-09	0.410492004	0.727	0.178	7.96E-05
TRIM58	1.90E-11	0.409292578	0.636	0.075	3.96E-07
CNN2	1.43E-09	0.40888641	0.818	0.243	2.99E-05

FAM219B	3.50E-07	0.408207177	0.576	0.112	0.007285946
LCP2	4.34E-10	0.406436629	0.727	0.15	9.03E-06
DOK3	2.45E-07	0.403666124	0.515	0.075	0.005099158
AC116366.3	1.27E-07	0.399033481	0.364	0.009	0.002643518
SRSF1	1.36E-07	0.396718423	0.667	0.168	0.002839891
SMARCC2	5.74E-08	0.395438557	0.606	0.121	0.001195568
SEC13	6.21E-07	0.393887243	0.606	0.14	0.012934717
PIAS1	4.04E-07	0.393811774	0.636	0.15	0.008407601
MPV17	1.16E-07	0.39233405	0.606	0.131	0.00241211
DDX39B	4.16E-10	0.392155795	0.576	0.056	8.66E-06
FAM96A	7.11E-07	0.392021445	0.758	0.262	0.014797757
ZNF706	4.74E-07	0.391580547	0.879	0.374	0.009872692
ARPC5	4.87E-07	0.390936799	0.879	0.383	0.01013048
NDUFAF3	3.14E-07	0.390578005	0.818	0.308	0.006540805
MTG1	3.81E-09	0.390558403	0.424	0.009	7.93E-05
CBWD2	8.93E-07	0.389270677	0.424	0.047	0.018592246
LBR	9.97E-07	0.389074018	0.606	0.14	0.020766856
BOD1L1	4.28E-07	0.387968885	0.606	0.14	0.008907042
PTPN6	1.94E-06	0.387538268	0.788	0.299	0.040472902
TCF12	1.46E-09	0.386759428	0.545	0.047	3.03E-05
TMOD3	5.77E-10	0.38646004	0.515	0.037	1.20E-05
CXXC5	6.39E-07	0.386373839	0.606	0.14	0.01329867
M6PR	4.11E-13	0.386038665	0.879	0.215	8.55E-09
GPS1	6.65E-08	0.386007521	0.545	0.084	0.001383839
IRF2	9.90E-08	0.385878143	0.515	0.065	0.00206104
SQSTM1	1.60E-07	0.385228907	0.848	0.327	0.003324767
RFTN1	7.36E-07	0.385193682	0.606	0.14	0.015331702
U2AF1	2.30E-08	0.385141139	0.485	0.037	0.000478191
COQ8A	2.40E-08	0.385111986	0.576	0.084	0.000498881
GOLPH3	5.41E-07	0.385009585	0.697	0.206	0.011267898
G3BP1	6.29E-08	0.383473412	0.788	0.252	0.001310203
NMI	4.71E-08	0.382323365	0.758	0.224	0.00098021
SLC9B2	3.74E-09	0.381617754	0.424	0.009	7.79E-05
RAB7A	1.12E-06	0.380849575	0.727	0.243	0.023288207
ATP8B4	8.20E-08	0.380075541	0.606	0.131	0.00170695
EIF4EBP3	2.25E-11	0.379379511	0.485	0.019	4.68E-07
HIST2H2AA4	9.47E-07	0.379161459	0.364	0.019	0.019719434
P4HB	1.19E-06	0.378632487	0.788	0.299	0.024677131
JUP	6.31E-08	0.378034877	0.697	0.178	0.00131339
TIA1	1.57E-09	0.37781076	0.636	0.103	3.26E-05
DUSP23	9.39E-07	0.377283256	0.788	0.318	0.019547156
CAPG	1.20E-07	0.376590776	0.636	0.15	0.002493522
RPN2	8.10E-07	0.375210583	0.848	0.364	0.016869879
HACD4	5.34E-08	0.374647771	0.727	0.206	0.001111235
CNST	1.73E-07	0.37424028	0.606	0.131	0.00359967
NCBP2-AS2	3.66E-08	0.372656338	0.697	0.178	0.000762319
BSG	1.05E-06	0.372415956	0.818	0.327	0.02190626
IMMT	1.43E-10	0.372038461	0.697	0.131	2.99E-06
PFKL	3.47E-07	0.371993305	0.545	0.093	0.007214832
PAK2	3.05E-08	0.371828577	0.818	0.28	0.000635572
CYC1	8.19E-07	0.371315866	0.788	0.29	0.017058434
TMEM106B	5.12E-08	0.370923592	0.515	0.056	0.001066076
GPI	1.81E-06	0.370591298	0.606	0.15	0.037682934

SCOC	3.13E-08	0.370530164	0.576	0.103	0.000652284
NOL7	2.56E-08	0.369929681	0.909	0.383	0.000532428
CUEDC2	1.87E-06	0.369787451	0.727	0.243	0.038846405
ATP6AP1	5.58E-08	0.369777392	0.606	0.112	0.001160868
VPS36	8.72E-10	0.368348098	0.788	0.215	1.81E-05
SYNGR2	2.47E-08	0.36747194	0.636	0.131	0.000514616
RP9	1.35E-07	0.366158607	0.455	0.037	0.00280136
CD46	3.17E-09	0.365590805	0.576	0.084	6.59E-05
SNRNP40	1.33E-08	0.365126722	0.636	0.121	0.00027751
MFSD1	2.59E-07	0.364142145	0.697	0.196	0.005384108
TACC3	8.07E-07	0.364094906	0.636	0.168	0.01680738
KCNAB2	6.54E-08	0.363951787	0.667	0.159	0.001362406
DRG1	2.17E-07	0.36229725	0.667	0.168	0.004512653
NLRC3	1.81E-07	0.360887466	0.394	0.019	0.003769615
ADH5	3.22E-08	0.360581371	0.667	0.159	0.000669799
AGGF1	1.15E-12	0.360570562	0.545	0.019	2.40E-08
FAM110A	1.62E-07	0.360540261	0.424	0.028	0.003373037
ARL5A	7.17E-10	0.35994612	0.667	0.121	1.49E-05
UBE2I	1.35E-07	0.359842536	0.909	0.402	0.002817003
MRPL40	1.88E-10	0.358501552	0.636	0.093	3.92E-06
HIST1H2BK	3.76E-12	0.35822478	0.455	0	7.82E-08
UBR5-AS1	2.68E-07	0.358000093	0.515	0.075	0.005580058
HLA-F	1.01E-09	0.356913001	0.636	0.112	2.09E-05
PLXND1	1.45E-08	0.356321794	0.455	0.028	0.000301188
CMTM3	1.93E-06	0.356167947	0.545	0.112	0.040234723
NOSIP	2.94E-09	0.356142525	0.727	0.178	6.13E-05
NAPA	1.15E-06	0.355918333	0.576	0.131	0.023948697
PPIH	4.57E-08	0.355913429	0.636	0.131	0.00095049
NGRN	5.82E-10	0.355750282	0.455	0.009	1.21E-05
SMIM3	6.60E-08	0.355331231	0.727	0.224	0.001373876
DUS3L	2.52E-07	0.355215968	0.485	0.056	0.005239584
PCMTD1	9.55E-09	0.354774417	0.697	0.168	0.000198928
ARRDC1	3.39E-11	0.354582502	0.667	0.103	7.05E-07
HSPB11	8.21E-08	0.352326555	0.667	0.178	0.001708287
HAUS4	1.14E-06	0.351798201	0.485	0.075	0.023809015
REXO2	1.23E-08	0.351422189	0.788	0.252	0.000256394
DPYD	1.08E-07	0.350974549	0.545	0.084	0.002248355
LINC01684	4.47E-11	0.350746284	0.515	0.019	9.31E-07
RNF167	7.09E-07	0.350349074	0.727	0.234	0.014761262
WDR1	9.06E-11	0.349953137	0.758	0.187	1.89E-06
LRPPRC	5.82E-09	0.349886602	0.576	0.084	0.000121124
RGS19	2.12E-07	0.349648794	0.727	0.224	0.004406366
HMG20B	1.55E-06	0.349217712	0.606	0.159	0.032238804
UBA3	1.45E-07	0.349099272	0.455	0.047	0.003024037
SLC25A46	8.74E-07	0.348490118	0.394	0.028	0.018206232
ELAC2	1.10E-09	0.34806636	0.485	0.028	2.30E-05
HMGN4	5.18E-13	0.347840459	0.485	0	1.08E-08
SMARCB1	2.79E-09	0.346912085	0.788	0.234	5.82E-05
GSTM2	1.61E-10	0.346055674	0.485	0.019	3.36E-06
MZT2A	2.65E-07	0.344747963	0.879	0.383	0.005527117
STX10	2.24E-07	0.344599882	0.697	0.196	0.004672909
FPGS	4.85E-10	0.344051652	0.576	0.065	1.01E-05
ABCB7	5.46E-07	0.344022293	0.545	0.103	0.011366891

UBXN4	4.05E-07	0.343300381	0.848	0.355	0.008426354
SYK	2.04E-06	0.343224826	0.606	0.168	0.042414316
AASDHPPT	9.92E-09	0.343037763	0.515	0.056	0.000206603
SWAP70	1.76E-06	0.342910511	0.515	0.093	0.036612121
FBXL5	2.54E-07	0.340737343	0.576	0.112	0.005290605
DNAJA1	3.71E-08	0.340689663	0.879	0.346	0.000772325
ZNF524	4.74E-07	0.340249165	0.394	0.028	0.009869936
ENOSF1	1.69E-06	0.340157148	0.545	0.121	0.035171235
HSD17B4	5.75E-09	0.340103263	0.515	0.056	0.000119804
HNRNPAB	1.55E-06	0.33967145	0.636	0.187	0.03217568
ANXA6	1.19E-06	0.33943227	0.576	0.131	0.024835028
IVD	6.66E-08	0.338988511	0.455	0.037	0.001387619
AL365205.1	1.43E-09	0.33826081	0.424	0.009	2.98E-05
ITGA2B	2.36E-08	0.338097349	0.394	0.009	0.000490608
TAGAP	1.26E-06	0.336805174	0.424	0.047	0.02624134
AGPAT5	3.83E-09	0.336133489	0.515	0.047	7.98E-05
MRPL51	5.44E-09	0.335826983	0.909	0.374	0.000113255
MAGEF1	2.08E-10	0.335769349	0.606	0.075	4.34E-06
EIF4E	1.09E-06	0.335161646	0.667	0.196	0.02273265
ITGA5	9.17E-08	0.335055145	0.455	0.037	0.001908974
METAP1	8.29E-09	0.334382543	0.515	0.047	0.00017254
WSB1	2.06E-06	0.334023561	0.909	0.449	0.042815317
PPP6R2	9.10E-08	0.333826974	0.515	0.065	0.001895602
PLAGL1	9.08E-08	0.333191418	0.667	0.187	0.00189074
PNPLA2	2.15E-06	0.332473073	0.636	0.187	0.044758828
HERC4	4.67E-07	0.332285801	0.545	0.103	0.009720615
SDHD	5.84E-10	0.332051556	0.727	0.178	1.22E-05
TTC17	2.05E-06	0.331103135	0.667	0.196	0.042705042
PUM2	2.11E-08	0.330450343	0.576	0.093	0.000438402
HEBP1	1.29E-07	0.330104201	0.879	0.374	0.002687001
BCCIP	6.75E-12	0.329587633	0.758	0.168	1.40E-07
DEF6	5.11E-07	0.329120195	0.545	0.112	0.010634477
TIPRL	6.29E-07	0.328449039	0.576	0.131	0.013101941
FLOT2	1.97E-06	0.327622572	0.485	0.084	0.040976517
LTBR	2.26E-08	0.325875611	0.576	0.093	0.000469963
BTK	5.10E-09	0.325155413	0.636	0.121	0.000106208
OAS2	4.17E-09	0.324732941	0.455	0.019	8.68E-05
SCRN2	9.95E-09	0.324218341	0.667	0.15	0.000207228
TMEM9	3.58E-07	0.32400237	0.545	0.112	0.007452651
RANBP2	1.87E-06	0.323565418	0.576	0.14	0.038976962
WASF1	9.61E-07	0.323126123	0.545	0.121	0.020012471
LAIR1	3.54E-09	0.323120125	0.818	0.262	7.36E-05
DPH1	1.73E-10	0.322158067	0.394	0	3.61E-06
APH1A	1.12E-07	0.322039989	0.879	0.374	0.002325192
SF3B4	2.40E-06	0.321722235	0.636	0.187	0.049910455
PUF60	1.93E-07	0.320248592	0.727	0.234	0.004014799
ZBTB44	7.35E-09	0.319501239	0.606	0.112	0.00015311
LCP1	4.85E-08	0.318612423	0.818	0.299	0.001010273
SYTL1	1.03E-07	0.318566593	0.727	0.234	0.002150915
PARP10	8.85E-11	0.318298689	0.515	0.028	1.84E-06
HSPA4	5.24E-09	0.317589307	0.545	0.065	0.000109152
KAT8	2.51E-09	0.316757569	0.606	0.112	5.23E-05
SARNP	3.06E-09	0.316718039	0.485	0.037	6.37E-05

KMT2C	4.59E-09	0.315696239	0.606	0.112	9.56E-05
ZKSCAN1	2.36E-06	0.31550819	0.727	0.262	0.049068514
C20orf27	9.76E-07	0.315452417	0.727	0.252	0.020327747
ARHGEF1	7.06E-08	0.315352498	0.667	0.178	0.001469654
MED28	9.36E-07	0.315340976	0.788	0.308	0.019486331
ADSS	4.89E-07	0.315094855	0.606	0.159	0.010178826
TNFSF13	1.73E-10	0.314565248	0.394	0	3.61E-06
TESMIN	2.79E-10	0.313422719	0.606	0.075	5.81E-06
WASHC3	1.23E-07	0.312973401	0.606	0.131	0.002565174
ILVBL	8.39E-10	0.312462081	0.636	0.112	1.75E-05
CLTC	1.11E-06	0.312380712	0.455	0.065	0.02303329
BRIX1	1.43E-06	0.310754784	0.515	0.103	0.029811546
NIPSNAP2	6.30E-07	0.310602544	0.576	0.131	0.013122244
NMRAL1	1.03E-08	0.310304818	0.697	0.178	0.000213586
PRRC2B	4.65E-08	0.310279121	0.545	0.093	0.000967971
BUB3	1.55E-12	0.30985522	0.758	0.159	3.22E-08
MYCBP2	2.29E-06	0.309708164	0.758	0.299	0.047705071
MTA1	7.21E-07	0.309323365	0.606	0.15	0.015018346
FSCN1	2.71E-07	0.309247726	0.636	0.168	0.005646014
MRPL3	1.02E-09	0.309138733	0.727	0.178	2.13E-05
RNF24	1.26E-08	0.308721317	0.606	0.131	0.000263115
FARSA	2.29E-09	0.307649131	0.606	0.103	4.77E-05
RNF220	7.52E-08	0.307570908	0.697	0.206	0.001565405
CHD2	2.27E-07	0.305814257	0.636	0.168	0.004731369
RPL7A	5.57E-09	0.305504329	1	1	0.000116015
NT5C2	2.56E-07	0.305408326	0.515	0.084	0.005323603
CTSB	4.05E-07	0.304899266	0.515	0.103	0.008431173
DELE1	1.38E-06	0.304399727	0.545	0.121	0.028677497
ORAI3	5.05E-09	0.304165113	0.455	0.037	0.000105194
MLEC	1.35E-08	0.303653792	0.667	0.159	0.000281973
RPRD1A	1.08E-08	0.302240323	0.576	0.093	0.00022407
OXLD1	7.43E-07	0.302165266	0.576	0.131	0.015479134
TM9SF2	2.58E-07	0.30209726	0.545	0.112	0.005380012
RNF34	4.07E-09	0.301870809	0.424	0.019	8.47E-05
CLN6	1.73E-10	0.301810381	0.394	0	3.61E-06
SNX17	1.50E-08	0.301766737	0.758	0.243	0.000312846
MCM5	7.81E-07	0.301547078	0.515	0.103	0.016259407
TTC5	5.63E-07	0.301092945	0.485	0.084	0.011727568
AC243960.1	2.34E-11	0.300634388	0.455	0.009	4.87E-07
STAT6	1.75E-06	0.299613772	0.485	0.084	0.036394392
COA5	2.00E-08	0.299205582	0.636	0.15	0.000415802
AUP1	1.46E-06	0.299072114	0.788	0.327	0.030304994
MRTO4	1.88E-06	0.298621023	0.485	0.084	0.039090055
RPL30	6.68E-07	0.298345504	1	1	0.013911616
MALRD1	6.83E-09	0.298047794	0.333	0	0.000142106
AARSD1	9.07E-09	0.297550788	0.455	0.028	0.00018892
CHTF8	2.23E-07	0.297069115	0.545	0.103	0.00463502
ELP5	1.24E-06	0.296631332	0.485	0.084	0.025749613
USP39	4.36E-07	0.296167623	0.485	0.075	0.009072825
ADAM15	3.39E-07	0.295595195	0.485	0.065	0.007048994
METTL26	6.88E-10	0.295506425	0.909	0.355	1.43E-05
EPN1	9.03E-08	0.295504231	0.515	0.093	0.001879125
RPP21	3.99E-08	0.294773635	0.455	0.037	0.000831158

LRRC41	3.23E-08	0.294433006	0.576	0.103	0.000672666
GPAT3	1.85E-07	0.294331031	0.485	0.075	0.003845345
STIM1	1.49E-08	0.294302858	0.515	0.065	0.000310739
STAT1	3.69E-08	0.293889991	0.515	0.075	0.00076766
PDCD6IP	1.99E-06	0.293499566	0.545	0.14	0.041507156
SENP6	3.21E-09	0.293343718	0.667	0.14	6.69E-05
RAB11B	1.26E-06	0.292935144	0.667	0.215	0.026146948
MRPS26	6.55E-09	0.292585553	0.576	0.103	0.000136445
ARFGAP2	1.42E-07	0.292099442	0.636	0.159	0.002949286
SLC38A10	1.79E-07	0.290686287	0.455	0.047	0.00373527
MTCH1	1.95E-07	0.289424729	0.636	0.178	0.004063786
MFSD14A	1.11E-09	0.289344161	0.364	0	2.31E-05
SMIM10L1	1.23E-06	0.28927528	0.758	0.299	0.025581016
COQ9	2.65E-07	0.288831872	0.515	0.084	0.005517107
NAP1L4	2.32E-07	0.28881857	0.636	0.168	0.004826354
STRADB	2.90E-07	0.288740228	0.455	0.047	0.006041833
MRPS23	8.97E-07	0.288367657	0.576	0.14	0.01867122
PTTG1IP	2.55E-07	0.286955008	0.667	0.196	0.005301697
ANKRD17	1.09E-06	0.286932603	0.576	0.14	0.022625303
NUPL2	5.68E-07	0.286192569	0.515	0.093	0.011823903
ITFG1	2.26E-06	0.286038402	0.515	0.103	0.047047142
BCKDHA	4.06E-08	0.285344722	0.303	0	0.000845506
MTHFD2L	7.31E-09	0.285280513	0.697	0.178	0.00015217
PSMD3	7.17E-10	0.285220487	0.636	0.121	1.49E-05
SF3A2	1.01E-06	0.284880253	0.667	0.215	0.02099866
USP4	5.44E-07	0.284309742	0.515	0.093	0.011335145
GMDS	1.60E-06	0.28387037	0.455	0.065	0.033299793
RAB5B	1.49E-06	0.283249291	0.424	0.047	0.031030781
ARID1A	1.74E-08	0.283222918	0.636	0.159	0.000363154
SLC38A2	3.15E-10	0.283096405	0.788	0.224	6.57E-06
AL034397.3	4.31E-07	0.283031792	0.485	0.084	0.008967589
NSL1	2.78E-08	0.282833106	0.515	0.075	0.000578069
SCMH1	1.36E-07	0.282349299	0.424	0.037	0.002835815
RPUSD1	3.97E-08	0.281983283	0.485	0.056	0.000827069
DPY19L2	1.13E-07	0.281910672	0.364	0.009	0.002359769
UCKL1	3.00E-07	0.281909291	0.424	0.037	0.006253453
IL16	4.87E-07	0.281789678	0.515	0.103	0.010131152
RAB6A	3.18E-07	0.28145094	0.485	0.075	0.006610384
MAX	3.46E-07	0.281261386	0.848	0.374	0.007211154
SNAP23	5.60E-07	0.281009261	0.545	0.131	0.011668042
ZNF738	1.37E-06	0.279713716	0.394	0.037	0.028474714
N6AMT1	4.30E-07	0.279397783	0.333	0.009	0.0089511
MRPS12	8.06E-08	0.279348943	0.636	0.168	0.001677715
PPM1K	2.03E-06	0.279121688	0.424	0.056	0.042213929
HIGD1A	1.62E-07	0.278961609	0.576	0.14	0.00336902
RWDD3	4.06E-08	0.27895453	0.303	0	0.000845506
TNFAIP8L2	6.83E-09	0.27874432	0.333	0	0.000142106
TRAPPC6B	2.77E-07	0.278728461	0.424	0.047	0.005763513
PTBP1	1.79E-06	0.276293749	0.636	0.206	0.037170929
CIAO1	8.88E-07	0.275918795	0.455	0.065	0.018483547
PRMT2	1.58E-06	0.275348699	0.667	0.224	0.032949193
NSMAF	1.34E-07	0.275098236	0.576	0.121	0.002789439
DCAF7	1.02E-06	0.274692961	0.667	0.224	0.021135647

SECISBP2	4.91E-07	0.274437937	0.636	0.187	0.010213626
TPRA1	9.51E-08	0.274029184	0.455	0.047	0.001979818
TMEM115	4.92E-07	0.27396855	0.364	0.019	0.010240877
KIAA1468	2.34E-07	0.273397502	0.273	0	0.004872208
TBP	4.74E-07	0.2733519	0.424	0.037	0.009859775
SLC39A8	5.09E-07	0.273296137	0.545	0.121	0.010588451
RBM42	6.41E-07	0.272290189	0.576	0.15	0.013339394
NAT10	1.08E-06	0.272193408	0.394	0.037	0.022390996
CYB5R1	7.08E-07	0.272064727	0.455	0.065	0.014737982
MAD2L2	2.15E-07	0.27129807	0.545	0.103	0.004485947
THOC6	2.38E-06	0.2712706	0.485	0.103	0.049616671
KPNB1	4.70E-07	0.270987945	0.788	0.308	0.009783917
DNAJA2	1.12E-09	0.269792205	0.576	0.084	2.34E-05
COPB1	5.46E-07	0.269787599	0.455	0.075	0.011358449
SIGIRR	1.01E-06	0.268711993	0.636	0.196	0.021076625
ASF1A	1.39E-06	0.268379916	0.485	0.075	0.028943141
ISCU	1.37E-06	0.268149298	0.818	0.364	0.028459403
BSCL2	7.50E-08	0.267805667	0.394	0.019	0.001562451
DBNDD2	4.06E-08	0.266938174	0.303	0	0.000845506
CHMP1A	2.05E-08	0.266718485	0.455	0.037	0.000427772
RAB2A	1.57E-06	0.266555964	0.697	0.243	0.032643117
GOLGA7	7.04E-08	0.266481356	0.576	0.14	0.0014648
ASNA1	1.50E-07	0.266153755	0.545	0.121	0.00312638
PIGM	2.10E-06	0.265803074	0.424	0.065	0.043699899
DCTN4	1.64E-09	0.265059515	0.606	0.112	3.41E-05
MMRN1	8.49E-07	0.264523881	0.576	0.159	0.017675093
SUPT6H	1.71E-06	0.26440106	0.455	0.075	0.035581744
TRIM27	1.87E-10	0.264341989	0.515	0.056	3.88E-06
EGLN2	8.21E-08	0.264094266	0.364	0.009	0.001708847
VPS4A	2.54E-08	0.263753969	0.545	0.103	0.00052898
MAN2A2	1.51E-07	0.263523232	0.485	0.084	0.003140653
SSNA1	8.19E-07	0.263035817	0.758	0.299	0.017056809
DTX3	4.04E-10	0.262625465	0.576	0.084	8.41E-06
COPB2	1.11E-08	0.261774796	0.545	0.093	0.000230876
SURF4	7.50E-08	0.261608661	0.576	0.131	0.001562019
SNX20	2.30E-07	0.261576626	0.424	0.037	0.004779174
PARP9	3.27E-07	0.261386196	0.424	0.047	0.006805811
GET4	1.11E-09	0.261332995	0.364	0	2.31E-05
MAN1A2	1.46E-08	0.261007959	0.606	0.15	0.000303101
EDEM2	1.77E-07	0.260815515	0.424	0.047	0.003690914
ABHD10	1.69E-07	0.26067421	0.455	0.056	0.003510214
SEC14L1	1.56E-06	0.260662652	0.485	0.103	0.032440027
MKL1	1.08E-07	0.25934714	0.364	0.009	0.002255846
WDR77	6.45E-07	0.25813476	0.364	0.019	0.013432249
RPUSD4	1.68E-07	0.257337433	0.485	0.075	0.003492209
CALCOCO2	1.81E-07	0.257186749	0.515	0.103	0.003777765
MRPS27	4.06E-09	0.256835147	0.667	0.168	8.46E-05
DAP	1.58E-06	0.256833487	0.576	0.159	0.032821509
PSMF1	5.97E-07	0.25672679	0.758	0.29	0.012420446
SLBP	2.19E-06	0.256306655	0.606	0.187	0.045698471
AKIRIN2	2.16E-06	0.256193599	0.455	0.075	0.04502529
PRPF19	1.11E-07	0.255200189	0.545	0.112	0.002317304
ACY1	9.71E-08	0.255049047	0.364	0.009	0.002020716

RRAGA	8.65E-08	0.254483576	0.545	0.112	0.001800759
ALDH9A1	6.20E-07	0.254107521	0.545	0.131	0.012901199
RAB34	2.62E-07	0.253478338	0.727	0.28	0.005457713
SIGMAR1	2.25E-07	0.253443196	0.424	0.037	0.004694679
LEMD3	2.36E-06	0.252612836	0.364	0.028	0.049096204
FUNDC2	1.88E-08	0.252119963	0.758	0.262	0.000392054
WASHC4	2.59E-09	0.251286557	0.576	0.093	5.40E-05
SCAMP3	1.07E-06	0.251183773	0.485	0.093	0.022245249
TMC6	2.52E-08	0.250969785	0.515	0.075	0.000524037
YME1L1	3.70E-07	0.250400065	0.697	0.234	0.007710734
RPL26	4.32E-09	-0.25896387	1	1	8.99E-05
DDX3X	2.82E-07	-0.29995192	0.818	0.617	0.005863225
RPL18A	1.29E-08	-0.30076362	1	1	0.000269276
RPS27A	8.52E-11	-0.30910826	1	1	1.77E-06
RPS14	5.98E-11	-0.31487972	1	1	1.24E-06
RPL39	3.25E-10	-0.31603812	1	1	6.77E-06
RPS19	1.92E-09	-0.32628917	1	1	4.00E-05
UBA52	1.63E-07	-0.36142176	1	1	0.003392352
RPL36	3.46E-11	-0.37110507	1	1	7.20E-07
RPL15	6.24E-14	-0.39590687	1	1	1.30E-09
RPL3	3.22E-14	-0.39860158	1	1	6.71E-10
RPS16	1.41E-06	-0.43529221	1	1	0.029432029
RPL10A	1.53E-13	-0.43630838	0.97	1	3.18E-09
RPS18	1.53E-17	-0.4637237	1	1	3.19E-13
SNRPE	1.85E-06	-0.46630499	0.909	0.832	0.038462359
SRSF6	1.72E-07	-0.51344515	0.394	0.523	0.003584333
HNRNPA1P48	5.99E-07	-0.52907987	0	0.374	0.012461111
UQCRB	2.14E-07	-0.54511246	0.909	0.981	0.004456575
RPL34	1.16E-21	-0.56164783	1	1	2.42E-17
RPL35	2.41E-22	-0.58913273	1	1	5.01E-18
RPS6	2.54E-24	-0.60141869	1	1	5.29E-20
SNHG8	4.12E-07	-0.60254829	0.909	0.981	0.008575499
EIF1	5.50E-10	-0.609737	1	1	1.15E-05
FTH1	4.81E-09	-0.669021	1	0.991	0.00010013
RPL23A	1.54E-28	-0.71010185	1	1	3.22E-24
RPL27	7.02E-23	-0.71935875	1	1	1.46E-18
IDS	4.91E-08	-0.72268188	0.697	0.822	0.001022395
MT-ND4	2.42E-18	-0.75877726	1	1	5.03E-14
NAP1L1	1.35E-14	-0.76531885	1	1	2.80E-10
JUND	3.47E-08	-0.765713	0.485	0.822	0.0007234
RPL9	6.45E-37	-0.80076841	1	1	1.34E-32
RPL37A	1.09E-31	-0.84195829	0.939	1	2.28E-27
CHMP1B	1.68E-07	-0.86148334	0.273	0.617	0.00348755
RPLP2	4.50E-39	-0.86540181	1	1	9.37E-35
RPS27	9.79E-36	-0.90505298	1	1	2.04E-31
RPL36A	1.97E-22	-0.91282663	0.97	1	4.10E-18
FOS	4.01E-20	-0.94360344	0.758	0.991	8.34E-16
IER2	5.37E-07	-0.94924349	0.606	0.832	0.011182579
ATP5PO	6.17E-12	-1.04213231	0.515	0.822	1.28E-07
RPS11	5.79E-33	-1.06387153	0.97	1	1.21E-28
JUN	1.01E-10	-1.06398396	0.636	0.944	2.10E-06
AC103591.3	7.32E-08	-1.07370333	0.242	0.579	0.001523238
AVP	3.12E-08	-1.08268474	0.576	0.841	0.000649202

MCL1	7.94E-09	-1.1020473	0.545	0.766	0.000165333
MT-ND2	3.60E-29	-1.2001728	1	1	7.49E-25
JUNB	4.31E-10	-1.21206396	0.545	0.925	8.97E-06
RPL38	6.12E-37	-1.22181931	0.939	1	1.28E-32
ID1	4.26E-09	-1.24060215	0.091	0.654	8.86E-05
MALAT1	3.03E-32	-1.26043166	1	1	6.30E-28
RPL23	1.14E-42	-1.3062287	1	1	2.38E-38
C6orf48	9.19E-21	-1.30831934	0.727	0.897	1.91E-16
EGR1	2.90E-09	-1.31365587	0.121	0.664	6.04E-05
FOSB	5.60E-17	-1.31576769	0.273	0.953	1.17E-12
RPS10	5.48E-40	-1.3292192	0.97	1	1.14E-35
RPS17	1.64E-34	-1.36101535	0.939	1	3.42E-30
RPL21	4.79E-63	-1.38282392	1	1	9.97E-59
ZFP36	2.74E-15	-1.38805343	0.394	0.925	5.70E-11
RPL7	1.46E-65	-1.42803133	1	1	3.04E-61
RPL31	5.34E-66	-1.57227555	1	1	1.11E-61
RPS29	3.20E-56	-1.70420818	1	1	6.67E-52
RPL27A	1.34E-66	-1.82400713	0.909	1	2.80E-62
RPL41	1.20E-74	-1.86631515	1	1	2.50E-70
RPL13A	2.81E-82	-1.88293633	1	1	5.86E-78
RPS20	1.38E-83	-2.30920625	0.97	1	2.88E-79

Supplementary Table S3. Lineage specific markers

Cell types	Markers
HSC/MPP	CRHBP, MYCT1, MLLT3, HLF, BEX2, NPR3, AVP, IDS
B-like	CD24, AKAP12, VPREB1, CD9, CD79B, EBF1, DNTT, MME, CD79A
T-like	IL32, NKG7, GZMH, KLRF1, GNLY, GZMA, PRF1
GMP	MPO, CTSG, ELANE, AZU1, PRTN3, TNFSF13B, CSTA, CFD
EBMP	HDC, LMO4, MS4A2, CLC, CPA3
EP	KCNH2, UROD, BLVRB, HBD, KLF1, CA1, CA2
